# Catalyst-Free One-Pot Synthesis of Densely Substituted Pyrazole-Pyrazines as Anti-Colorectal Cancer Agents

**DOI:** 10.1038/s41598-020-66137-z

**Published:** 2020-06-09

**Authors:** Jia Xu, Hong-Bo Tan, Ya-Jun Zhang, Dian-Yong Tang, Fenghuang Zhan, Hong-yu Li, Zhong-Zhu Chen, Zhi-Gang Xu

**Affiliations:** 1 College of Pharmacy, National & Local Joint Engineering Research Center of Targeted and Innovative Therapeutics, Chongqing Key Laboratory of Kinase Modulators as Innovative Medicine, Chongqing University of Arts and Sciences, Chongqing, 402160 China; 2 Department of Pharmaceutical Sciences, College of Pharmacy, University of Arkansas for Medical Sciences, Little Rock, AR 72205 USA; 3 Winthrop P. Rockefeller Cancer Institute, University of Arkansas for Medical Sciences, Little Rock, AR 72205 USA

**Keywords:** Green chemistry, Medicinal chemistry, Organic chemistry, Combinatorial libraries, Synthetic chemistry methodology, Microwave chemistry

## Abstract

The first catalyst-free post-Ugi cascade methodology was developed for expeditious access to structurally diverse and complex pyrazole-pyrazines in one-pot. This novel cascade reaction features an intramolecular *N2*-arylation of pyrazoles with allenes at the C-β position of triple bond. Screening in the colorectal cancer cell lines HCT116 and SW620 validated the feasibility of the methodology for generating bioactive compounds. The lead compound **7h** which is active against HCT116 and SW620 with IC_50_ of 1.3 and 1.8 µM, respectively, can be synthesized and purified in a gram process synthetic scale in 7 hours. The mechanical studies indicated that compound **7h** can induce cell cycle arrest in the G2/M phase and inhibit proliferation and viability in human colon cancer cells. Overall, compound **7h** is represented as a promising starting point for the development of new anti-colorectal cancer drugs.

## Introduction

The Ugi four-component reaction (U-4CR) as a typical multicomponent reaction (MCR) continues to attract considerable interest^1^. The post-Ugi condensation is known as a versatile and highly efficient synthetic strategy for the preparation of heterocyclic compounds^2–4^. In addition, the Ugi MCR provides expeditious access to complex and drug like chemical scaffolds with high iterative efficiency from commercially available starting materials^5–8^.

**Figure 1:**
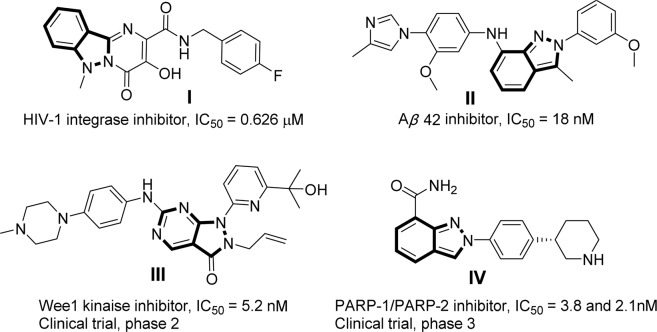
Bioactive structures of 2*H*-indazoles.

In recent years, the indazole scaffold as an important core structure has been investigated extensively as antibacterial agent^9,10^, HIV-1 integrase inhibitor (**I**)^11^, *γ*-secretase modulator (GSM) (**II**)^12^, Wee1 kinase inhibitor (**III**)^13,14^ and PARP-1/PARP-2 inhibitor (**IV**) for the treatment of cancer (Fig. 1)^15,16^.

Although synthetic studies of indazole derivatives have been mainly devoted to the intramolecular *N*1-arylation with metal catalysts^17–19^, the intramolecular *N*2-arylation was also feasible^20,21^. More recently, privileged scaffolds represented by 2*H*-indazole were prepared via the U-4CR with Au@Al-SBA15^17^, silver^19^, or copper as a catalyst^22–24^. In view of the cost-effective and environmentally friendly methodologies developed with MCRs for the synthesis of heterocyclic compounds^25–27^, further exploring sustainable reaction conditions for the synthesis of 2*H*-indazoles would be still desirable.

During the process by using the post-Ugi cascade reaction for the construction of heterocycles in drug discovery campaign^28,29^, a new methodology in our group was developed to synthesize two types of the 2*H*-indazole scaffolds^30–32^. The methodology features with mild reaction, simple operation procedure, a broad scope of starting materials, and the absence of a catalyst. The 2*H*-indazoles were screened in two cancer cell lines HCT116 and SW620. Compound **7h** which was synthesized and purified in a gram scale in 7 hours, is only 10 times less active than the legendary cancer drug paclitaxel. Herein we disclosed detailed synthesis and biological testing.

## Results and Discussion

### Chemistry

**Scheme 1:**
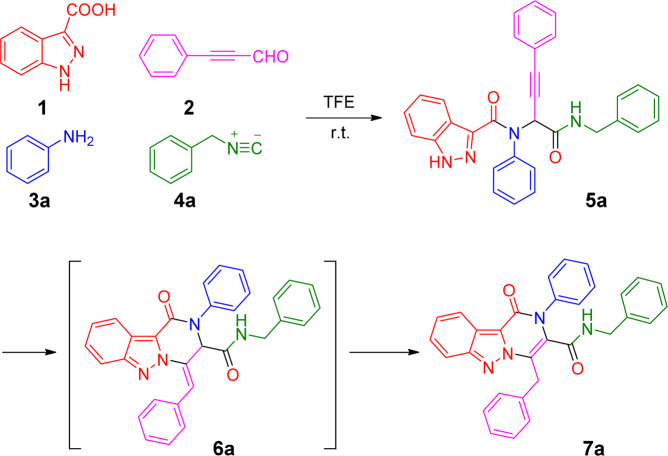
Synthetic routes for compound **7a**.

Using an optimized solvent 2,2,2-trifluoroethanol (TFE) shown in Scheme 1, the reaction of 1*H*-indazole-3-carboxylic acid (**1**) with phenylpropiolaldehyde (**2**), aniline (**3a**), and phenyl isocyanide (**4a**) in TFE at room temperature afforded the Ugi product **5a** in high yield as expected.

With a large amount of compound **5a** in hand, we then explored conditions for the post-Ugi cyclization reaction. In order to find environmental-friendly conditions, we investigated the cyclization reaction by testing inorganic or organic base as a catalyst instead of a metal catalyst (Table 1). Strikingly the Ugi product **5a** was converted to compound **7a** with inorganic base K_2_CO_3_ (2.0 equiv.) in 63% yield. Compound **6a**, the tautomeric precursor of compound **7a** was also isolated with 25% yield and the structure of **6a** was proved by NMR spectra (see supporting information). Further, the desired product **7a** was isolated in 78% yield, and the side product **6a** was reduced to 7% yield by elevating the microwave irradiation temperature from 80 °C to 90 °C. Enhanced temperature to 110 °C led to 89% yield for compound **7a**. Interestingly, 1,8-Diazabicyclo[5.4.0]undec-7-ene (DBU) and diisopropanolamine (DIPA), organic bases, could still afford compound **7a** (entries 4–7) with excellent yields (71–87%). With these exciting results, an investigation without a base or acid was processed. Unprecedentedly, the desired product **7a** was obtained just by microwave heating in DMF for 10 min (entries 8–10). The microwave heating at 110 °C for 20 min in DMF could afford the desired product **7a** in 95% yield. A variety of different solvents were then tested (entries 11–17). For protic solvents (TFE and EtOH), compound **6a** could be isolated together with the product **7a** (entries 11–13, 16–17) with lower yields. Notably, switching the solvent DMF to MeCN or tetrahydrofuran (THF), we failed to isolate either the product **7a** or the intermediate **6a** (entries 14, 15). Therefore, the optimized reaction condition is entry 10: the reaction was heated with microwave irradiation at 110 °C for 20 min in DMF in the absence of a catalyst.

**Table 1:** Optimization of the reaction conditions for the synthesis of compound **7a**.

entry	cat	solv	conditions	yield (%) 6a^a^	yield (%) 7a^a^
1	K_2_CO_3_	DMF	MW, 80 °C, 20 min	25	63
2	K_2_CO_3_	DMF	MW, 90 °C, 10 min	7	78
3	K_2_CO_3_	DMF	MW, 110 °C, 10 min	—	89
4	DBU	DMF	MW, 90 °C, 10 min	—	76
5	DBU	DMF	MW, 110 °C, 10 min	—	87
6	DIPA	DMF	MW, 90 °C, 10 min	—	71
7	DIPA	DMF	MW, 110 °C, 10 min	—	83
8	—	DMF	MW, 90 °C, 10 min	7	77
9	—	DMF	MW, 110 °C, 10 min	—	85
**10**	—	**DMF**	**MW, 110 °C, 20 min**	—	**95**
11	—	TFE	MW, 90 °C, 10 min	12	23
12	—	TFE	MW, 110 °C, 10 min	30	40
13	—	TFE	MW, 110 °C, 20 min	32	51
14	—	MeCN	MW, 90 °C, 10 min	—	—
15	—	THF	MW, 90 °C, 10 min	—	—
16	—	EtOH	MW, 90 °C, 10 min	—	12
17	—	EtOH	MW, 110 °C, 10 min	10	15

^a^Yield (%) based on the integral of the HPLC peaks detected at 254 nm. MW = microwave irradiation. Reaction conditions: **5a** (0.1 mmol), additive (2.0 eq.) in DMF (1.0 mL) under microwave irradiation.

**Scheme 2:**
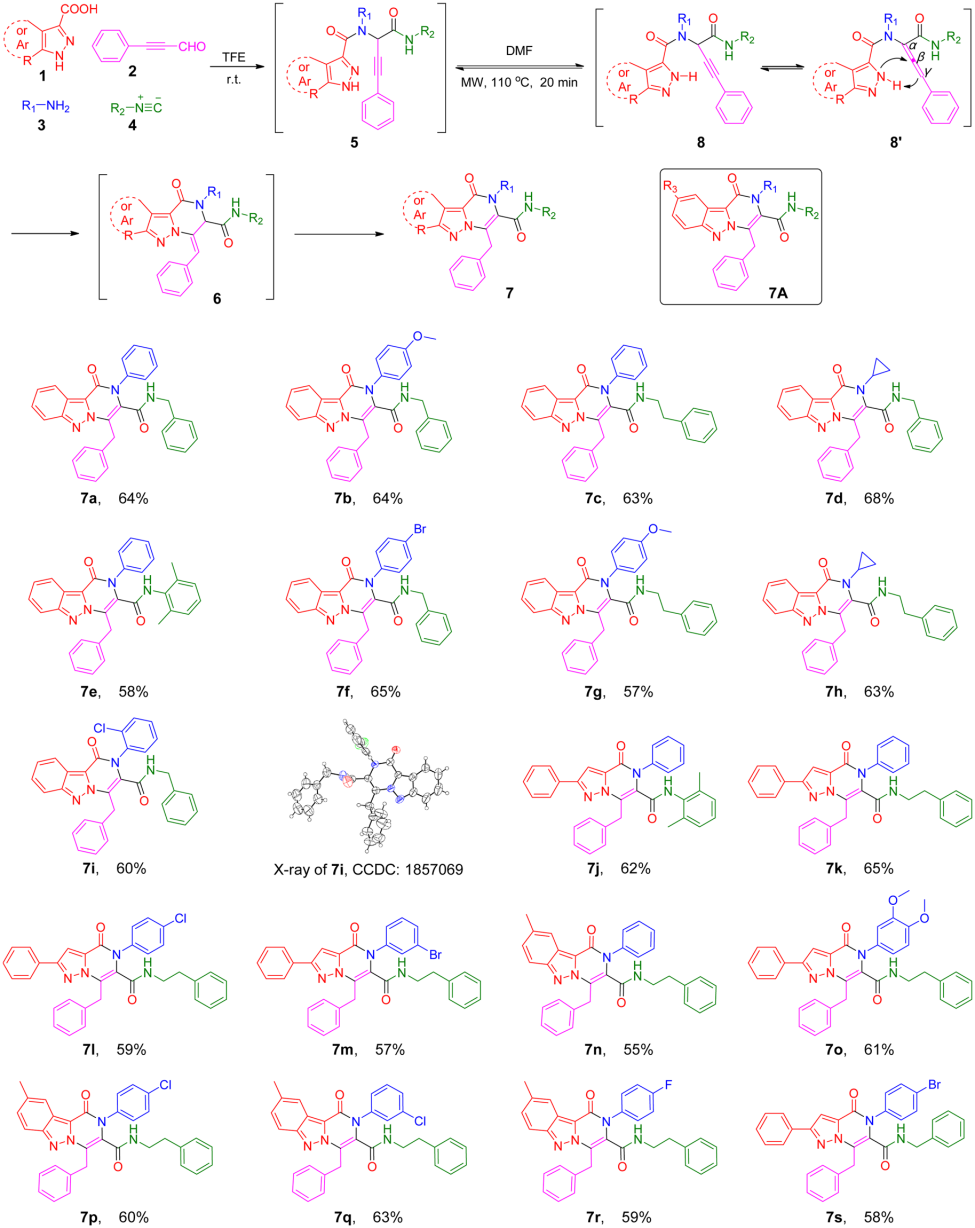
Scope of the Ugi cascade reaction route leading to fused pyrazole-pyrazines **7a-s**. Reaction conditions: **1** (1.0 mmol), **2** (1.0 mmol), **3** (1.0 mmol) and **4** (1.0 mmol) in TFE (2.0 mL) under air; After the removal of solvent, diluted by DMF (2.0 mL), under microwave irradiation at 110 °C, for 20 min. Overall yield of isolated product. See general procedure for synthesizing **7** in SI.

Encouraged by good yields for both the Ugi and cyclization reactions, we investigated the cyclization reaction with the unpurified Ugi product **5a**. In one pot, the cascade reaction produced the final product **7a** in 64% yield for two steps. We then investigated the scope of this one-pot procedure by varying starting materials (in Scheme 2). First, either electron withdrawing or donating phenyl amines and aromatic or aliphatic substituted isocyanides were well tolerated. Strikingly, compounds **7d** and **7h** with small cyclopropanamine were also obtained with good yields. Phenyl pyrazoles (**7j-m**, **7o** and **7s**) or methyl indazoles (**7n**, **7p-r**) didn’t make a significant difference for the reaction yield. It is worth to emphasize that the Ugi adduct **5** did not require purification by column chromatography, with the crude product having no discernible impact on the overall yield. In all cases, the initial Ugi products **5a-s** were used directly in the next step without further purification after the removal of solvent removal. A variety of different starting materials were successfully employed under the optimized conditions for the construction of structurally diverse pyrazole-pyrazines **7a-s** with yields in the range of 55–68% shown in Scheme 2, indicating a good functional group tolerance. The structure of compound **7i** was unequivocally confirmed by X-ray crystallography, as shown in Scheme 2 as well.

Mechanistically, under microwave irradiation the tautomer **8** could be formed. Microwave irradiation would promote an isomerization at the triple bond to generate the intermediate allene **8’**, which in turn would react with the pyrazole to afford the unstable compound **6**. It was surprising to find that the intramolecular *N*2-arylation occurred without a catalyst, which is obviously different from the previous reports^18–24,30,31^. This two-step operation provided the facile access to a variety of complex fused pyrazole-pyrazines **7** in good yields, making it suitable for the construction of small compound libraries and scale-up synthesis.

**Scheme 3:**
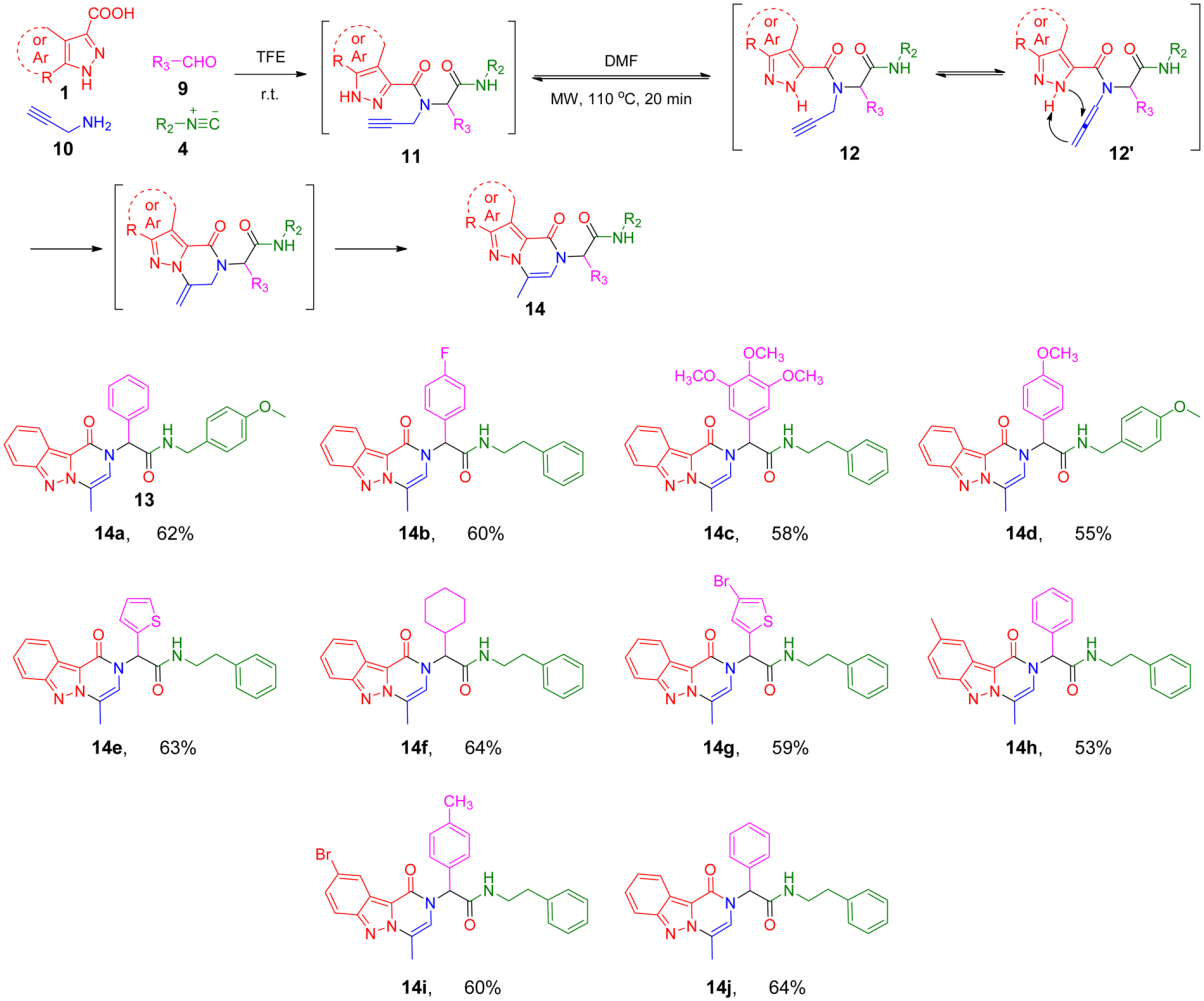
Scope of the Ugi cascade reaction route leading to fused pyrazole-pyrazines **14a-j**. Reaction conditions: **1** (1.0 mmol), **9** (1.0 mmol), **10** (1.0 mmol) and **4** (1.0 mmol) in TFE (2.0 mL) under air; After the removal of solvent, diluted by DMF (2.0 mL), under microwave irradiation at 110 °C, for 20 min. Overall yield of isolated product. See general procedure for synthesizing **14** in SI.

To expand this catalyst-free cyclization for the synthesis of other scaffolds, we investigated if the triple bond would still react with a 2*H*-indazole when it was present in different Ugi inputs. We replaced aniline with 2-propynylamine **10** to provide a triple bond in the amino-Ugi input as depicted in Scheme 3. Similarly to the synthesis of compound **7**, the U-4CR with dazole carboxylic acid **1**, 2-propynylamine **10**, aldehyde **9** and isocyanide **4** progressed smoothly (Scheme 3). The U-4CR was completed at room temperature overnight and evaporated to dryness to give the crude product **11**, which was then subjected to the optimal conditions with the microwave irradiation at 110 °C for 20 min in DMF without a catalyst to give compound **14**. With this condition, we proceeded to investigate the synthesis scope of this new series of pyrazine-pyrazines **14**. In all cases, initial Ugi products **11** were subjected to the cyclization reaction following the solvent removal. A variety of different starting materials were successfully employed in one-pot for the construction of structurally diverse pyrazole-pyrazines **14a-j** with yields of 53–64%.

With a similar mechanism to the preparation of compound **7**, the tautomerization of the Ugi adduct **11** provides intermediate **12** and allenamide **12’** under the microwave irradiation. The nucleophic attach of the allene bond from the pyrazole nitrogen would sequentially afford the unstable intermediate **13**. The double bond isomerization would then give the stable structure **14**. Remarkably, two types of structurally distinct and complex final products **7** or **14** can be synthesized without a catalyst in one-pot, just by varying a triple bond in different Ugi inputs.

### Biology and structure activity relationship (SAR)

To evaluate the potential for developing a drug lead from compounds **7a-s** and **14a-j**, the MTT assay was used to measure cancer cell viability upon the drug treatment (Table 2)^33^. Compounds **7a** and **7c** with an unsubstituted phenyl and compound **7i** with 2-chloro-substituted phenyl at the R_1_ position of **7A** (Scheme 2) were completely inactive, while compounds **7b**, **7f** and **7g** with either an electron donating (-OMe) or withdrawing group (-Br) at the 4-position of the phenyl group were potent with IC_50_ less than 5 µM in both cancer cells, indicating the importance of substituents at the 4-position of the phenyl group. The critical role of a 4-substituted phenyl group at the R_1_ position was further confirmed with derivatives **7j, 7k, 7l, 7m, 7o**, and **7s**, which possess a different pyrazolopyridone core structure. Compounds **7l** and **7o** with a 4-substituted phenyl group demonstrated a good activity; in contrast, other compounds including compound **7m** with a 3-substituted phenyl group was not active at 20 µM. Surprisingly, four compounds (compounds **7n** and **7p-7r**) with a methyl substituted indazole at the R_3_ position of **7A** (Scheme 2) were all inactive against HCT116 and SW620 cell lines at 20 µM. Furthermore, replacement of the non-substituted phenyl (R_1_ of **7A**) group of compound **7a** with a simple cyclopropyl group (**7d**) increased the activity significantly. Especially when the R_2_ group (**7A**, Scheme 2) was switched from the phenylmethyl group (**7a**) to a longer phenylethyl group, compound (**7h**) is equally potent for both cell lines with IC_50_ of 1.3 µM and 1.8 µM against HCT116 and SW620 cell lines, respectively. Finally, all fused pyrazole-pyrazines **14a-j** (Scheme 3) were not active at 20 µM.

**Table 2:** Anticancer activities of compounds **7** and **14**^a^.

compound	HCT116 (µM)	SW620 (µM)
**7a**	>20	>20
**7b**	2.1 ± 0.2	1.4 ± 0.2
**7c**	>20	18.7 ± 6.0
**7d**	1.2 ± 0.1	5.3 ± 1.2
**7e**	>20	>20
**7 f**	2.7 ± 0.7	3.8 ± 0.6
**7 g**	1.8 ± 0.2	1.6 ± 0.3
**7 h**	1.3 ± 0.4	1.8 ± 0.3
**7i**	>20	>20
**7j**	>20	>20
**7k**	17.2 ± 5.1	14.5 ± 3.7
**7 l**	2.9 ± 0.8	1.4 ± 0.5
**7 m**	>20	>20
**7n**	>20	>20
**7o**	3.4 ± 1.0	2.1 ± 0.9
**7p**	>20	>20
**7q**	>20	>20
**7r**	>20	>20
**7 s**	8.5 ± 1.0	11.3 ± 3.3
**14a**	>20	>20
**14b**	>20	>20
**14c**	>20	>20
**14d**	>20	>20
**14e**	>20	>20
**14 f**	>20	>20
**14 g**	>20	>20
**14 h**	>20	>20
**14i**	>20	>20
**14j**	>20	>20
Paclitaxel	0.14 ± 0.05	-

^a^All MTT assays were repeated three times by using six samples per assay.

**Figure 2:**
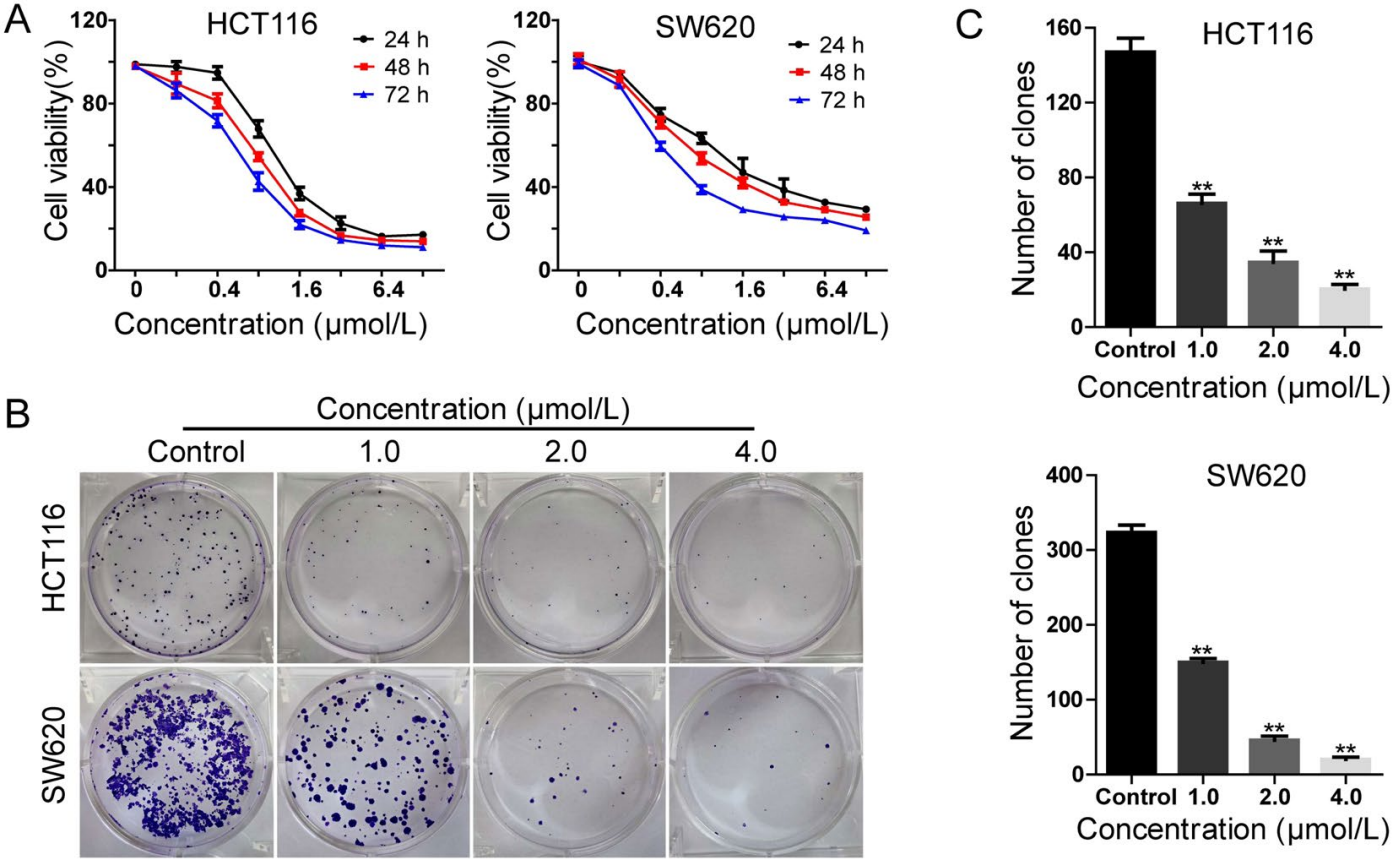
Anticancer activities of compound **7h** against human colon cancer cells. (A) HCT116 and SW620 cells were treated with the different concentrations of compound **7h** for 24, 48 and 72 h. Cell viability was measured with the MTT assay. Data are the mean ± SD of three independent experiments, and each experiment was conducted in sextuplicate. (B, C) The soft agar assay was employed to detect colony formation *in vitro* after treating with the indicated concentrations of compound **7h** for 14 days. The colonies were visualized with the images (B) and quantitated by histogram (C). All data were demonstrated as the mean ± SD of three independent experiments. *P < 0.05; **P < 0.01; ***P < 0.001 versus vehicle.

As shown in Fig. 2A, the compound **7h** efficiently inhibited colon cancer cell viability in a time- and dose-dependent manner. Moreover, soft agar assay *in vitro* was performed to evaluate the effect of the compound **7h** in colony formation, and the results demonstrated that smaller and lesser colonies were formed in treated groups (1, 2 and 4 μmol/L) compared with control groups in both glioblastoma cell lines (Fig. 2B,C). These results supported that the compound **7h** dramatically inhibited cell viability and proliferation in human colon cells.

**Figure 3:**
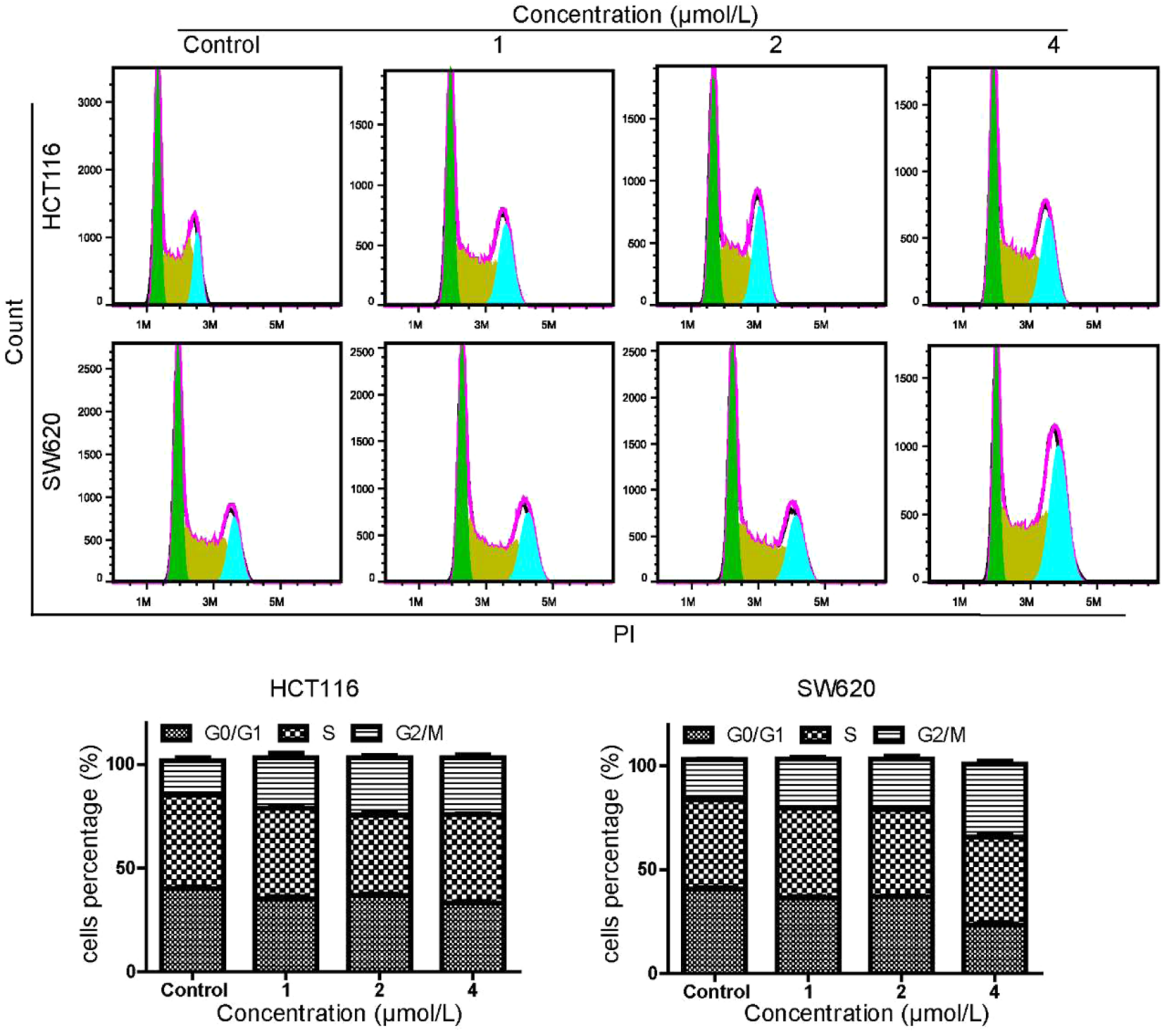
Compound **7h** induces cell cycle arrest at G2/M phase. Cell cycle of HCT116 and SW620 was analyzed by flow cytometry following treatment with compound **7h** for 48 h. Graph is a representative of percentage mean of three independent experiments. Histograms represent the percentages of cell distribution in G0/G1-, S- and G2/M-phase.

To explore the underlying mechanism responsible to compound **7h**-induced cell proliferation, cell cycle distribution was analyzed using flow cytometry in colon cancer cells treated with or without the inhibitor. Control and treated cells were harvested and stained with PI and then measured by flow cytometry. As shown in Fig. 3, compound **7h** affected the cell cycle progression in a dose-dependent manner in HCT116 and SW620 cell lines. Representative histograms further showed that the percentage of G2/M-phase cells increased after exposing to different concentrations of compound **7h**, implying that this compound induced cell cycle arrest at the G2/M phase to inhibit cell proliferation.

## Conclusions

In summary, two series of densely substituted and distinct fused pyrazole-pyrazines were synthesized by using an Ugi reaction following an intramolecular *N*2-arylation of pyrazoles with allenes at the C-β position in absence of a catalyst in one-pot. Under the mild reaction condition, simple operation procedure, commercial availability of starting materials, and good overall yield, the novel methodology will offer a quick access to diverse indazole and pyrazole analogues for biological testing in a cost-effective and environmental-friendly manner. The gram scale synthesis of compound **7** can be accomplished within in one day (see SI for details). To our best knowledge, this is the first report for post-Ugi cascade reaction to prepare pyrazole-pyrazine scaffolds without a catalyst in one-pot. This catalyst-free cascade methodology would be highly applicable to other MCRs such as Passerini^34^, Petasis^35^, Betti^36^, Kabachnik-Fields^37^, and GBBR^38^.

The preliminary screening results of compounds **7** in the colon cancer cell lines HCT116 and SW620 are promising. Considering that compounds **7b** and **7h** are only 10 times less active than the most popular anticancer drug paclitaxel, **7b** and **7h** provide starting points for the further optimization in the drug discovery value chain for the treatment of colorectal cancer. Further efforts are ongoing for applicable design strategy of this cascade cyclization to synthesize new scaffolds and optimize **7h** toward a clinical candidate.

## Experimental Section

### General information

^1^H and ^13^C NMR were recorded on a Bruker 400 spectrometer. ^1^H NMR data are reported as follows: chemical shift in ppm (δ), multiplicity (s = singlet, d = doublet, t = triplet, m = multiplet), coupling constant (Hz), relative intensity. ^13^C NMR data are reported as follows: chemical shift in ppm (δ). HPLC-MS analyses were performed on a Shimadzu-2020 LC-MS instrument using the following conditions: Shim-pack VPODS C18 column (reverse phase, 150 × 2.0 mm); 80% acetonitrile and 20% water over 6.0 min; flow rate of 0.4 mL/min; UV photodiode array detection from 200 to 300 nm. The reaction was irradiated at the required ceiling temperature (the reaction temperature was monitored by an external surface sensor using the Biotage Initiator reactor) using maximum power for the stipulated time. Then it was cooled to 50 °C with gas jet cooling. The products were purified by Biotage Isolera™ Spektra Systems and Hexane/EtOAc solvent systems. All reagents and solvents were obtained from commercial sources and used without further purification.

### General procedure for compound **7**

A solution of 3-phenylpropiolaldehyde (1.0 mmol) and amine (1.0 mmol) in CF_3_CH_2_OH (TFE) (2 mL) was stirred at room temperature for 10 min in a 10 mL microwave vial. Then, carboxylic acid (1.0 mmol) and isocyanide (1.0 mmol) were added separately. After completion of the reaction (monitored by TLC), the solvent was removed under a nitrogen stream (The Ugi product **5a** and **5b** were purified). The residue was dissolved in DMF (2.0 mL), sealed and heated in microwave at 110 °C for 20 min. After the microwave vial was cooled to room temperature, the solvent was removed under reduced pressure and then diluted with EtOAc (15 mL) and washed with brine. The organic layer was dried over Na_2_SO_4_ and concentrated. The residue was purified by silica gel column chromatography using a gradient of ethyl acetate/hexane (0–60%) to afford the relative pyrazoles products **7a-s**. (The crude Ugi compound **5a** was heated in microwave at 80 °C for 20 min to give products **6a** (22%) and **7a** (60%)).

*N-(1-(benzylamino)-1-oxo-4-phenylbut-3-yn-2-yl)-N-phenyl-1H-indazole-3-carboxamide* (**5a**), 420 mg light yellow solid, yield 87%, HPLC purity >96%, mp 117–119 °C. ^1^H NMR (400 MHz, CDCl_3_) δ 8.27 (d, J = 8.3 Hz, 1 H), 7.89 (d, J = 8.8 Hz, 1 H), 7.61–7.40 (m, 5 H), 7.35 (d, J = 7.1 Hz, 4 H), 7.31–7.05 (m, 7 H), 6.77 (d, J = 7.2 Hz, 1 H), 6.04 (s, 1 H), 4.54 (s, 1 H), 4.13 (d, J = 5.5 Hz, 2 H). ^13^C NMR (100 MHz, CDCl_3_) δ 160.63, 154.76, 148.85, 137.41, 136.32, 136.16, 129.34, 129.23, 128.69, 128.61, 128.50, 128.29, 127.94, 127.78, 126.89, 124.82, 124.44, 121.34, 120.98, 117.72, 82,57, 81.38, 58.45, 44.44. HRMS (ESI) m/z calcd for C_31_H_25_N_4_O_2_^+^ (M + H)^+^ 485.1972, found 485.1973.

*N-(1-(benzylamino)-1-oxo-4-phenylbut-3-yn-2-yl)-N-(4-methoxyphenyl)-1H-indazole-3-carboxamide* (**5b**), 465 mg white solid, yield 90%, HPLC purity >94%, mp 118–120 °C. ^1^H NMR (400 MHz, DMSO-*d*_6_) δ 12.20 (s, 1 H), 8.41–7.83 (m, 1 H), 7.78 (d, *J* = 7.7 Hz, 2 H), 7.74 (d, *J* = 8.1 Hz, 1 H), 7.65–7.47 (m, 2 H), 7.45 (d, *J* = 7.7 Hz, 2 H), 7.31 (t, *J* = 7.4 Hz, 1 H), 7.24 (d, *J* = 7.3 Hz, 1 H), 7.22–7.06 (m, 3 H), 6.86 (t, *J* = 7.1 Hz, 1 H), 6.76 (d, *J* = 8.5 Hz, 2 H), 5.49 (s, 1 H), 4.54 (d, *J* = 5.8 Hz, 2 H), 3.65 (s, 3 H). ^13^C NMR (100 MHz, DMSO-*d*_6_) δ 158.67, 132.54, 132.30, 130.92, 130.29, 129.59, 129.32, 129.15, 127.50, 127.33, 126.11, 125.71, 124.49, 119.15, 116.04, 115.11, 114.98, 113.97, 79.65, 55.41, 48.78. HRMS (ESI) m/z calcd for C_32_H_27_N_4_O_3_^+^ (M + H)^+^ 515.2078, found 515.2075.

*N-benzyl-4-benzylidene-1-oxo-2-phenyl-1,2,3,4-tetrahydropyrazino[1,2-b]indazole-3-carboxamide* (**6a**), 106 mg white solid, yield 22%, HPLC purity >98%, mp 120–122 °C. ^1^H NMR (400 MHz, CDCl_3_) δ 8.21 (d, *J* = 8.5 Hz, 1 H), 7.93 (s, 1 H), 7.83 (d, *J* = 8.8 Hz, 1 H), 7.48 (dd, *J* = 13.4, 7.1 Hz, 3 H), 7.44–7.41 (m, 4 H), 7.38 (t, *J* = 7.2 Hz, 3 H), 7.33 (dd, *J* = 9.3, 6.0 Hz, 2 H), 7.24–7.13 (m, 3 H), 6.92 (d, *J* = 7.0 Hz, 2 H), 5.83 (s, 1 H), 5.74 (s, 1 H), 4.31 (m, 2 H). ^13^C NMR (100 MHz, CDCl_3_) δ 166.70, 156.95, 149.63, 139.98, 136.72, 133.07, 130.19, 129.54, 129.03, 128.72, 128.31, 127.80, 127.65, 127.43, 126.26, 125.70, 124.67, 123.29, 122.87, 121.70, 118.08, 62.75, 44.24. HRMS (ESI) m/z calcd for C_31_H_25_N_4_O_2_^+^ (M + H)^+^ 485.1972, found 485.1972.

*N,4-dibenzyl-1-oxo-2-phenyl-1,2-dihydropyrazino[1,2-b]indazole-3-carboxamide* (**7a**), 310 mg white solid, yield 64%, HPLC purity >98%, mp 124–126^o^C. ^1^H NMR (400 MHz, CDCl_3_) δ 8.32 (d, *J* = 8.3 Hz, 1 H), 7.92 (d, *J* = 8.8 Hz, 1 H), 7.52 (s, 1 H), 7.47 (s, 3 H), 7.43–7.31 (m, 5 H), 7.25 (s, 2 H), 7.23 (s, 2 H), 7.20 (s, 2 H), 6.78 (d, *J* = 5.7 Hz, 2 H), 5.82 (s, 1 H), 4.60 (s, 2 H), 4.17 (d, *J* = 5.3 Hz, 2 H). ^13^C NMR (100 MHz, CDCl_3_) δ 160.67, 154.79, 148.91, 137.49, 136.37, 136.16, 129.40, 129.28, 128.76, 128.51, 128.30, 127.99, 127.85, 126.95, 124.89, 124.50, 121.40, 121.04, 117.76, 44.11, 32.36. HRMS (ESI) m/z calcd for C_31_H_25_N_4_O_2_^+^ (M + H)^+^ 485.1972, found 485.1973.

*N,4-dibenzyl-2-(4-methoxyphenyl)-1-oxo-1,2-dihydropyrazino[1,2-b]indazole-3-Carboxamide* (**7b**), 330 mg white solid, yield 64%, HPLC purity >98%, mp 120–122 ^o^C. ^1^H NMR (400 MHz, CDCl_3_) δ 8.32 (d, *J* = 8.3 Hz, 1 H), 7.92 (d, *J* = 8.7 Hz, 1 H), 7.52 (t, *J* = 7.3 Hz, 1 H), 7.41–7.35 (m, 3 H), 7.31 (s, 1 H), 7.28 (s, 1 H), 7.27 (s, 1 H), 7.26–7.25 (m, 1 H), 7.21 (dd, *J* = 12.9, 5.6 Hz, 4 H), 6.95 (d, *J* = 8.9 Hz, 2 H), 6.81 (d, *J* = 6.6 Hz, 2 H), 5.85 (t, *J* = 5.7 Hz, 1 H), 4.59 (s, 2 H), 4.22 (d, *J* = 5.8 Hz, 2 H), 3.87 (s, 3 H). ^13^C NMR (100 MHz, CDCl_3_) δ 160.79, 160.01, 148.94, 137.62, 136.26, 129.76, 128.76, 128.48, 127.92, 126.93, 124.79, 121.14, 120.78, 117.71, 114.62, 55.50, 44.09, 32.37. HRMS (ESI) m/z calcd for C_32_H_27_N_4_O_3_^+^ (M + H)^+^ 515.2078, found 515.2077.

*4-benzyl-1-oxo-N-phenethyl-2-phenyl-1,2-dihydropyrazino[1,2-b]indazole-3-Carboxamide* (**7c**), 313 mg white solid, yield 63%, HPLC purity >98%, mp 122–125 ^o^C. ^1^H NMR (400 MHz, CDCl_3_) δ 8.31 (d, *J* = 8.2 Hz, 1 H), 7.91 (d, *J* = 8.7 Hz, 1 H), 7.51 (dd, *J* = 15.8, 7.4 Hz, 2 H), 7.46 (s, 2 H), 7.39 (d, *J* = 7.7 Hz, 3 H), 7.34 (d, *J* = 7.8 Hz, 2 H), 7.28 (d, *J* = 6.4 Hz, 2 H), 7.25–7.25 (m, 1 H), 7.24–7.18 (m, 3 H), 6.96 (d, *J* = 7.4 Hz, 2 H), 5.55 (s, 1 H), 4.53 (s, 2 H), 3.26 (dd, *J* = 13.0, 6.4 Hz, 2 H), 2.37 (t, *J* = 7.1 Hz, 2 H). ^13^C NMR (100 MHz, CDCl_3_) δ 160.88, 154.77, 148.97, 137.67, 136.57, 129.29, 128.81, 128.67, 128.56, 128.42, 127.98, 126.90, 124.87, 121.07, 117.77, 40.84, 34.56, 32.40. HRMS (ESI) m/z calcd for C_32_H_27_N_4_O_2_^+^ (M + H)^+^ 499.2129, found 499.2129.

*N,4-dibenzyl-2-cyclopropyl-1-oxo-1,2-dihydropyrazino[1,2-b]indazole-3-Carboxamide* (**7d**), 305 mg white solid, yield 68%, HPLC purity >98%, mp 119–122 ^o^C. ^1^H NMR (400 MHz, CDCl_3_) δ 8.21 (d, *J* = 8.4 Hz, 1 H), 7.81 (d, *J* = 8.7 Hz, 1 H), 7.48 (dd, *J* = 11.2, 4.3 Hz, 1 H), 7.36–7.33 (m, 3 H), 7.31 (d, *J* = 8.1 Hz, 3 H), 7.24 (s, 2 H), 7.19 (d, *J* = 7.4 Hz, 3 H), 6.81 (s, 1 H), 4.62 (d, *J* = 5.8 Hz, 2 H), 4.28 (s, 2 H), 3.20–3.08 (m, 1 H), 1.02 (d, *J* = 6.9 Hz, 2 H), 0.94 (s, 2 H). ^13^C NMR (100 MHz, CDCl_3_) δ 161.10, 148.73, 137.47, 136.76, 129.37, 128.95, 128.63, 128.53, 128.38, 128.13, 127.78, 126.82, 124.61, 121.26, 120.88, 117.74, 44.47, 32.28, 29.52, 8.87. HRMS (ESI) m/z calcd for C_28_H_25_N_4_O_3_^+^ (M + H)^+^ 449.1972, found 449.1977.

*4-benzyl-N-(2,6-dimethylphenyl)-1-oxo-2-phenyl-1,2-dihydropyrazino[1,2-b]indazole-3-carboxamide* (**7e**), 290 mg white solid, yield 58%, HPLC purity >98%, mp 125–127 ^o^C. ^1^H NMR (400 MHz, CDCl_3_) δ 8.30 (d, *J* = 8.4 Hz, 1 H), 7.89 (d, *J* = 8.7 Hz, 1 H), 7.55 (d, *J* = 7.7 Hz, 2 H), 7.51 (d, *J* = 7.9 Hz, 2 H), 7.48 (d, *J* = 6.4 Hz, 1 H), 7.44 (d, *J* = 7.5 Hz, 2 H), 7.40–7.34 (m, 1 H), 7.28 (d, *J* = 7.3 Hz, 2 H), 7.20 (t, *J* = 7.3 Hz, 1 H), 7.06 (dd, *J* = 16.5, 9.0 Hz, 2 H), 6.94 (d, *J* = 7.5 Hz, 2 H), 4.76 (s, 2 H), 1.71 (s, 6 H). ^13^C NMR (100 MHz, CDCl_3_) δ 158.93, 155.15, 149.15, 136.81, 134.76, 132.11, 129.74, 129.60, 129.48, 128.68, 128.48, 128.05, 127.87, 126.94, 125.04, 121.40, 121.05, 117.92, 32.88, 18.21. HRMS (ESI) m/z calcd for C_32_H_27_N_4_O_2_^+^ (M + H)^+^ 499.2129, found 499.2126.

*N,4-dibenzyl-2-(4-bromophenyl)-1-oxo-1,2-dihydropyrazino[1,2-b]indazole-3-carboxamide* (**7f**), 365 mg white solid, yield 65%, HPLC purity >98%, mp 171–173 ^o^C. ^1^H NMR (400 MHz, CDCl_3_) δ 8.30 (d, *J* = 8.3 Hz, 1 H), 7.93 (d, *J* = 8.7 Hz, 1 H), 7.54 (dd, *J* = 11.6, 8.7 Hz, 3 H), 7.43–7.37 (m, 1 H), 7.35 (d, *J* = 7.1 Hz, 2 H), 7.28 (s, 4 H), 7.24 (t, *J* = 5.3 Hz, 4 H), 6.86–6.75 (m, 2 H), 5.87 (s, 1 H), 4.59 (s, 2 H), 4.23 (d, *J* = 5.9 Hz, 2 H). ^13^C NMR (100 MHz, CDCl_3_) δ 160.46, 137.45, 132.60, 130.30, 128.85, 128.76, 128.43, 128.02, 127.06, 125.10, 121.05, 117.85, 44.14, 32.38. HRMS (ESI) m/z calcd for C_31_H_24_BrN_4_O_2_^+^ (M + H)^+^ 563.1077, found 563.1081 and 565.1061.

*4-benzyl-2-(4-methoxyphenyl)-1-oxo-N-phenethyl-1,2-dihydropyrazino[1,2-b]indazole-3-carboxamide* (**7g**), 300 mg white solid, yield 57%, HPLC purity >98%, mp 124–126 ^o^C. ^1^H NMR (400 MHz, CDCl_3_) δ 8.31 (d, *J* = 8.3 Hz, 1 H), 7.91 (d, *J* = 8.7 Hz, 1 H), 7.56–7.47 (m, 1 H), 7.41–7.35 (m, 3 H), 7.31–7.26 (m, 3 H), 7.25–7.19 (m, 5 H), 6.97 (dd, *J* = 7.6, 5.6 Hz, 4 H), 5.55 (t, *J* = 5.5 Hz, 1 H), 4.51 (s, 2 H), 3.84 (s, 3 H), 3.31 (dd, *J* = 13.1, 7.0 Hz, 2 H), 2.44 (t, *J* = 7.1 Hz, 2 H). ^13^C NMR (100 MHz, CDCl_3_) δ 160.96, 159.97, 155.08, 148.92, 137.75, 137.63, 129.66, 128.82, 128.67, 128.50, 128.41, 127.93, 126.94, 124.77, 121.11, 117.70, 114.55, 55.58, 40.81, 34.62, 32.41. HRMS (ESI) m/z calcd for C_33_H_29_N_4_O_3_^+^ (M + H)^+^ 529.2234, found 529.2236.

*4-benzyl-2-cyclopropyl-1-oxo-N-phenethyl-1,2-dihydropyrazino[1,2-b]indazole-3-carboxamide* (**7h**), 290 mg white solid, yield 63%, HPLC purity >98%, mp 121–123 ^o^C. ^1^H NMR (400 MHz, CDCl_3_) δ 8.23 (d, *J* = 8.4 Hz, 1 H), 7.82 (d, *J* = 8.7 Hz, 1 H), 7.53–7.44 (m, 1 H), 7.37–7.32 (m, 1 H), 7.29 (dd, *J* = 10.2, 3.5 Hz, 4 H), 7.23 (d, *J* = 7.6 Hz, 3 H), 7.19 (t, *J* = 7.5 Hz, 3 H), 6.42 (s, 1 H), 4.23 (s, 2 H), 3.78 (dd, *J* = 12.7, 6.8 Hz, 2 H), 3.11–2.99 (m, 1 H), 2.91 (t, *J* = 6.9 Hz, 2 H), 1.01 (d, *J* = 6.8 Hz, 2 H), 0.88 (s, 2 H). ^13^C NMR (100 MHz, CDCl_3_) δ 161.42, 156.16, 148.73, 138.02, 137.60, 129.50, 128.84, 128.63, 128.41, 127.76, 126.92, 126.86, 124.58, 121.21, 120.86, 120.69, 117.73, 41.13, 34.94, 32.29, 29.49, 8.84. HRMS (ESI) m/z calcd for C_29_H_27_N_4_O_2_^+^ (M + H)^+^ 463.2129, found 463.2131. Gram scale synthesis of compound **7h**. A solution of 3-phenylpropiolaldehyde (5.0 mmol) and cyclopropanamine (5.0 mmol) in TFE (10 mL) was stirred at room temperature for 10 min. Then, 1*H*-indazole-3-carboxylic acid (5.0 mmol) and phenethyl isocyanide (5.0 mmol) were added separately. After completion of the reaction (monitored by TLC), the solvent was removed under reduced pressure. The residue was dissolved in DMF (10.0 mL), sealed and heated in microwave at 110 ^o^C for 20 min. After the microwave vial was cooled to room temperature, the solvent was removed under reduced pressure and then diluted with EtOAc (60 mL). After washed with brine, the organic layer was dried over Na_2_SO_4_ and concentrated. The residue was purified by silica gel column chromatography using a gradient of ethyl acetate/hexane (0–60%) to afford compound **7 h** (1.18 g) with 51% yield in 7 hours.

*N,4-dibenzyl-2-(2-chlorophenyl)-1-oxo-1,2-dihydropyrazino[1,2-b]indazole-3-carboxamide* (**7i**), 310 mg white solid, yield 60%, HPLC purity >98%, mp 166–168 ^o^C. ^1^H NMR (400 MHz, CDCl_3_) δ 8.31 (d, *J* = 8.4 Hz, 1 H), 7.91 (d, *J* = 8.7 Hz, 1 H), 7.51 (dd, *J* = 10.2, 5.3 Hz, 2 H), 7.45–7.36 (m, 4 H), 7.34 (d, *J* = 7.8 Hz, 2 H), 7.29–7.23 (m, 3 H), 7.21 (dd, *J* = 13.0, 5.5 Hz, 3 H), 6.84 (d, *J* = 7.0 Hz, 2 H), 6.16 (s, 1 H), 4.68 (d, *J* = 15.9 Hz, 1 H), 4.44 (d, *J* = 15.9 Hz, 1 H), 4.27 (dd, *J* = 14.5, 6.6 Hz, 1 H), 4.07 (dd, *J* = 14.5, 5.3 Hz, 1 H). ^13^C NMR (100 MHz, CDCl_3_) δ 160.24, 154.12, 148.90, 137.27, 136.18, 134.08, 133.02, 131.55, 131.03, 130.14, 128.77, 128.74, 128.29, 128.11, 128.01, 127.90, 127.81, 126.94, 125.02, 124.48, 121.55, 121.08, 120.71, 117.83, 43.99, 32.46. HRMS (ESI) m/z calcd for C_31_H_24_ClN_4_O_2_^+^ (M + H)^+^ 519.1582, found 519.1586.

*7-benzyl-N-(2,6-dimethylphenyl)-4-oxo-2,5-diphenyl-4,5-dihydropyrazolo[1,5-a]pyrazine-6-carboxamide* (**7j**), 325 mg white solid, yield 62%, HPLC purity >97%, mp 135–137 ^o^C. ^1^H NMR (400 MHz, CDCl_3_) δ 7.85 (d, *J* = 7.1 Hz, 2 H), 7.54 (d, *J* = 7.6 Hz, 2 H), 7.48 (d, *J* = 7.2 Hz, 2 H), 7.45–7.41 (m, 3 H), 7.41–7.34 (m, 3 H), 7.32 (s, 1 H), 7.28 (d, *J* = 7.0 Hz, 2 H), 7.19 (t, *J* = 7.3 Hz, 1 H), 7.07–7.00 (m, 1 H), 6.94 (d, *J* = 7.5 Hz, 2 H), 4.59 (s, 2 H), 1.70 (s, 6 H). ^13^C NMR (100 MHz, CDCl_3_) δ 159.17, 154.89, 153.62, 137.09, 136.93, 134.69, 134.15, 132.35, 131.94, 129.61, 129.35, 129.17, 128.95, 128.82, 128.52, 128.48, 127.70, 126.86, 126.24, 124.94, 122.32, 103.82, 32.81, 18.25. HRMS (ESI) m/z calcd for C_34_H_29_N_4_O_2_^+^ (M + H)^+^ 525.2285, found 525.2299.

*7-benzyl-4-oxo-N-phenethyl-2,5-diphenyl-4,5-dihydropyrazolo[1,5-a]pyrazine-6-carbox amide* (**7k**), 340 mg white solid, yield 65%, HPLC purity >98%, mp 129–131 °C. ^1^H NMR (400 MHz, CDCl_3_) δ 7.88 (d, *J* = 7.2 Hz, 2 H), 7.45 (d, *J* = 7.2 Hz, 3 H), 7.44–7.40 (m, 4 H), 7.39 (s, 1 H), 7.35 (s, 1 H), 7.32–7.26 (m, 4 H), 7.21 (dd, *J* = 14.1, 7.1 Hz, 4 H), 6.98 (d, *J* = 7.0 Hz, 2 H), 5.75 (t, *J* = 5.6 Hz, 1 H), 4.36 (s, 2 H), 3.23 (dd, *J* = 13.4, 7.0 Hz, 2 H), 2.36 (t, *J* = 7.3 Hz, 2 H). ^13^C NMR (100 MHz, CDCl_3_) δ 161.13, 154.49, 153.34, 137.88, 137.60, 136.91, 134.24, 132.03, 129.26, 129.07, 128.95, 128.83, 128.76, 128.53, 128.45, 128.44, 126.89, 126.78, 126.24, 124.82, 121.61, 103.73, 40.81, 34.58, 32.35. HRMS (ESI) m/z calcd for C_34_H_29_N_4_O_2_^+^ (M + H)^+^ 525.2285, found 525.2285.

*7-benzyl-5-(4-chlorophenyl)-4-oxo-N-phenethyl-2-phenyl-4,5-dihydropyrazolo[1,5-a] pyrazine-6-carboxamide* (**7l**), 330 mg white solid, yield 59%, HPLC purity >98%, mp 155–157 °C. ^1^H NMR (400 MHz, CDCl_3_) δ 7.89 (d, *J* = 7.2 Hz, 2 H), 7.44 (dd, *J* = 10.2, 4.2 Hz, 3 H), 7.42–7.35 (m, 5 H), 7.33–7.26 (m, 4 H), 7.24 (s, 1 H), 7.21 (dd, *J* = 11.4, 5.2 Hz, 3 H), 6.99 (d, *J* = 6.9 Hz, 2 H), 5.60 (t, *J* = 5.3 Hz, 1 H), 4.36 (s, 2 H), 3.32 (dd, *J* = 12.9, 6.9 Hz, 2 H), 2.48 (t, *J* = 7.0 Hz, 2 H). ^13^C NMR (100 MHz, CDCl_3_) δ 160.96, 154.34, 153.50, 137.69, 137.53, 135.27, 135.04, 134.11, 131.91, 129.72, 129.46, 128.90, 128.85, 128.78, 128.64, 128.37, 127.00, 126.90, 126.25, 124.47, 121.73, 103.99, 40.75, 34.52, 32.36. HRMS (ESI) m/z calcd for C_34_H_28_ClN_4_O_2_^+^ (M + H)^+^ 559.1895, found 559.1895.

*7-benzyl-5-(3-bromophenyl)-4-oxo-N-phenethyl-2-phenyl-4,5-dihydropyrazolo[1,5-a] pyrazine-6-carboxamide* (**7m**), 343 mg white solid, yield 57%, HPLC purity >97%, mp 206–209 °C. ^1^H NMR (400 MHz, CDCl_3_) δ 7.91 (d, J = 5.9 Hz, 2 H), 7.62–7.42 (m, 4 H), 7.42–7.33 (m, 2 H), 7.29 (t, J = 7.5 Hz, 2 H), 7.22 (t, J = 7.2 Hz, 3 H), 7.16 (d, J = 7.4 Hz, 2 H), 6.81 (d, J = 95.5 Hz, 3 H), 4.68 (s, 2 H), 2.19 (s, 6 H). ^13^C NMR (100 MHz, CDCl_3_) δ 153.73, 137.23, 135.64, 134.49, 131.75, 130.35, 129.70, 129.16, 129.07, 128.89, 128.62, 127.32, 126.98, 126.30, 122.84, 103.65, 31.58, 18.73. HRMS (ESI) m/z calcd for C_34_H_28_BrN_4_O_2_^+^ (M + H)^+^ 603.1390, found 603.1395.

*4-benzyl-9-methyl-1-oxo-N-phenethyl-2-phenyl-1,2-dihydropyrazino[1,2-b]indazole-3-carboxamide* (**7n**), 280 mg white solid, yield 55%, HPLC purity >97%, mp 196–198 °C. ^1^H NMR (400 MHz, CDCl_3_) δ 8.05 (s, 1 H), 7.79 (d, *J* = 8.8 Hz, 1 H), 7.50–7.42 (m, 3 H), 7.38 (d, *J* = 7.2 Hz, 2 H), 7.36–7.30 (m, 3 H), 7.27 (d, *J* = 7.2 Hz, 2 H), 7.25–7.18 (m, 4 H), 6.95 (d, *J* = 6.9 Hz, 2 H), 5.63 (s, 1 H), 4.48 (s, 2 H), 3.24 (d, *J* = 6.4 Hz, 2 H), 2.49 (s, 3 H), 2.35 (t, *J* = 7.3 Hz, 2 H). ^13^C NMR (100 MHz, CDCl_3_) δ 160.97, 154.87, 147.83, 137.76, 137.62, 136.64, 134.88, 130.76, 129.26, 129.13, 128.77, 128.63, 128.59, 128.55, 128.41, 128.07, 126.89, 126.79, 123.74, 121.80, 121.04, 119.38, 117.39, 40.83, 34.55, 32.37, 21.97. HRMS (ESI) m/z calcd for C_33_H_29_N_4_O_2_^+^ (M + H)^+^ 513.2285, found 513.2285.

*7-benzyl-5-(3,4-dimethoxyphenyl)-4-oxo-N-phenethyl-2-phenyl-4,5-dihydropyrazolo[1,5-a] pyrazine-6-carboxamide* (**7o**), 356 mg white solid, yield 61%, HPLC purity >98%, mp 187–190 °C. ^1^H NMR (400 MHz, CDCl_3_) δ 7.90 (d, *J* = 7.3 Hz, 2 H), 7.44 (t, *J* = 6.9 Hz, 4 H), 7.38 (dd, *J* = 8.5, 4.8 Hz, 2 H), 7.28 (d, *J* = 7.2 Hz, 2 H), 7.26–7.15 (m, 4 H), 6.99 (d, *J* = 7.3 Hz, 2 H), 6.83 (dd, *J* = 12.3, 6.9 Hz, 3 H), 5.68 (d, *J* = 4.2 Hz, 1 H), 4.35 (s, 2 H), 3.89 (s, 3 H), 3.83 (s, 3 H), 3.32 (dd, *J* = 13.0, 6.7 Hz, 2 H), 2.47 (t, *J* = 7.0 Hz, 2 H). ^13^C NMR (100 MHz, CDCl_3_) δ 161.26, 154.73, 153.27, 149.52, 149.35, 137.85, 137.62, 134.23, 132.04, 129.49, 128.94, 128.83, 128.78, 128.52, 128.38, 126.88, 126.79, 126.24, 125.16, 121.36, 120.62, 112.04, 110.94, 103.65, 56.18, 56.09, 40.76, 34.75, 32.33. HRMS (ESI) m/z calcd for C_36_H_33_N_4_O_4_^+^ (M + H)^+^ 585.2496, found 585.2504.

*4-benzyl-2-(4-chlorophenyl)-9-methyl-1-oxo-N-phenethyl-1,2-dihydropyrazino[1,2-b] indazole-3-carboxamide* (**7p**), 327 mg white solid, yield 60%, HPLC purity >98%, mp 199–202 °C. ^1^H NMR (400 MHz, CDCl_3_) δ 8.05 (s, 1 H), 7.80 (d, *J* = 8.8 Hz, 1 H), 7.41 (d, *J* = 8.6 Hz, 2 H), 7.35 (d, *J* = 8.7 Hz, 3 H), 7.28 (dd, *J* = 7.7, 3.3 Hz, 3 H), 7.24 (d, *J* = 8.7 Hz, 5 H), 6.96 (d, *J* = 6.8 Hz, 2 H), 5.56 (s, 1 H), 4.50 (s, 2 H), 3.32 (dd, *J* = 12.9, 6.9 Hz, 2 H), 2.50 (s, 3 H), 2.47 (t, *J* = 7.0 Hz, 2 H). ^13^C NMR (100 MHz, CDCl_3_) δ 160.83, 154.73, 147.90, 137.61, 137.57, 135.15, 135.02, 130.91, 129.88, 129.45, 128.87, 128.75, 128.41, 128.36, 127.73, 127.02, 126.92, 123.60, 121.90, 121.19, 119.33, 117.46, 40.77, 34.49, 32.41, 21.99. 327 mg white solid, yield 60%. HRMS (ESI) m/z calcd for C_33_H_28_ClN_4_O_2_^+^ (M + H)^+^ 547.1895, found 547.1896.

*4-benzyl-2-(3-chlorophenyl)-9-methyl-1-oxo-N-phenethyl-1,2-dihydropyrazino[1,2-b] indazole-3-carboxamide* (**7q**), 344 mg white solid, yield 63%, HPLC purity >98%, mp 197–200 °C. ^1^H NMR (400 MHz, CDCl_3_) δ 11.36 (s, 1 H), 7.99 (s, 1 H), 7.78 (d, *J* = 8.8 Hz, 1 H), 7.40 (d, *J* = 7.5 Hz, 2 H), 7.29 (d, *J* = 8.6 Hz, 5 H), 7.26–7.16 (m, 4 H), 6.92 (dd, *J* = 87.9, 57.8 Hz, 4 H), 4.58 (s, 2 H), 4.13 (s, 2 H), 3.04 (s, 2 H), 2.24 (s, 3 H). ^13^C NMR (100 MHz, CDCl_3_) δ 162.33, 156.29, 147.60, 137.81, 137.07, 135.01, 134.92, 130.73, 130.41, 128.90, 128.69, 128.51, 127.13, 126.99, 126.82, 125.39, 123.80, 123.74, 122.55, 121.15, 119.00, 117.47, 52.31, 33.82, 31.39, 21.66. HRMS (ESI) m/z calcd for C_33_H_28_ClN_4_O_2_^+^ (M + H)^+^ 547.1895, found 547.1898.

*4-benzyl-2-(4-fluorophenyl)-9-methyl-1-oxo-N-phenethyl-1,2-dihydropyrazino[1,2-b] indazole-3-carboxamide* (**7r**), 313 mg white solid, yield 59%, HPLC purity >96%, mp 185–187 °C. ^1^H NMR (400 MHz, CDCl_3_) δ 8.03 (s, 1 H), 7.78 (d, *J* = 8.8 Hz, 1 H), 7.35 (t, *J* = 7.4 Hz, 3 H), 7.28 (d, *J* = 7.1 Hz, 4 H), 7.25–7.15 (m, 4 H), 7.10 (t, *J* = 8.4 Hz, 2 H), 6.96 (d, *J* = 6.8 Hz, 2 H), 5.70 (d, *J* = 5.4 Hz, 1 H), 4.44 (s, 2 H), 3.30 (dd, *J* = 13.0, 6.8 Hz, 2 H), 2.49 (s, 3 H), 2.45 (t, *J* = 7.1 Hz, 2 H). ^13^C NMR (100 MHz, CDCl_3_) δ 161.33, 160.87, 154.94, 147.83, 137.66, 137.51, 135.07, 132.35, 130.83, 130.53, 130.45, 128.83, 128.70, 128.48, 138.37, 127.99, 126.98, 126.88, 123.57, 121.78, 120.95, 119.26, 117.46, 116.33, 116.11, 40.81, 34.55, 32.42, 21.98. ^19^F NMR (377 MHz, CDCl_3_) δ -111.28. HRMS (ESI) m/z calcd for C_33_H_28_FN_4_O_2_^+^ (M + H)^+^ 531.2191, found 531.2184.

*N,7-dibenzyl-5-(4-bromophenyl)-4-oxo-2-phenyl-4,5-dihydropyrazolo[1,5-a]pyrazine-6-carboxamide* (**7s**), 340 mg white solid, yield 58%, HPLC purity >97%, mp 205–208 °C. ^1^H NMR (400 MHz, CDCl_3_) δ 10.77 (s, 1 H), 7.89 (d, *J* = 7.3 Hz, 2 H), 7.45 (d, *J* = 7.2 Hz, 2 H), 7.39 (dd, *J* = 17.3, 10.1 Hz, 4 H), 7.29 (d, *J* = 6.2 Hz, 5 H), 7.24–7.19 (m, 3 H), 7.14 (d, *J* = 8.1 Hz, 2 H), 6.57 (s, 2 H), 5.03 (s, 2 H), 4.49 (s, 2 H). ^13^C NMR (100 MHz, CDCl_3_) δ 162.84, 155.43, 153.20, 139.47, 137.23, 135.71, 134.21, 132.59, 131.95, 129.18, 129.11, 128.89, 128.86, 128.63, 128.58, 128.00, 126.94, 126.24, 123.13, 122.00, 119.97, 103.04, 54.05, 31.21. HRMS (ESI) m/z calcd for C_33_H_26_BrN_4_O_2_^+^ (M + H)^+^ 589.1234, found 589.1233.

### General procedure for compound **14**

The same procedure with compound **7**. (The Ugi product **11a** was purified.)

*N-(2-((4-methoxybenzyl)amino)-2-oxo-1-phenylethyl)-N-(prop-2-yn-1-yl)-1H-indazole-3-carboxamide*, (**11a**), 420 mg white solid, yield 93%, HPLC purity >95%, mp 155–157 °C. ^1^H NMR (400 MHz, CDCl_3_) δ 8.24 (s, 1 H), 7.35 (s, 4 H), 7.33 (d, *J* = 15.9 Hz, 3 H), 7.26 (s, 1 H), 7.23 (d, *J* = 8.1 Hz, 1 H), 7.19 (d, *J* = 8.6 Hz, 2 H), 6.77 (d, *J* = 8.5 Hz, 2 H), 4.51 (s, 2 H), 4.49 (s, 1 H), 3.73 (s, 3 H). ^13^C NMR (100 MHz, CDCl_3_) δ 158.99, 140.57, 129.66, 129.18, 128.91, 128.63, 127.20, 123.91, 122.74, 122.5, 114.00, 110.10, 80.16, 71.79, 55.26, 43.38. HRMS (ESI) m/z calcd for C_27_H_25_N_4_O_3_^+^ (M + H)^+^ 452.1848, found 452.1844.

*N-(4-methoxybenzyl)-2-(4-methyl-1-oxopyrazino[1,2-b]indazol-2(1 H)-yl)-2-phenylacet amide* (**14a**), 380 mg white solid, yield 62%, HPLC purity >98%, mp 195–197 °C. ^1^H NMR (400 MHz, CDCl_3_) δ 8.23 (d, *J* = 8.3 Hz, 1 H), 7.93 (d, *J* = 8.7 Hz, 1 H), 7.57–7.49 (m, 1 H), 7.44 (dd, *J* = 8.5, 3.9 Hz, 5 H), 7.40–7.32 (m, 1 H), 7.10 (d, *J* = 8.5 Hz, 2 H), 7.02 (s, 1 H), 6.91 (s, 1 H), 6.83 (d, *J* = 5.1 Hz, 1 H), 6.71 (d, *J* = 8.5 Hz, 2 H), 4.44 (qd, *J* = 14.5, 5.5 Hz, 2 H), 3.73 (s, 3 H), 2.56 (s, 3 H). ^13^C NMR (100 MHz, CDCl_3_) δ 167.68, 159.07, 155.61, 148.62, 134.64, 129.42, 129.29, 129.22, 129.10, 127.54, 124.19, 124.12, 121.03, 120.86, 119.28, 117.79, 117.39, 114.01, 59.13, 55.23, 43.65, 14.52. HRMS (ESI) m/z calcd for C_27_H_25_N_4_O_3_^+^ (M + H)^+^ 453.1921, found 453.1917.

*2-(4-fluorophenyl)-2-(4-methyl-1-oxopyrazino[1,2-b]indazol-2(1 H)-yl)-N-phenethylacet amide* (**14b**), 272 mg white solid, yield 60%, HPLC purity >98%, mp 199–202 °C. ^1^H NMR (400 MHz, CDCl_3_) δ 8.19 (d, *J* = 8.3 Hz, 1 H), 7.94 (d, *J* = 8.7 Hz, 1 H), 7.57–7.49 (m, 1 H), 7.41–7.30 (m, 3 H), 7.14–7.08 (m, 3 H), 7.06 (t, *J* = 6.8 Hz, 4 H), 7.00–6.92 (m, 2 H), 6.89 (s, 1 H), 3.70 (dq, *J* = 13.5, 7.0 Hz, 1 H), 3.57 (td, *J* = 13.0, 6.5 Hz, 1 H), 2.81 (t, *J* = 6.8 Hz, 2 H), 2.56 (s, 3 H). ^13^C NMR (100 MHz, CDCl_3_) δ 167.78, 164.19, 161.71, 155.52, 148.65, 138.18, 130.92, 130.84, 130.52, 130.49, 128.64, 128.48, 127.65, 126.43, 124.32, 124.04, 121.00, 120.66, 119.54, 117.53, 117.43, 116.43, 116.22, 58.34, 40.86, 35.49, 14.51. ^19^F NMR (377 MHz, CDCl_3_) δ -111.72. HRMS (ESI) m/z calcd for C_27_H_24_FN_4_O_2_^+^ (M + H)^+^ 455.1878, found 455.1884.

*2-(4-methyl-1-oxopyrazino[1,2-b]indazol-2(1 H)-yl)-N-phenethyl-2-(3,4,5-trimethoxyphenyl) acetamide* (**14c**), 334 mg white solid, yield 58%, HPLC purity >98%, mp 201–204 °C. ^1^H NMR (400 MHz, CDCl_3_) δ 8.33 (d, *J* = 8.3 Hz, 1 H), 7.95 (d, *J* = 8.7 Hz, 1 H), 7.58–7.50 (m, 1 H), 7.45–7.38 (m, 1 H), 7.19–7.14 (m, 2 H), 7.11 (dd, *J* = 13.8, 7.1 Hz, 3 H), 6.90 (s, 1 H), 6.75 (s, 1 H), 6.68 (s, 2 H), 6.44 (d, *J* = 5.2 Hz, 1 H), 3.87 (s, 3 H), 3.81 (s, 6 H), 3.71–3.52 (m, 2 H), 2.84 (tq, *J* = 13.8, 7.1 Hz, 2 H), 2.59 (s, 3 H). ^13^C NMR (100 MHz, CDCl_3_) δ 167.80, 155.49, 153.87, 148.67, 138.85, 138.19, 129.54, 128.63, 128.57, 127.64, 126.57, 124.30, 124.17, 121.03, 120.82, 119.31, 117.47, 117.43, 106.43, 60.90, 59.39, 56.37, 41.11, 35.54, 14.60. HRMS (ESI) m/z calcd for C_30_H_31_N_4_O_5_^+^ (M + H)^+^ 527.2289, found 527.2291.

*N-(4-methoxybenzyl)-2-(4-methoxyphenyl)-2-(4-methyl-1-oxopyrazino[1,2-b]indazol-2(1 H)-yl)acetamide* (**14d**), 265 mg white solid, yield 55%, HPLC purity >98%, mp 200–203 °C. ^1^H NMR (400 MHz, CDCl_3_) δ 8.13 (dd, *J* = 17.1, 8.3 Hz, 1 H), 7.91 (d, *J* = 8.7 Hz, 1 H), 7.50 (t, *J* = 7.7 Hz, 1 H), 7.41 (d, *J* = 8.7 Hz, 2 H), 7.30 (dd, *J* = 14.7, 7.7 Hz, 1 H), 7.10 (s, 1 H), 7.07–6.99 (m, 3 H), 6.93 (dd, *J* = 9.2, 5.9 Hz, 3 H), 6.61 (dd, *J* = 15.3, 8.3 Hz, 2 H), 4.49–4.35 (m, 2 H), 3.83 (s, 3 H), 3.75–3.66 (m, 3 H), 2.55 (s, 3 H). ^13^C NMR (100 MHz, CDCl_3_) δ 168.03, 160.25, 158.90, 155.64, 148.59, 130.64, 129.28, 129.13, 127.47, 126.44, 124.13, 124.04, 120.94, 120.82, 119.16, 117.82, 117.33, 114.75, 113.85, 58.88, 55.39, 55.17, 43.57, 14.52. HRMS (ESI) m/z calcd for C_28_H_27_N_4_O_4_^+^ (M + H)^+^ 483.2027, found 483.2028.

*2-(4-methyl-1-oxopyrazino[1,2-b]indazol-2(1 H)-yl)-N-phenethyl-2-(thiophen-2-yl)acetamide* (**14e**), 278 mg white solid, yield 63%, HPLC purity >98%, mp 174–177 °C. ^1^H NMR (400 MHz, CDCl_3_) δ 8.28 (d, *J* = 8.3 Hz, 1 H), 7.94 (d, *J* = 8.6 Hz, 1 H), 7.53 (t, *J* = 7.6 Hz, 1 H), 7.39 (dd, *J* = 12.4, 5.7 Hz, 2 H), 7.25 (d, *J* = 3.3 Hz, 1 H), 7.09 (s, 1 H), 7.05 (d, *J* = 6.5 Hz, 5 H), 7.02 (s, 2 H), 6.81 (d, *J* = 5.1 Hz, 1 H), 3.61 (q, *J* = 6.7 Hz, 2 H), 2.89–2.73 (m, 2 H), 2.58 (s, 3 H). ^13^C NMR (100 MHz, CDCl_3_) δ 167.24, 155.13, 148.67, 138.14, 135.70, 129.64, 128.59, 128.41, 128.19, 127.64, 127.20, 126.33, 124.32, 124.07, 121.05, 120.78, 119.70, 117.48, 116.80, 54.56, 40.98, 35.37, 14.55. HRMS (ESI) m/z calcd for C_25_H_23_N_4_O_2_S^+^ (M + H)^+^ 443.1536, found 443.1532.

*2-cyclohexyl-2-(4-methyl-1-oxopyrazino[1,2-b]indazol-2(1 H)-yl)-N-phenethylacetamide* (**14f**), 282 mg white solid, yield 64%, HPLC purity >98%, mp 178–180 °C. ^1^H NMR (400 MHz, CDCl_3_) δ 8.32 (d, *J* = 8.3 Hz, 1 H), 7.96 (d, *J* = 8.6 Hz, 1 H), 7.59–7.51 (m, 1 H), 7.45–7.37 (m, 1 H), 7.23 (s, 1 H), 7.01 (d, *J* = 4.0 Hz, 3 H), 7.00–6.93 (m, 2 H), 6.68 (s, 1 H), 5.25 (d, *J* = 10.8 Hz, 1 H), 3.54 (q, *J* = 6.7 Hz, 2 H), 2.76 (tq, *J* = 14.0, 7.0 Hz, 2 H), 2.67 (s, 3 H), 2.23 (q, *J* = 10.9 Hz, 1 H), 1.78 (t, *J* = 16.2 Hz, 2 H), 1.65 (d, *J* = 11.2 Hz, 2 H), 1.45–1.28 (m, 2 H), 1.25–1.03 (m, 3 H), 1.00–0.84 (m, 1 H). ^13^C NMR (100 MHz, CDCl_3_) δ 168.80, 155.69, 148.62, 138.15, 128.53, 128.29, 127.64, 126.25, 124.31, 124.19, 121.02, 120.80, 119.73, 117.43, 116.12, 40.45, 37.64, 35.45, 29.95, 28.25, 26.06, 25.43, 25.31, 14.56. HRMS (ESI) m/z calcd for C_27_H_31_N_4_O_2_^+^ (M + H)^+^ 443.2442, found 443.2450.

*2-(4-bromothiophen-2-yl)-2-(4-methyl-1-oxopyrazino[1,2-b]indazol-2(1 H)-yl)-N-phenethyl acetamide* (**14g**), 307 mg white solid, yield 59%, HPLC purity >98%, mp 193–196 °C. ^1^H NMR (400 MHz, CDCl_3_) δ 8.22 (d, *J* = 8.0 Hz, 1 H), 8.03–7.89 (m, 1 H), 7.54 (dd, *J* = 10.4, 4.8 Hz, 1 H), 7.40 (dd, *J* = 9.8, 5.0 Hz, 1 H), 7.28 (dd, *J* = 8.2, 3.8 Hz, 1 H), 7.16 (s, 1 H), 7.07 (s, 1 H), 7.03 (d, *J* = 18.3 Hz, 3 H), 7.01–6.74 (m, 4 H), 3.61 (qd, *J* = 13.4, 6.7 Hz, 2 H), 2.90–2.71 (m, 2 H), 2.60 (d, *J* = 2.8 Hz, 3 H). ^13^C NMR (100 MHz, CDCl_3_) δ 166.68, 155.05, 148.69, 137.95, 137.22, 131.94, 128.52, 128.37, 127.76, 126.36, 125.49, 124.55, 123.90, 121.08,120.62, 120.18, 117.61, 116.31, 110.06, 53.79, 40.91, 35.28, 14.54. HRMS (ESI) m/z calcd for C_25_H_22_BrN_4_O_2_S^+^ (M + H)^+^ 521.0641, found 521.0646.

*2-(4,9-dimethyl-1-oxopyrazino[1,2-b]indazol-2(1 H)-yl)-N-phenethyl-2-phenylacetamide* (**14h**), 238 mg white solid, yield 53%, HPLC purity >98%, mp 174–177 °C. ^1^H NMR (400 MHz, CDCl_3_) δ 8.06 (s, 1 H), 7.83 (d, *J* = 8.8 Hz, 1 H), 7.44–7.38 (m, 3 H), 7.36 (d, *J* = 9.6 Hz, 3 H), 7.21–7.11 (m, 3 H), 7.09 (d, *J* = 7.4 Hz, 2 H), 6.88 (d, *J* = 12.2 Hz, 1 H), 6.82 (s, 1 H), 6.51 (d, *J* = 43.6 Hz, 1 H), 3.70 (dq, *J* = 13.5, 6.9 Hz, 1 H), 3.57 (td, *J* = 13.0, 6.7 Hz, 1 H), 2.83 (t, *J* = 7.0 Hz, 2 H), 2.52 (s, 6 H). ^13^C NMR (100 MHz, CDCl_3_) δ 167.92, 155.67, 147.50, 138.28, 134.66, 134.17, 130.31, 129.31, 129.10, 128.97, 128.70, 128.55, 126.49, 123.45, 121.40, 119.31, 117.25, 117.07, 59.23, 40.95, 35.56, 21.94, 14.50. HRMS (ESI) m/z calcd for C_28_H_26_N_4_O_2_^+^ (M + H)^+^ 451.2129, found 451.2126.

*2-(9-bromo-4-methyl-1-oxopyrazino[1,2-b]indazol-2(1 H)-yl)-N-phenethyl-2-(p-tolyl)acet amide* (**14i**), 316 mg white solid, yield 60%, HPLC purity >98%, mp 207–209 °C. ^1^H NMR (400 MHz, CDCl_3_) δ 8.44 (d, *J* = 1.6 Hz, 1 H), 7.79 (d, *J* = 9.1 Hz, 1 H), 7.57 (dd, *J* = 9.1, 1.8 Hz, 1 H), 7.27 (s, 1 H), 7.25 (s, 1 H), 7.20 (d, *J* = 8.3 Hz, 2 H), 7.15 (d, *J* = 7.4 Hz, 2 H), 7.12 (d, *J* = 6.8 Hz, 1 H), 7.11–7.06 (m, 2 H), 6.89 (s, 1 H), 6.83 (s, 1 H), 6.53 (t, *J* = 5.6 Hz, 1 H), 3.69 (td, *J* = 13.5, 7.0 Hz, 1 H), 3.57 (td, *J* = 13.0, 6.7 Hz, 1 H), 2.83 (t, *J* = 7.0 Hz, 2 H), 2.52 (s, 3 H), 2.37 (s, 3 H). ^13^C NMR (100 MHz, CDCl_3_) δ 167.91, 155.29, 147.04, 139.37, 138.27, 131.26, 131.16, 130.09, 128.95, 128.70, 128.53, 126.46, 123.64, 123.17, 122.08, 119.11, 118.97, 118.35, 117.79, 59.30, 40.98, 35.56, 21.21, 14.43. HRMS (ESI) m/z calcd for C_28_H_26_BrN_4_O_2_^+^ (M + H)^+^ 529.1234, found 529.1234.

*2-(4-methyl-1-oxopyrazino[1,2-b]indazol-2(1 H)-yl)-N-phenethyl-2-phenylacetamide* (**14j**), 280 mg white solid, yield 64%, HPLC purity >98%, mp 189–191 °C. ^1^H NMR (400 MHz, CDCl_3_) δ 8.38–8.27 (m, 1 H), 7.94 (d, *J* = 8.6 Hz, 1 H), 7.53 (t, *J* = 7.7 Hz, 1 H), 7.39 (s, 4 H), 7.37 (d, *J* = 3.5 Hz, 2 H), 7.20–7.14 (m, 2 H), 7.11 (dd, *J* = 16.8, 4.0 Hz, 3 H), 6.88 (dd, *J* = 13.2, 3.3 Hz, 2 H), 6.47 (s, 1 H), 3.70 (dq, *J* = 13.5, 6.9 Hz, 1 H), 3.58 (dq, *J* = 13.3, 6.6 Hz, 1 H), 2.84 (t, *J* = 6.9 Hz, 2 H), 2.55 (s, 3 H). ^13^C NMR (100 MHz, CDCl_3_) δ 167.84, 155.51, 148.63, 138.24, 134.53, 129.36, 129.18, 128.99, 128.71, 128.57, 127.60, 126.51, 124.25, 124.16, 121.03, 120.87, 119.28, 117.68, 117.42, 59.24, 40.98, 35.54, 14.55. HRMS (ESI) m/z calcd for C_27_H_25_N_4_O_2_^+^ (M + H)^+^ 437.1972, found 437.1966.

### Measurement of cell viability and proliferation

Human colon cancer cell lines HCT116 and SW620 were cultured under standard culture conditions at 37 °C and 5% CO_2_ atmosphere in the specific medium (McCOY’S 5 A and DMEM) supplemented with 10% fetal bovine serum, 100 UI/mL penicillin and 100 mg/L streptomycin. The short-term effects of compounds **7** and **14** on cell growth were measured with the MTT assay. Briefly, HCT116 and SW620 cells were seeded into 96-well plates (3,000 cells/well) and incubated overnight at 37 °C, then treated with 0, 0.2, 0.4, 0.8, 1.6, 3.2, 6.4 and 12.8 μmol/L test compound for 24, 48 and 72 h. Next, 20 μL MTT solution (5 mg/mL) was added into each well and incubated for another 4 h followed by media removal and solubilization in 200 μL DMSO. The absorbance value was determined at 570 nm using a microplate reader (Bio-Tek, Winooski, VT, USA). The long-term effects of compounds **7** and **14** on cell growth were assessed with a clone formation assay. Cells were cultured at a density of 200 cells per well in 6-well plates for 36 h. Then, different concentrations of escin (0, 1, 2 and 4 μmol/L) were added. Cells were cultured for approximately 14 d until the cells grew visible colonies. The medium was discarded, and the colonies were stained with crystal violet (C0121, Beyotime) for 15 min at room temperature. After carefully washing with PBS, the stained colonies in each well were photographed and the number of colonies with more than 50 cells was counted manually.

### Flow cytometry analysis

HCT116 and SW620 were exposed to different concentrations of compound **7h** for 24 h, and then operated in flow cytometry analysis. Briefly, the harvested cells were fixed with 70% cold ethanol in 4 °C for 24 h. Then, the fixed cells were incubated in 200 μL PBS solution containing 1 μL propidium iodide (BD, CA, USA) and 1 μL RNase (Sigma Aldrich, USA). Finally, cells were analyzed using BD Accuri^TM^ C6 flow cytometry (BD Biosciences, USA). The FlowJo software was used to analyze the cell cycle arrest.

## Supplementary information

Supplementary information.

## References

[1] Tong, S., Wang, Q., Wang, M.-X. & Zhu, J. Tuning the reactivity of isocyano group: synthesis of imidazoles and imidazoliums from propargylamines and isonitriles in the presence of multiple catalysts. *Angew. Chem. Int. Ed.* **54**, 1293–1300, 10.1002/anie.201410113 (2015). 10.1002/anie.201410113 25430618

[2] Zajdlik A (2013). α-Boryl isocyanides enable facile preparation of bioactive boropeptides. Angew. Chem. Int. Ed..

[3] Dömling A, Wang W, Wang K (2012). Chemistry and biology of multicomponent reactions. Chem. Rev..

[4] Lei J (2018). Recent advances in the development of polycyclic skeletons *via* Ugi reaction cascades. Mol. Divers..

[5] Zhao W, Huang L, Guan Y, Wulff WD (2014). Three-Component Asymmetric Catalytic Ugi Reaction-Concinnity from Diversity by Substrate-Mediated Catalyst Assembly. Angew. Chem. Int. Ed..

[6] Chandgude AL, Dömling A (2017). *N*-Hydroxyimide Ugi Reaction toward α-Hydrazino Amides. Org. Lett..

[7] Xu Z, Shaw AY, Nichol G, Cappelli AP, Hulme C (2012). Applications of ortho-phenylisonitrile and ortho-N-Boc aniline for the two-step preparation of novel bis-heterocyclic chemotypes. Mol. Divers..

[8] Zhang Y (2016). Chiral Phosphoric Acid Catalyzed Asymmetric Ugi Reaction by Dynamic Kinetic Resolution of the Primary Multicomponent Adduct. Angew. Chem. Int. Ed..

[9] Igawa H (2016). Amine-free melanin-concentrating hormone receptor 1 antagonists: Novel 1-(1*H*-benzimidazol-6-yl)pyridin-2(1*H*)-one derivatives and design to avoid CYP3A4 time-dependent inhibition. Bioorg. Med. Chem..

[10] Thomé I, Besson C, Kleine T, Bolm C (2013). Base‐Catalyzed Synthesis of Substituted Indazoles under Mild, Transition‐Metal‐Free Conditions. Angew. Chem. Int. Ed..

[11] Jones ED (2010). Design of a series of bicyclic HIV-1 integrase inhibitors. Part 1: Selection of the scaffold. Bioorg. Med. Chem. Lett..

[12] Bischoff F (2012). Design and Synthesis of a Novel Series of Bicyclic Heterocycles As Potent *γ*-Secretase Modulators. J. Med. Chem..

[13] Bridges KA (2011). MK-1775, a Novel Wee1 Kinase Inhibitor, Radiosensitizes p53-Defective Human Tumor Cells. Clin. Cancer Res..

[14] Rajeshkumar NV (2011). MK-1775, a Potent Wee1 Inhibitor, Synergizes with Gemcitabine to Achieve Tumor Regressions, Selectively in p53-Deficient Pancreatic Cancer Xenografts. Clin. Cancer Res..

[15] Jones P (2009). Discovery of 2-{4-[(3*S*)-Piperidin-3-yl]phenyl}-2*H*-indazole-7-carboxamide (MK-4827): A Novel Oral Poly(ADP-ribose)polymerase (PARP) Inhibitor Efficacious in BRCA-1 and -2 Mutant Tumors. J. Med. Chem..

[16] Wang L (2012). MK-4827, a PARP-1/-2 inhibitor, strongly enhances response of human lung and breast cancer xenografts to radiation. Invest. New Drugs.

[17] Schröder F (2015). Supported gold nanoparticles as efficient and reusable heterogeneous catalyst for cycloisomerization reactions. Green Chem..

[18] Cai H, Thombal RS, Li X, Lee YR (2019). Rhodium(III)‐Catalyzed Regioselective C–H Activation/Annulation for the Diverse Pyrazole‐Core Substituted Furans. Adv. Synth. Catal..

[19] Kim, H., Thombal, R. S., Khanal, H. D. & Lee, Y. R. Rhodium(iii)-catalyzed regioselective distal *ortho* C–H alkenylation of *N*-benzyl/furanylmethylpyrazoles directed by *N*-coordinating heterocycles. *Chem. Commun.* **55**, 13402–13405, 10.1039/C9CC06758B (2019). 10.1039/c9cc06758b 31637396

[20] Nagaradja, E. *et al*. Deproto-metallation using a mixed lithium–zinc base and computed CH acidity of 1-aryl 1*H*-benzotriazoles and 1-aryl 1*H*-indazoles. *Org. Biomol. Chem.* **12**, 1475–1487, 10.1039/C3OB42380H (2014). 10.1039/c3ob42380h 24445663

[21] Lin M-H, Liu H-J, Lin W-C, Kuo C-K, Chuang T-H (2015). Regioselective synthesis of 2H-indazoles through Ga/Al- and Al-mediated direct alkylation reactions of indazoles. Org. Biomol. Chem..

[22] Balwe, S. G., Shinde, V. V., Rokade, A. A., Park, S. S. & Jeong, Y. T. Green synthesis and characterization of silver nanoparticles (Ag NPs) from extract of plant Radix Puerariae: An efficient and recyclable catalyst for the construction of pyrimido[1,2-*b*]indazole derivatives under solvent-free conditions. *Catal. Commun.* **99**, 121–126, 10.1016/j.catcom.2017.06.006 (2017).

[23] Ankamwar, B., Damle, C., Ahmad, A. & Sastry, M. Biosynthesis of gold and silver nanoparticles using Emblica officinalis fruit extract, their phase transfer, transmetallation in an organic solution. *J. Nanosci. Nanotechnol.* **5**, 1665–1671, 10.1166/jnn.2005.184 (2005). 10.1166/jnn.2005.184 16245525

[24] Palaniraja, J., Roopan, S. M. & Rayalu, G. M. One-pot synthesis of highly functionalized pyrimido[1,2-*b*]indazoles via 6-endo-dig cyclization. *RSC Adv.* **6**, 24610–24616, 10.1039/C6RA02596J (2016).

[25] Raj T (2017). “Solvent-Less” Mechanochemical Approach to the Synthesis of Pyrimidine Derivative. ACS Sustainable Chem. Eng..

[26] Kong D, Lu G, Wu M, Shi Z, Lin Q (2017). One-Pot, Catalyst-Free Synthesis of Spiro[dihydroquinolinenaphthofuranone] Compounds from Isatins in Water Triggered by Hydrogen Bonding Effects. ACS Sustainable Chem. Eng..

[27] Zhang M (2017). Catalyst-Free, Visible-Light Promoted One-Pot Synthesis of Spirooxindole-Pyran Derivatives in Aqueous Ethyl Lactate. ACS Sustainable Chem. Eng..

[28] Liao W (2016). An Efficient and Facile Method for the Synthesis of Benzimidazoisoquinoline Derivatives via a Multicomponent Reaction. ACS Comb. Sci..

[29] Lei J (2016). Synthesis of isoindolin-1-one derivatives via multicomponent reactions of methyl 2-formylbenzoate and intramolecular amidation. Mol. Diver..

[30] Yu X, Xin X, Wan B, Li X (2013). Base-Catalyzed Cyclization of *N*-Sulfonyl Propargylamides to Sulfonylmethyl-Substituted Oxazoles via Sulfonyl. Migration. J. Org. Chem..

[31] García–González, M. C., Hernández-Vázquez, E., Gordillo-Cruz, R. E. & Miranda, L. D. Ugi-derived dehydroalanines as a pivotal template in the diversity oriented synthesis of aza-polyheterocycles. *Chem. Commun.* **51**, 11669–11672, 10.1039/C5CC02927A (2015). 10.1039/c5cc02927a 26102372

[32] Icelo-Ávila E, Amador-Sánchez YA, Polindara-García LA, Miranda LD (2017). Synthesis of 6-methyl-3,4-dihydropyrazinones using an Ugi 4-CR/allenamide cycloisomerization protocol. Org. Biomol. Chem..

[33] Shafakat Ali NA, Ahmad Dar B, Pradhan V, Farooqui M (2013). Chemistry and Biology of Indoles and Indazoles: A Mini-Review. Mini-Rev. Med. Chem..

[34] Abdelraheem EMM, Kurpiewska K, Kalinowska-Tłuścik J, Dömling A (2016). Artificial Macrocycles by Ugi Reaction and Passerini Ring Closure. J. Org. Chem..

[35] Mundal DA, Lutz KE, Thomson RJ (2012). A Direct Synthesis of Allenes by a Traceless Petasis Reaction. J. Am. Chem. Soc..

[36] Dong Y (2005). An Efficient Kinetic Resolution of Racemic Betti Base Based on an Enantioselective *N,O*-Deketalization. J. Org. Chem..

[37] Bhagat S, Chakraborti AK (2007). An Extremely Efficient Three-Component Reaction of Aldehydes/Ketones, Amines, and Phosphites (Kabachnik-Fields Reaction) for the Synthesis of α-Aminophosphonates Catalyzed by Magnesium Perchlorate. J. Org. Chem..

[38] Shaaban S, Abdel-Wahab BF (2016). Groebke–Blackburn–Bienaymé multicomponent reaction: emerging chemistry for drug discovery. Mol. Divers..

