# Supplementary material for: Catalyst-Free One-Pot Synthesis of Densely Substituted Pyrazole-Pyrazines as Anti-Colorectal Cancer Agents
Source: Sci Rep. 2020 Jun 9;10:9281. doi: 10.1038/s41598-020-66137-z (PMC7283261; doi:10.1038/s41598-020-66137-z)
Supplement: Supplementary file 1 — Supplementary information. [file 41598_2020_66137_MOESM1_ESM.pdf]

Supporting Information

Catalyst-Free One-Pot Synthesis of Densely Substituted Pyrazole-Pyrazines as Anti-Colorectal Cancer Agents

Jia Xu[1], Hong-Bo Tan[1], Ya-Jun Zhang[1], Dian-Yong Tang[1], Frank F. Zhan,[3] Hong-yu Li,[2,3]* Zhong-Zhu Chen[1]* & Zhi-Gang Xu[1]*

[1]College of Pharmacy, National & Local Joint Engineering Research Center of Targeted and Innovative Therapeutics, Chongqing Key Laboratory of Kinase Modulators as Innovative Medicine, Chongqing University of Arts and Sciences, Chongqing 402160, China.

[2]Department of Pharmaceutical Sciences, College of Pharmacy, University of Arkansas for Medical Sciences, Little Rock, AR 72205, USA.

[3]Winthrop P. Rockefeller Cancer Institute, University of Arkansas for Medical Sciences, Little Rock, AR 72205, USA.

*email: HLi2@uams.edu; 18883138277@163.com; xzg@cqwu.edu.cn.

Compound **5a**

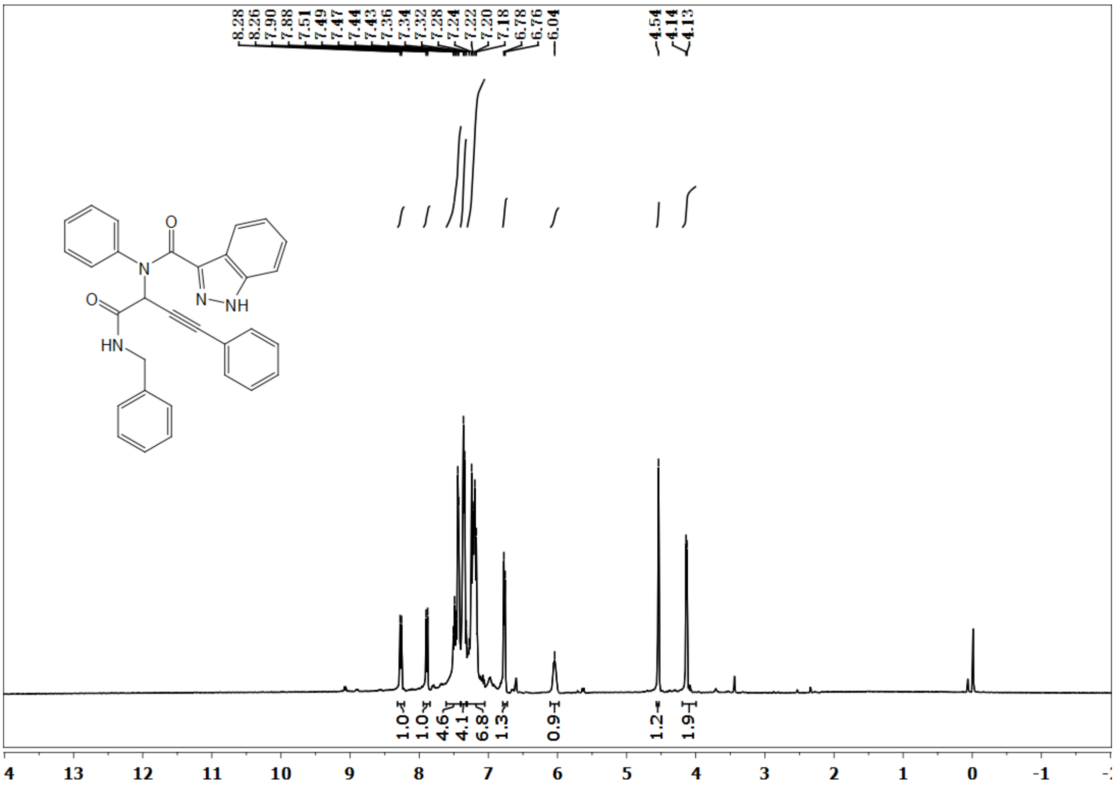

8.28
8.26
7.90
7.88
7.51
7.49
7.47
7.44
7.43
7.36
7.34
7.32
7.28
7.24
7.22
7.20
7.18
6.78
6.76
6.04
4.54
4.14
4.13
1.0
1.0
4.6
4.1
6.8
1.3
0.9
1.2
1.9
O
N
N
NH
O
HN

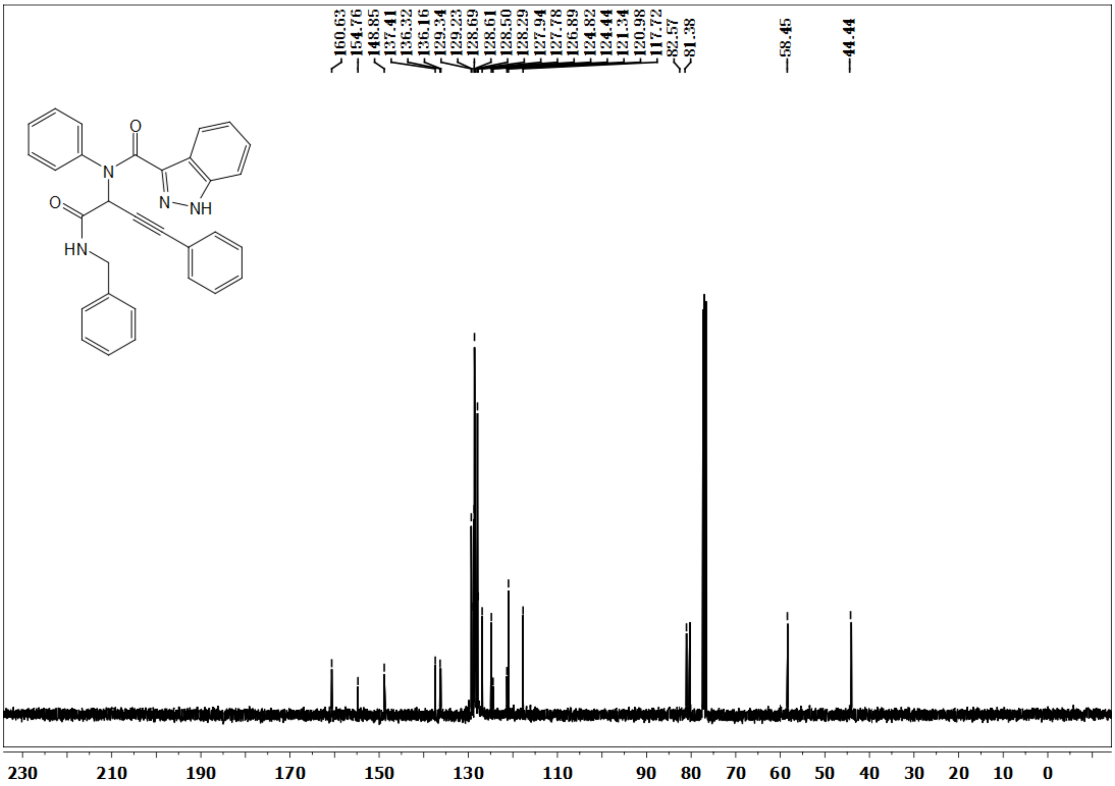

160.63
154.76
148.85
137.41
136.32
136.16
129.34
129.23
128.69
128.61
128.50
128.29
127.94
127.78
126.89
124.82
124.44
121.34
120.98
117.72
82.57
81.38
58.45
44.44
O
N
N
NH
O
HN
230
210
190
170
150
130
110
90
80
70
60
50
40
30
20
10
0

Compound **5b**

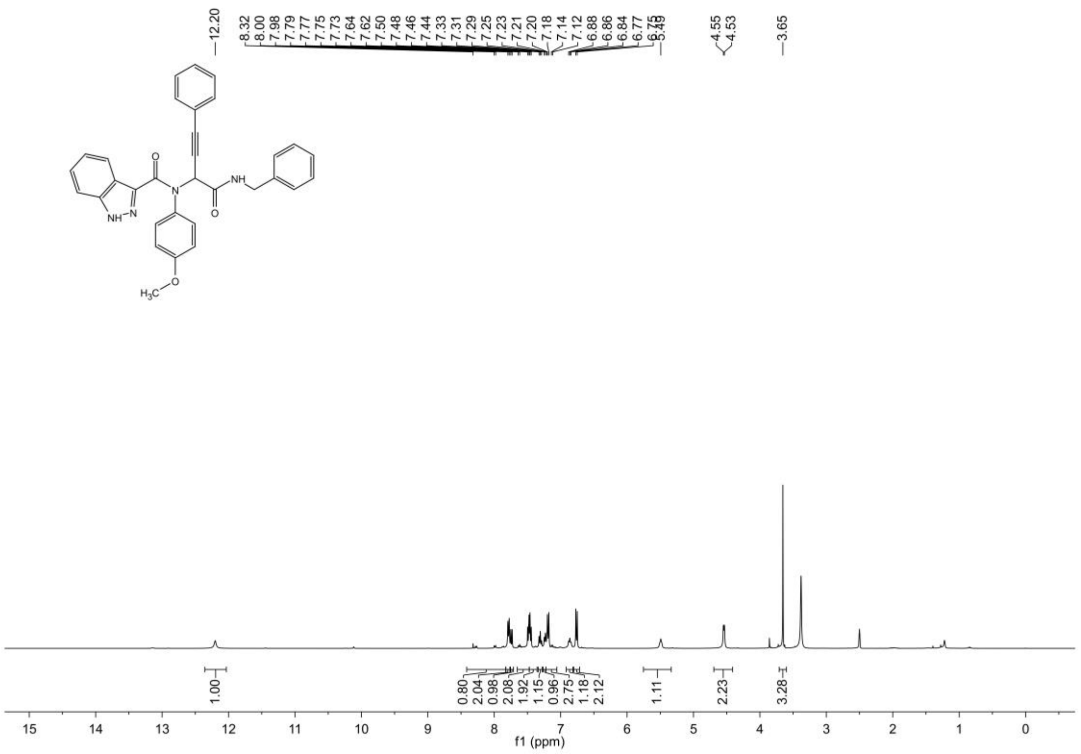
12.20
8.32
8.00
7.98
7.79
7.77
7.75
7.73
7.64
7.62
7.50
7.48
7.46
7.44
7.33
7.31
7.29
7.25
7.23
7.21
7.20
7.18
7.14
7.12
6.88
6.86
6.84
6.77
6.75
5.49
4.55
4.53
3.65
NH
N
O
H3C
1.00
0.80
2.04
0.98
2.08
1.92
1.15
0.96
2.75
1.18
2.12
1.11
2.23
3.28
f1 (ppm)

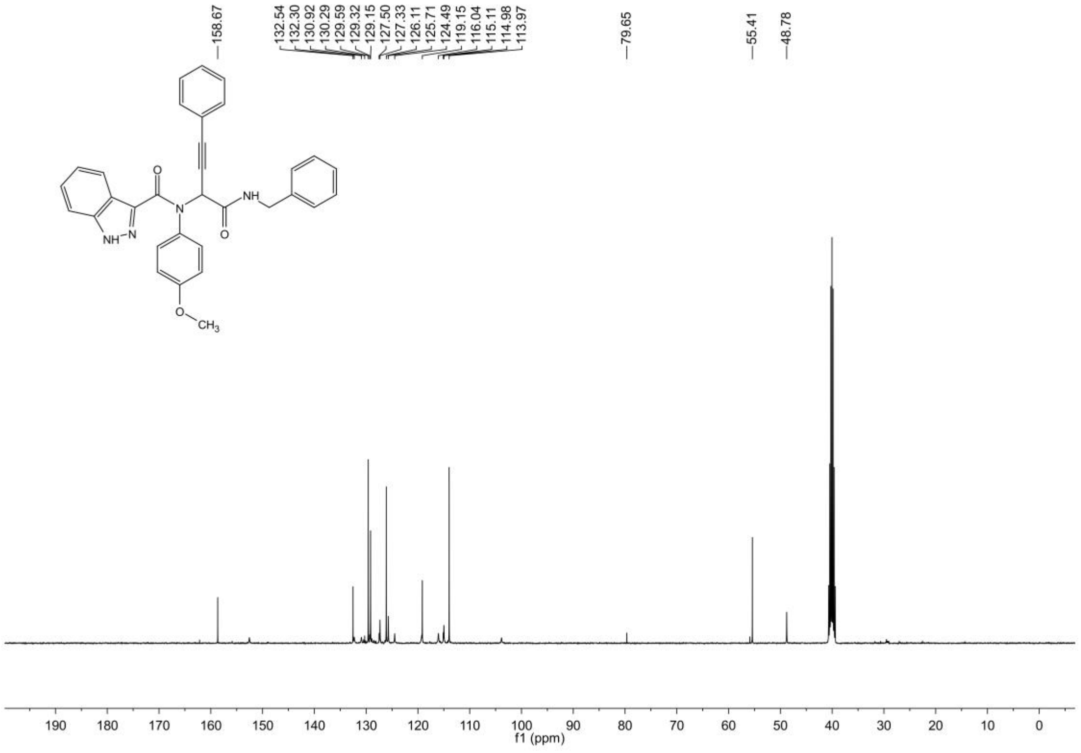
158.67
132.54
132.30
130.92
130.29
129.59
129.32
129.15
127.50
127.33
126.11
125.71
124.49
119.15
116.04
115.11
114.98
113.97
79.65
55.41
48.78
NH
N
O
CH3
f1 (ppm)

Compound **6a**

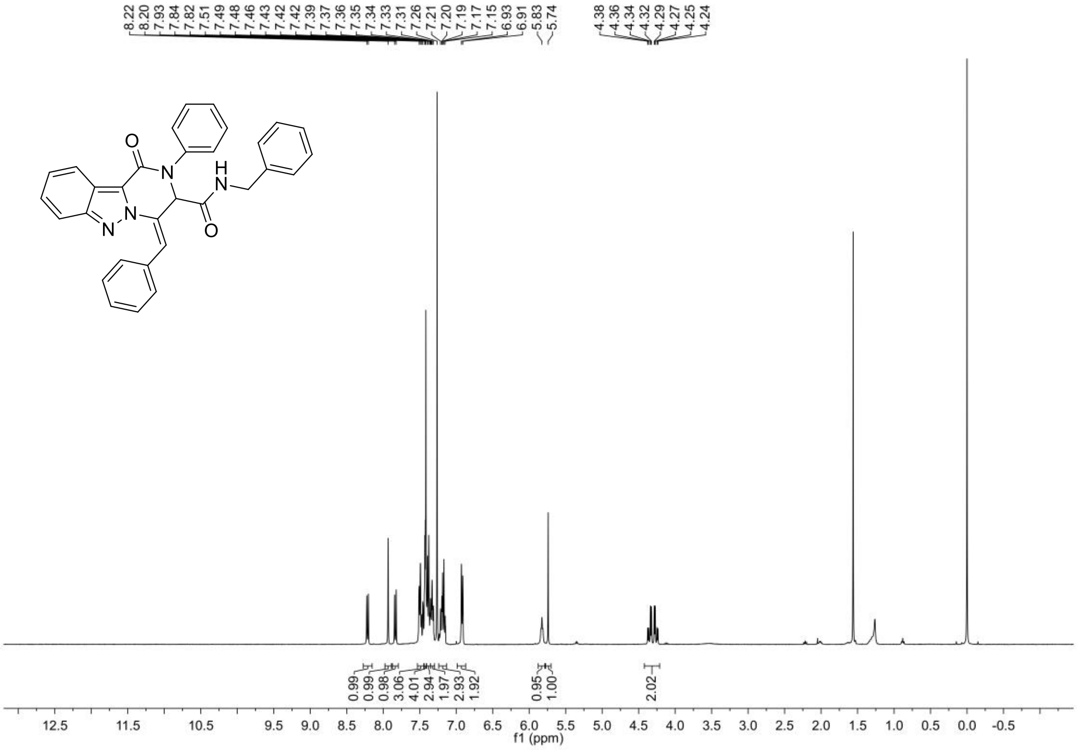
8.22
8.20
7.93
7.84
7.82
7.51
7.49
7.48
7.46
7.43
7.42
7.42
7.39
7.37
7.36
7.35
7.34
7.33
7.31
7.26
7.21
7.20
7.19
7.17
7.15
6.93
6.91
5.83
5.74
4.38
4.36
4.34
4.32
4.29
4.27
4.25
4.24
0.99
0.99
0.98
3.06
4.01
2.94
1.97
2.93
1.92
0.95
1.00
2.02
12.5 11.5 10.5 9.5 9.0 8.5 8.0 7.5 7.0 6.5 6.0 5.5 5.0 4.5 4.0 3.5 3.0 2.5 2.0 1.5 1.0 0.5 0.0 -0.5
f1 (ppm)

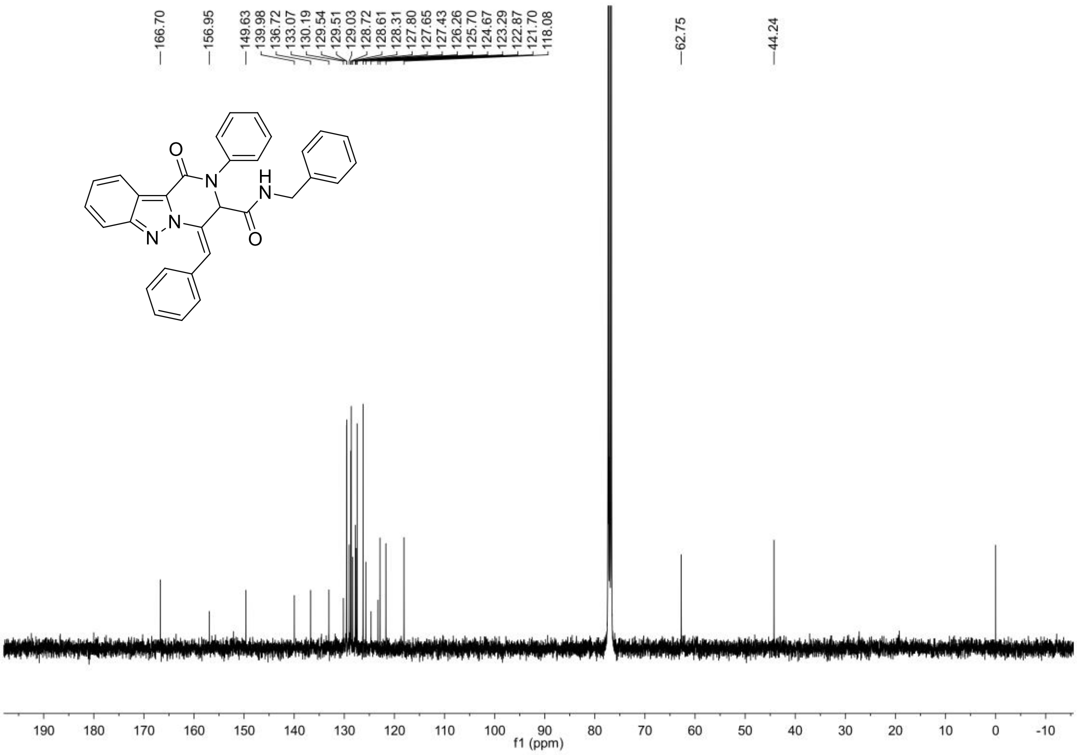
166.70
156.95
149.63
139.98
136.72
133.07
130.19
129.54
129.51
129.03
128.72
128.61
128.31
127.80
127.65
127.43
126.26
125.70
124.67
123.29
122.87
121.70
118.08
62.75
44.24
190 180 170 160 150 140 130 120 110 100 90 80 70 60 50 40 30 20 10 0 -10
f1 (ppm)

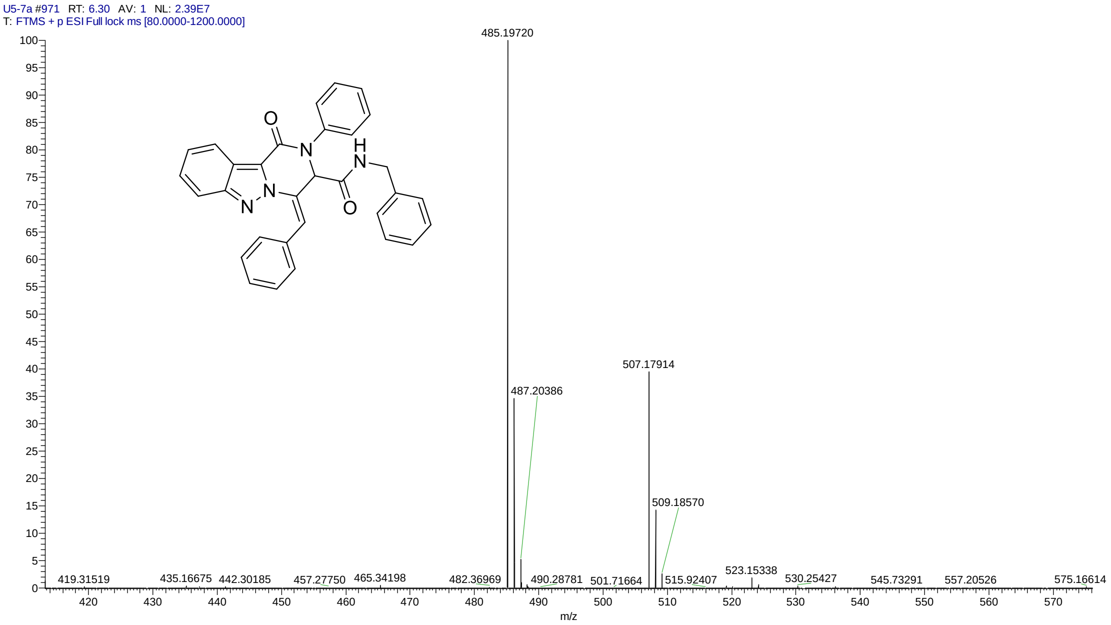
U5-7a #971 RT: 6.30 AV: 1 NL: 2.39E7
T: FTMS + p ESI Full lock ms [80.0000-1200.0000]
485.19720
487.20386
507.17914
509.18570
523.15338
419.31519
435.16675
442.30185
457.27750
465.34198
482.36969
490.28781
501.71664
515.92407
530.25427
545.73291
557.20526
575.16614
m/z
O
N
H
N
N
N
O

Compound **7a**

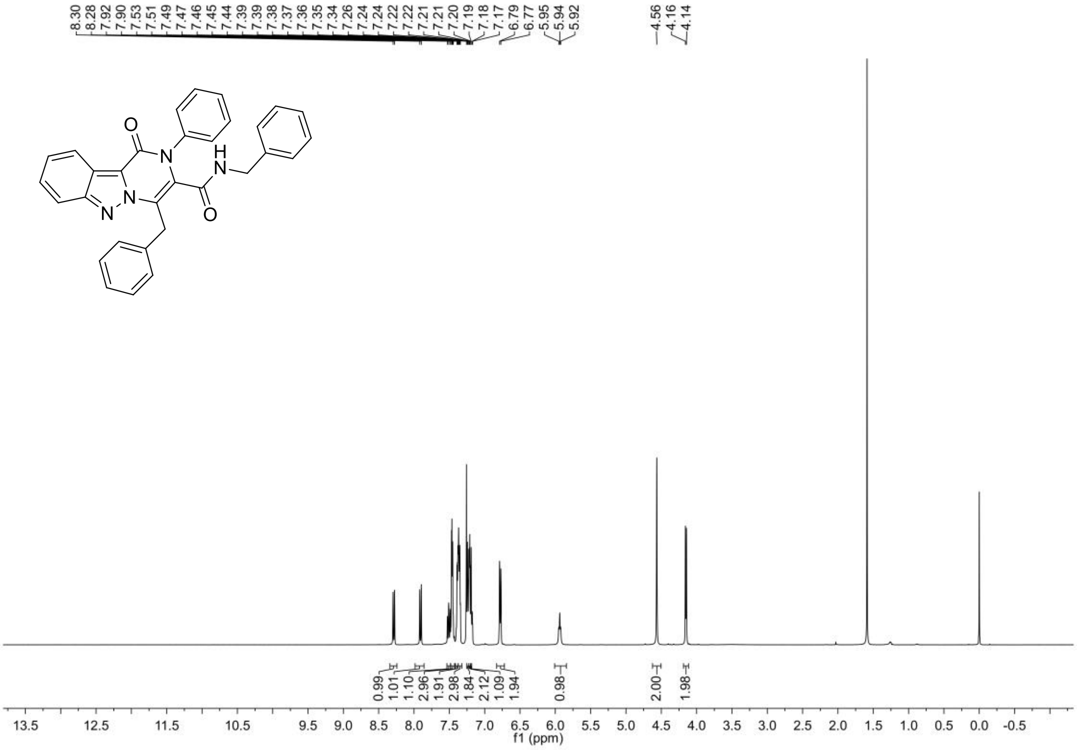
8.30 8.28 7.92 7.90 7.53 7.51 7.49 7.47 7.46 7.45 7.44 7.39 7.39 7.38 7.37 7.36 7.35 7.34 7.26 7.24 7.24 7.22 7.22 7.21 7.21 7.20 7.19 7.18 7.17 6.79 6.77 5.95 5.94 5.92
4.56 4.16 4.14
0.99 1.01 1.10 2.96 1.91 2.98 1.84 2.12 1.09 1.94 0.98 2.00 1.98
f1 (ppm)
O
N
H
N
N
N
O

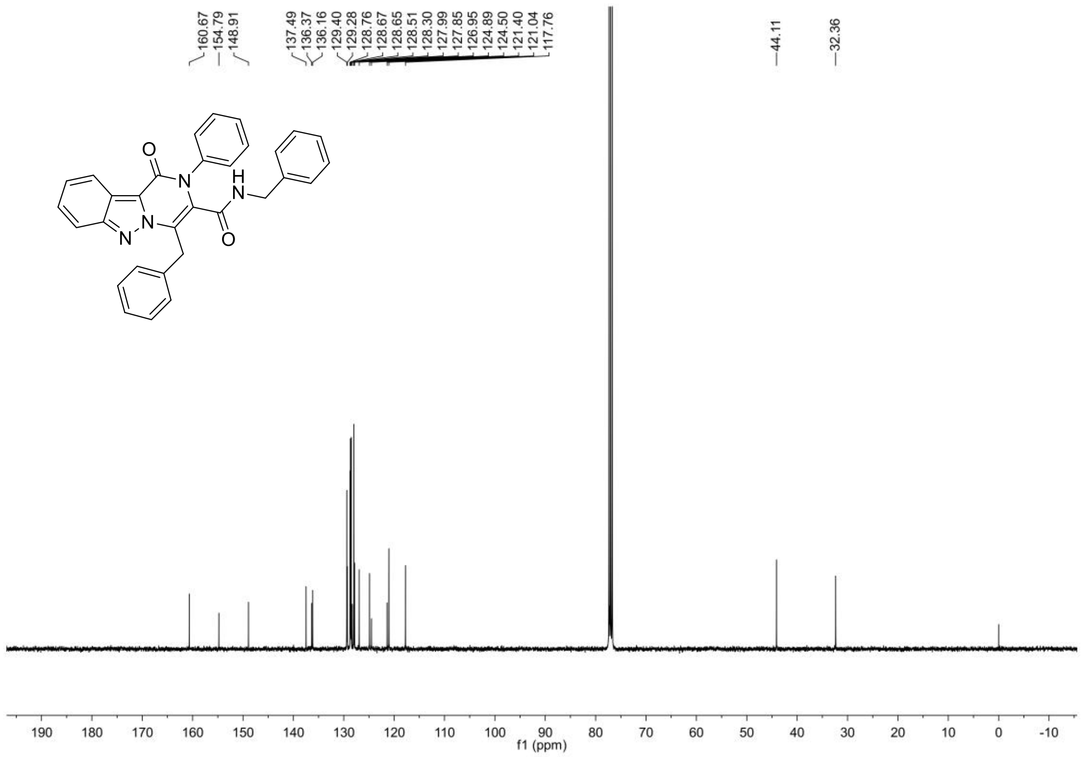
160.67
154.79
148.91
137.49
136.37
136.16
129.40
129.28
128.76
128.67
128.65
128.51
128.30
127.99
127.85
126.95
124.89
124.50
121.40
121.04
117.76
44.11
32.36
O
N
H
N
N
N
O
190
180
170
160
150
140
130
120
110
100
90
80
70
60
50
40
30
20
10
0
-10
f1 (ppm)

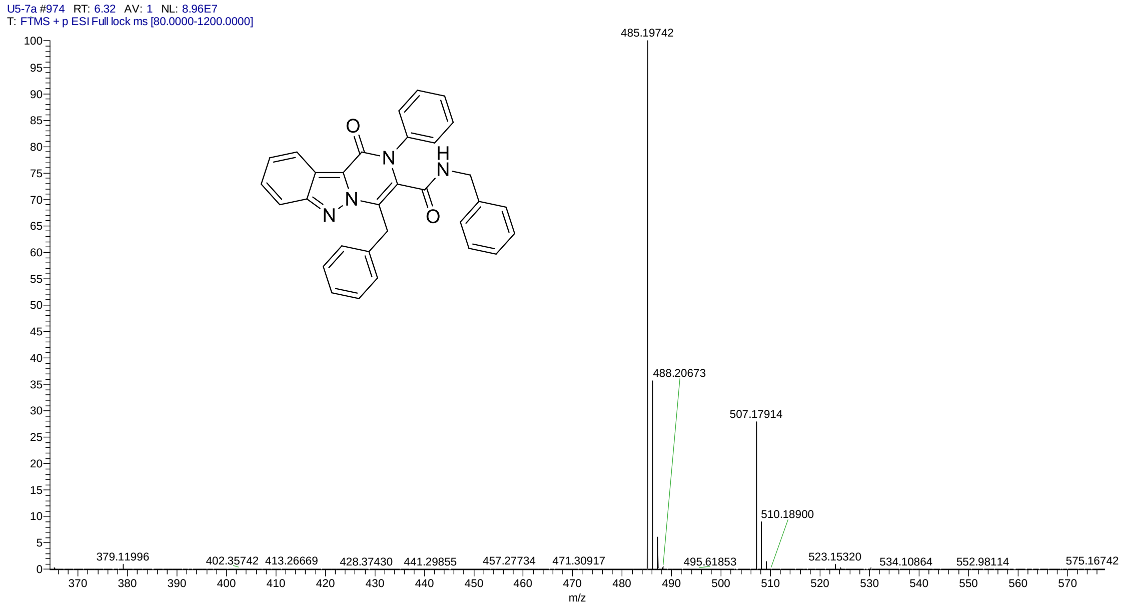
U5-7a #974 RT: 6.32 AV: 1 NL: 8.96E7
T: FTMS + p ESI Full lock ms [80.0000-1200.0000]
485.19742
488.20673
507.17914
510.18900
379.11996
402.35742
413.26669
428.37430
441.29855
457.27734
471.30917
495.61853
523.15320
534.10864
552.98114
575.16742
m/z

Compound **7b**

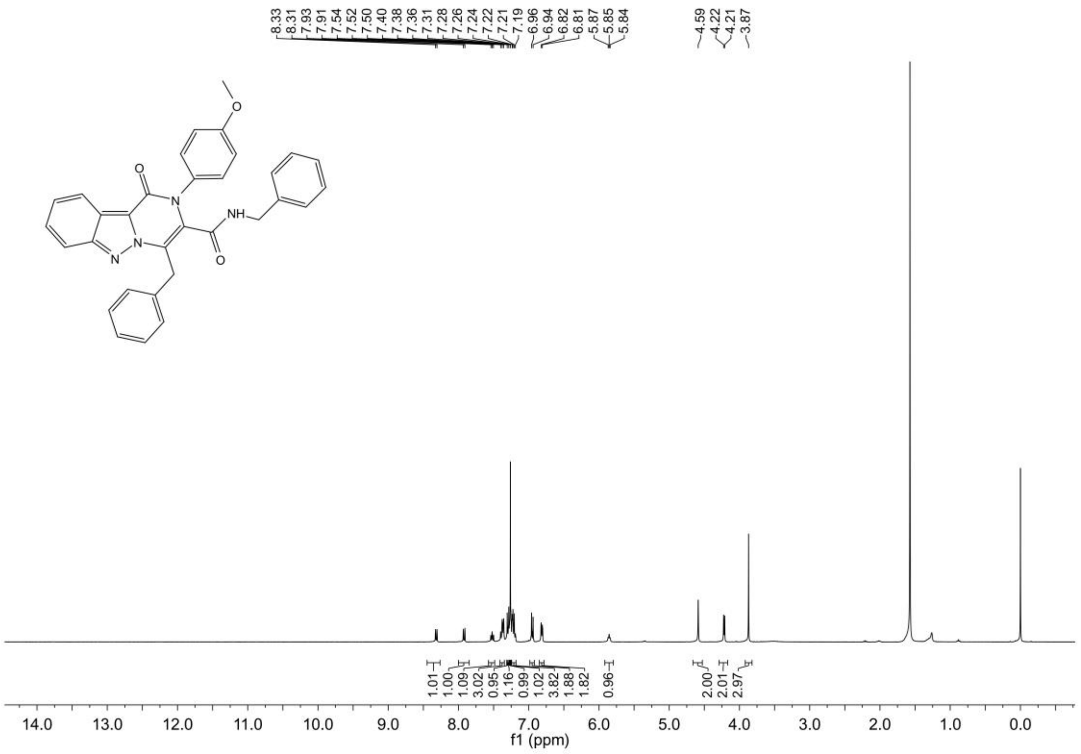
8.33 8.31 7.93 7.91 7.54 7.52 7.50 7.40 7.38 7.36 7.31 7.28 7.26 7.24 7.22 7.21 7.19 6.96 6.94 6.82 6.81 5.87 5.85 5.84
4.59 4.22 4.21 3.87
1.01 1.00 1.09 3.02 0.95 1.16 0.99 1.02 3.82 1.88 1.82 0.96
2.00 2.01 2.97
14.0 13.0 12.0 11.0 10.0 9.0 8.0 7.0 6.0 5.0 4.0 3.0 2.0 1.0 0.0
f1 (ppm)

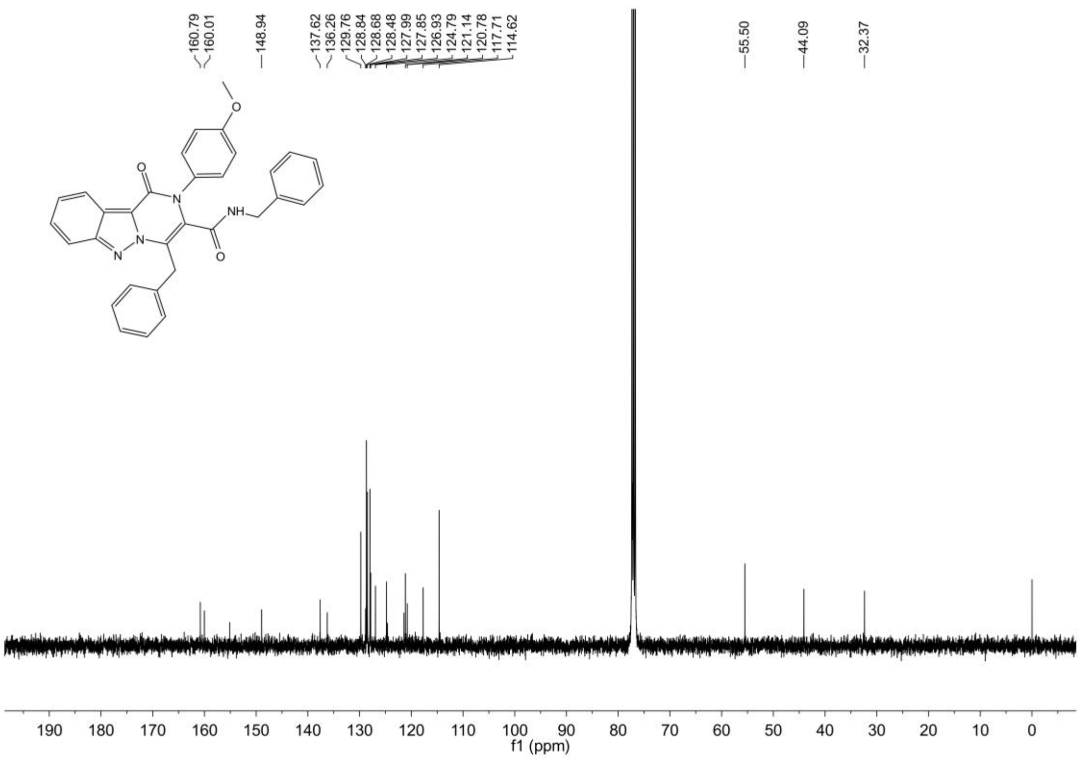
160.79 160.01 148.94
137.62 136.26 129.76 128.84 128.68 128.48 127.99 127.85 126.93 124.79 121.14 120.78 117.71 114.62
55.50 44.09 32.37
190 180 170 160 150 140 130 120 110 100 90 80 70 60 50 40 30 20 10 0
f1 (ppm)

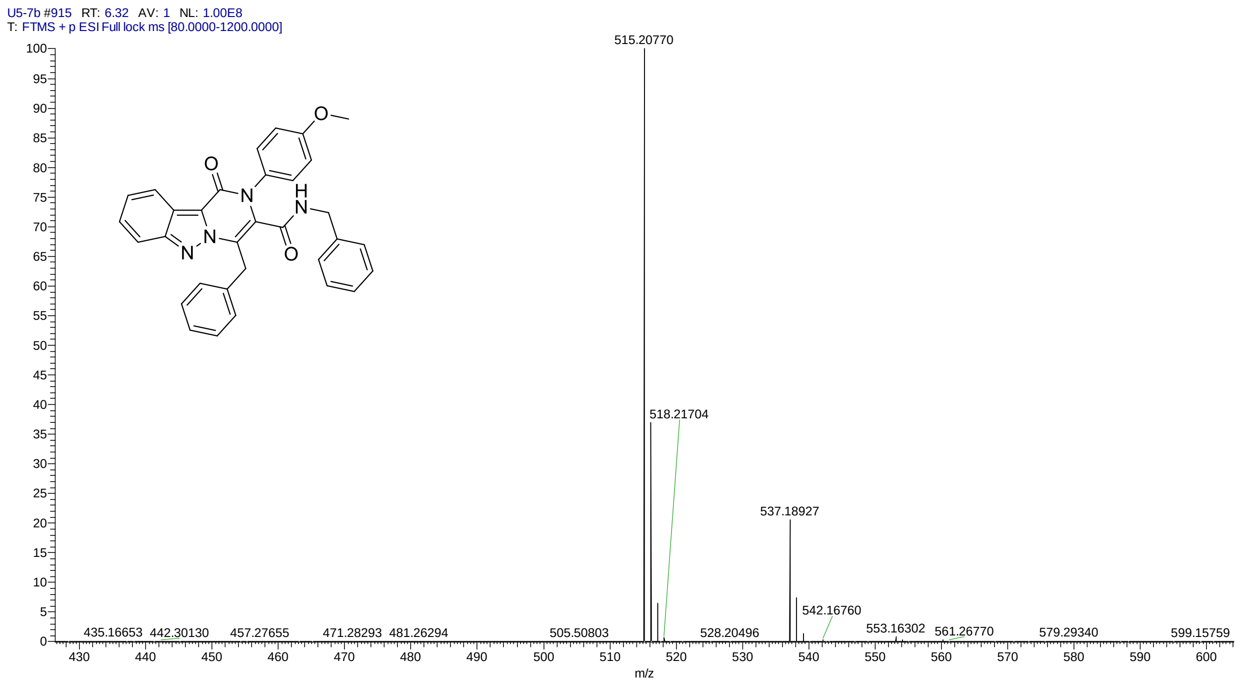

U5-7b #915 RT: 6.32 AV: 1 NL: 1.00E8
T: FTMS + p ESI Full lock ms [80.0000-1200.0000]
515.20770
518.21704
537.18927
542.16760
435.16653
442.30130
457.27655
471.28293
481.26294
505.50803
528.20496
553.16302
561.26770
579.29340
599.15759
m/z

Compound **7c**

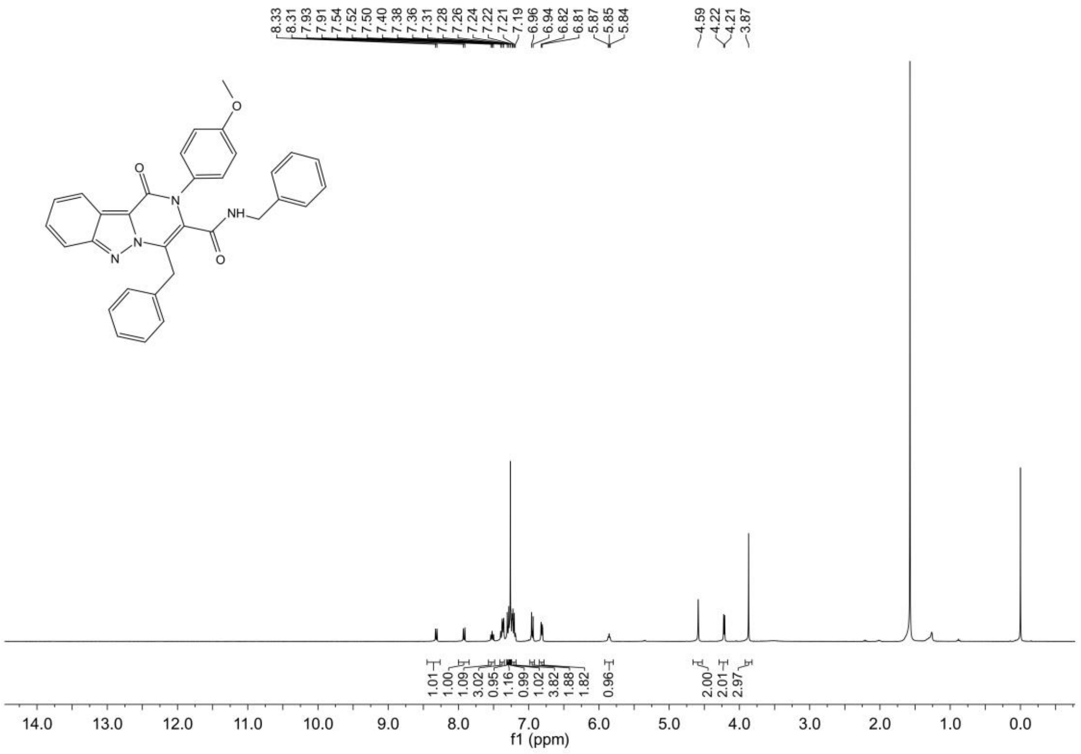

8.33
8.31
7.93
7.91
7.54
7.52
7.50
7.40
7.38
7.36
7.31
7.28
7.26
7.24
7.22
7.21
7.19
6.96
6.94
6.82
6.81
5.87
5.85
5.84
4.59
4.22
4.21
3.87
1.01
1.00
1.09
3.02
0.95
1.16
0.99
1.02
3.82
1.88
1.82
0.96
2.00
2.01
2.97
f1 (ppm)

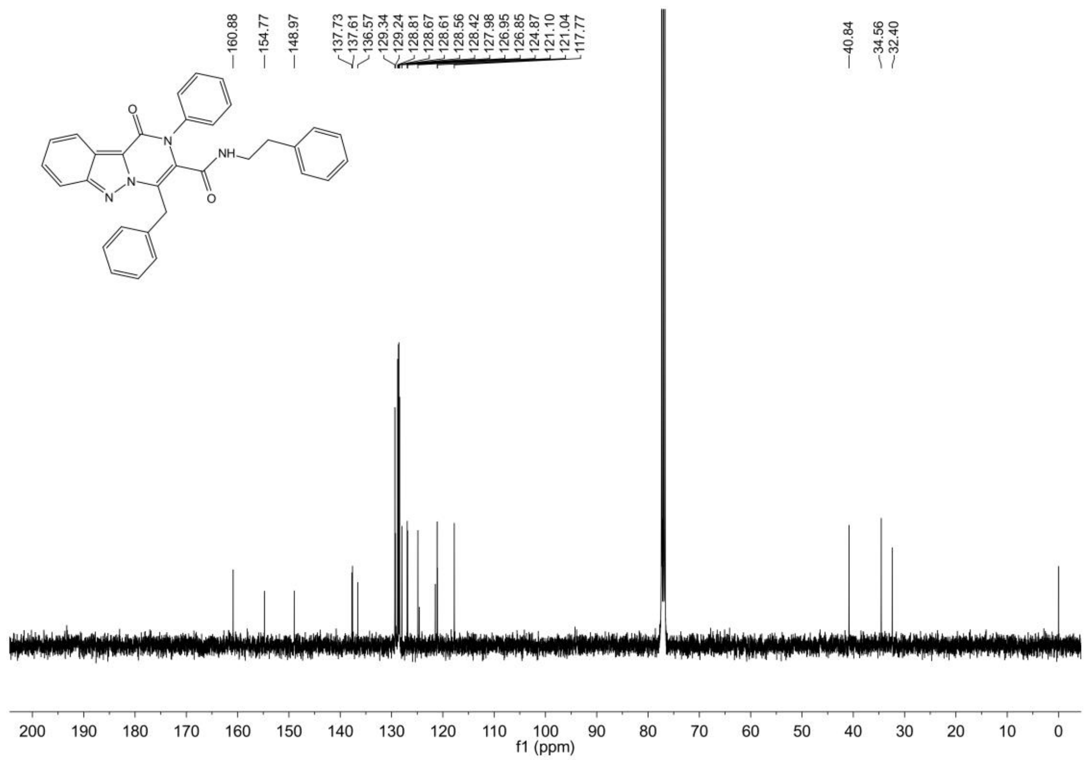
160.88
154.77
148.97
137.73
137.61
136.57
129.34
129.24
128.81
128.67
128.61
128.56
128.42
127.98
126.95
126.85
124.87
121.10
121.04
117.77
40.84
34.56
32.40
O
N
NH
N
N
O
200 190 180 170 160 150 140 130 120 110 100 90 80 70 60 50 40 30 20 10 0
f1 (ppm)

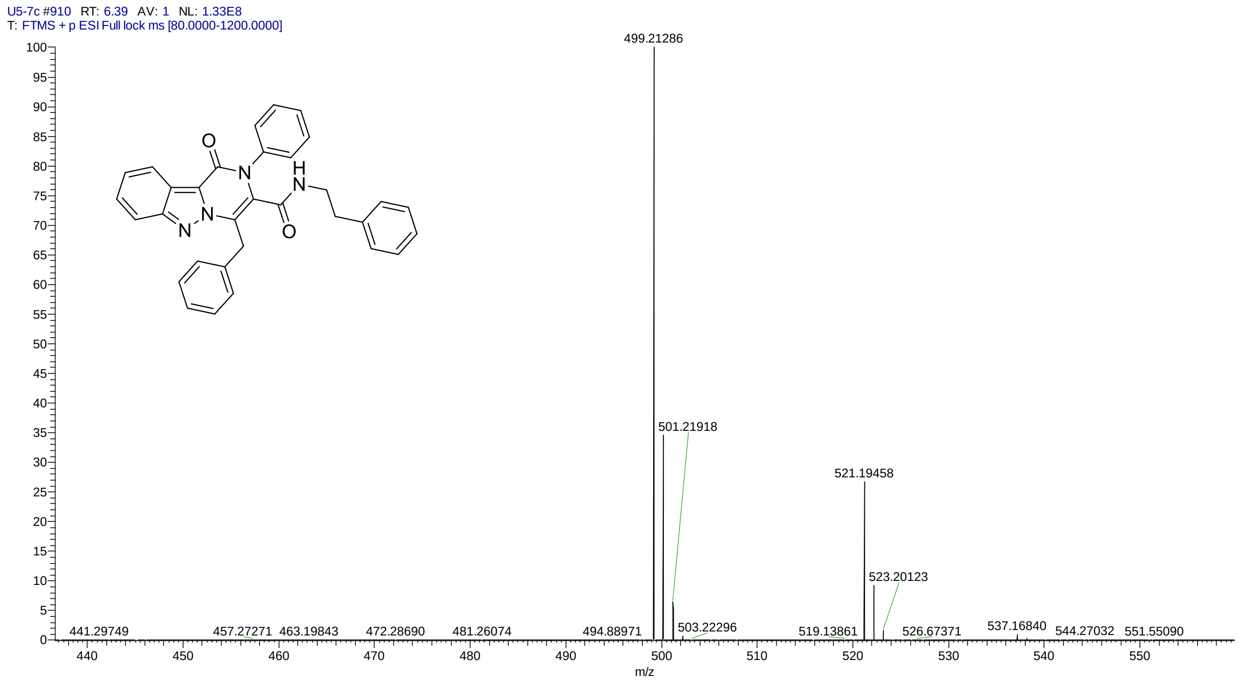
U5-7c #910 RT: 6.39 AV: 1 NL: 1.33E8
T: FTMS + p ESI Full lock ms [80.0000-1200.0000]
499.21286
501.21918
521.19458
523.20123
441.29749
457.27271
463.19843
472.28690
481.26074
494.88971
503.22296
519.13861
526.67371
537.16840
544.27032
551.55090
m/z

Compound **7d**

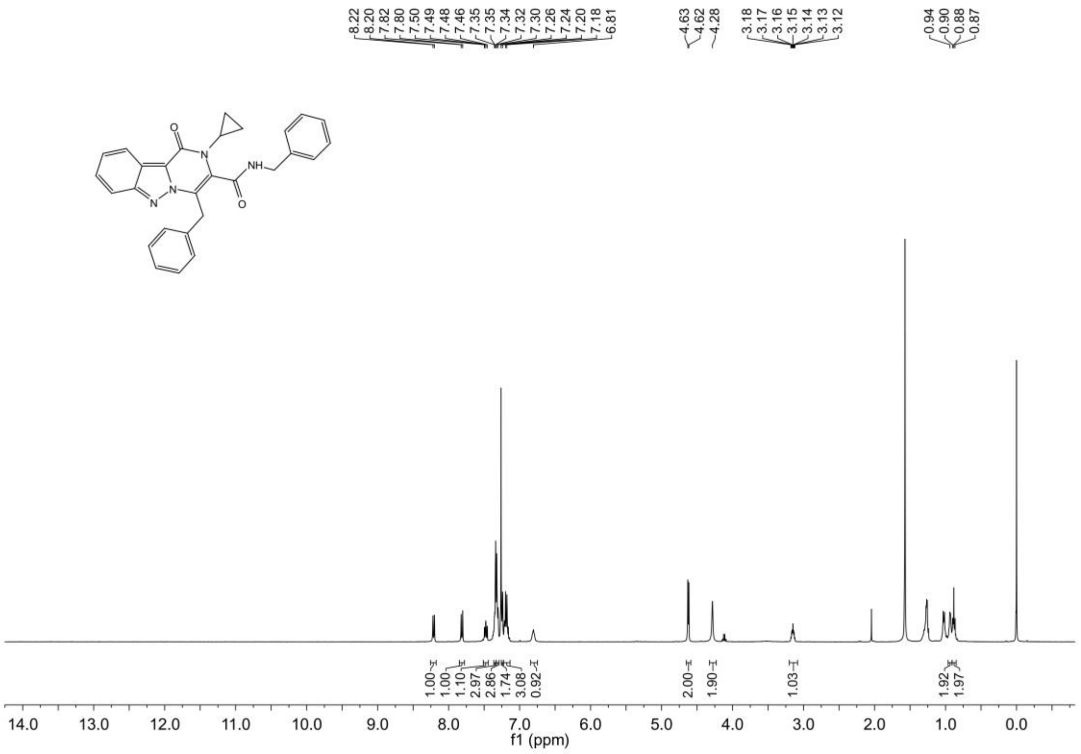

8.22
8.20
7.82
7.80
7.50
7.49
7.48
7.46
7.35
7.35
7.34
7.32
7.30
7.26
7.24
7.20
7.18
6.81
4.63
4.62
4.28
3.18
3.17
3.16
3.15
3.14
3.13
3.12
0.94
0.90
0.88
0.87
1.00
1.00
1.10
2.97
2.86
1.74
3.08
0.92
2.00
1.90
1.03
1.92
1.97
14.0 13.0 12.0 11.0 10.0 9.0 8.0 7.0 6.0 5.0 4.0 3.0 2.0 1.0 0.0
f1 (ppm)

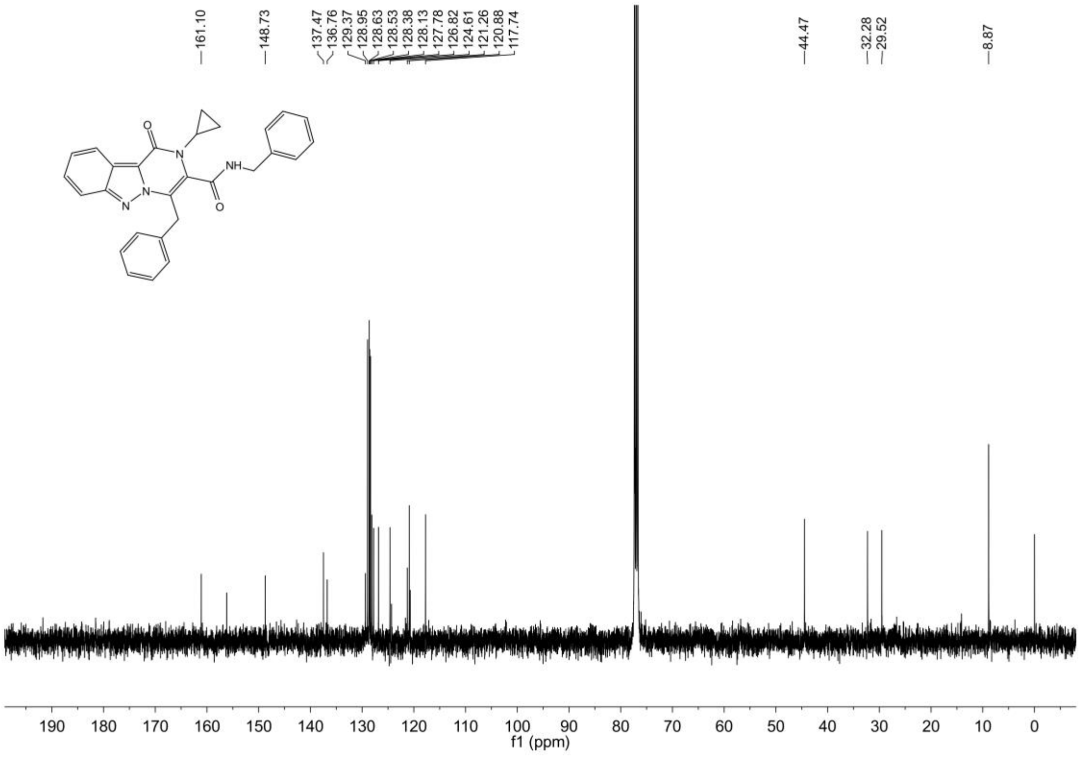

161.10
148.73
137.47
136.76
129.37
128.95
128.63
128.53
128.38
128.13
127.78
126.82
124.61
121.26
120.88
117.74
44.47
32.28
29.52
8.87
190 180 170 160 150 140 130 120 110 100 90 80 70 60 50 40 30 20 10 0
f1 (ppm)

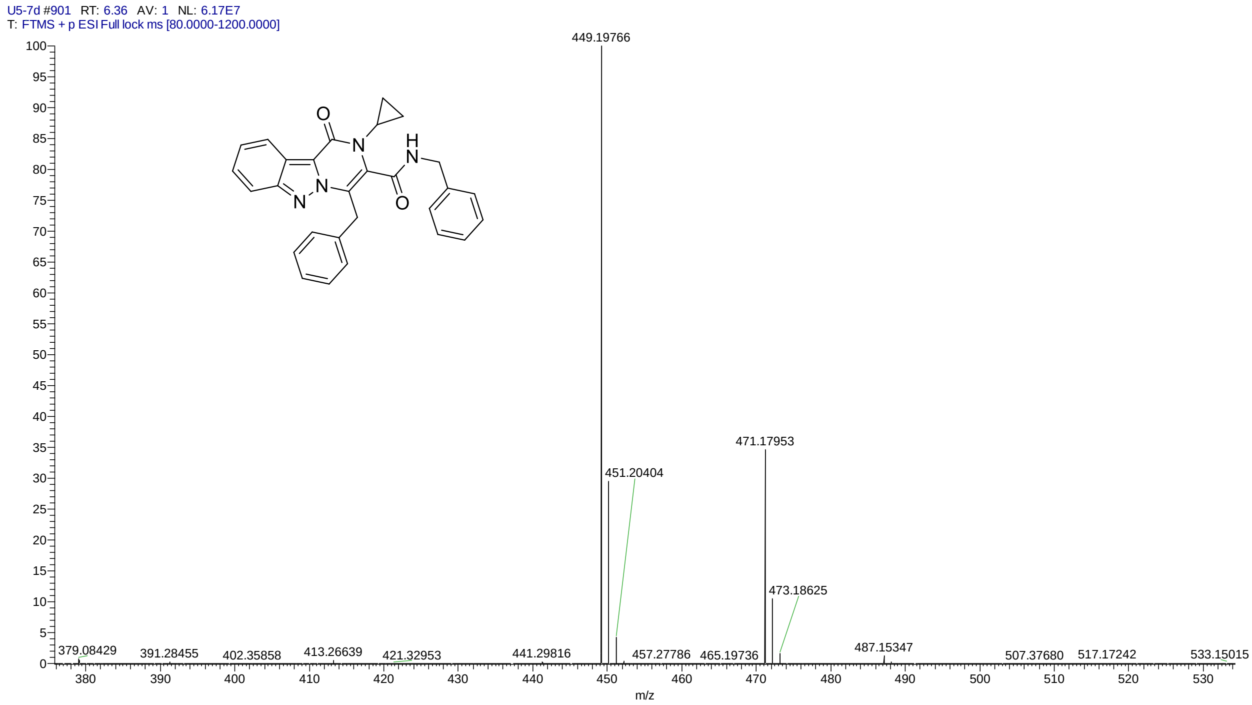
U5-7d #901 RT: 6.36 AV: 1 NL: 6.17E7
T: FTMS + p ESI Full lock ms [80.0000-1200.0000]
449.19766
471.17953
451.20404
473.18625
379.08429
391.28455
402.35858
413.26639
421.32953
441.29816
457.27786
465.19736
487.15347
507.37680
517.17242
533.15015
m/z

Compound **7e**

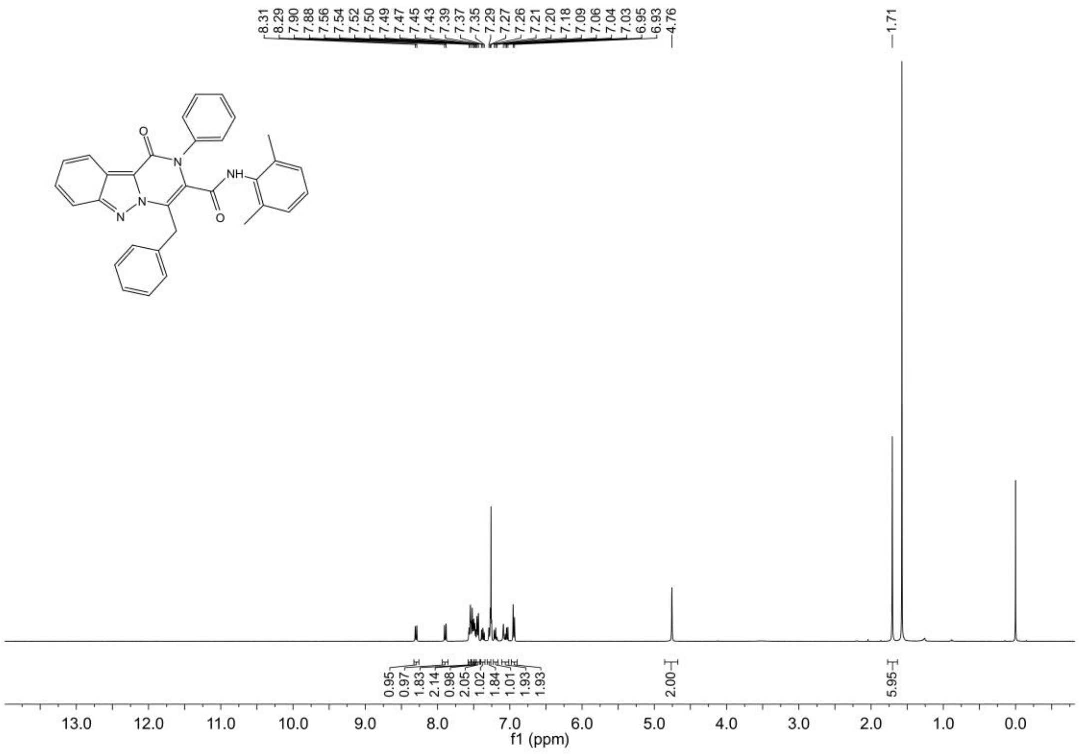
8.31
8.29
7.90
7.88
7.56
7.54
7.52
7.50
7.49
7.47
7.45
7.43
7.39
7.37
7.35
7.29
7.27
7.26
7.21
7.20
7.18
7.09
7.06
7.04
7.03
6.95
6.93
4.76
1.71
0.95
0.97
1.83
2.14
0.98
2.05
1.02
1.84
1.01
1.93
1.93
2.00
5.95
f1 (ppm)

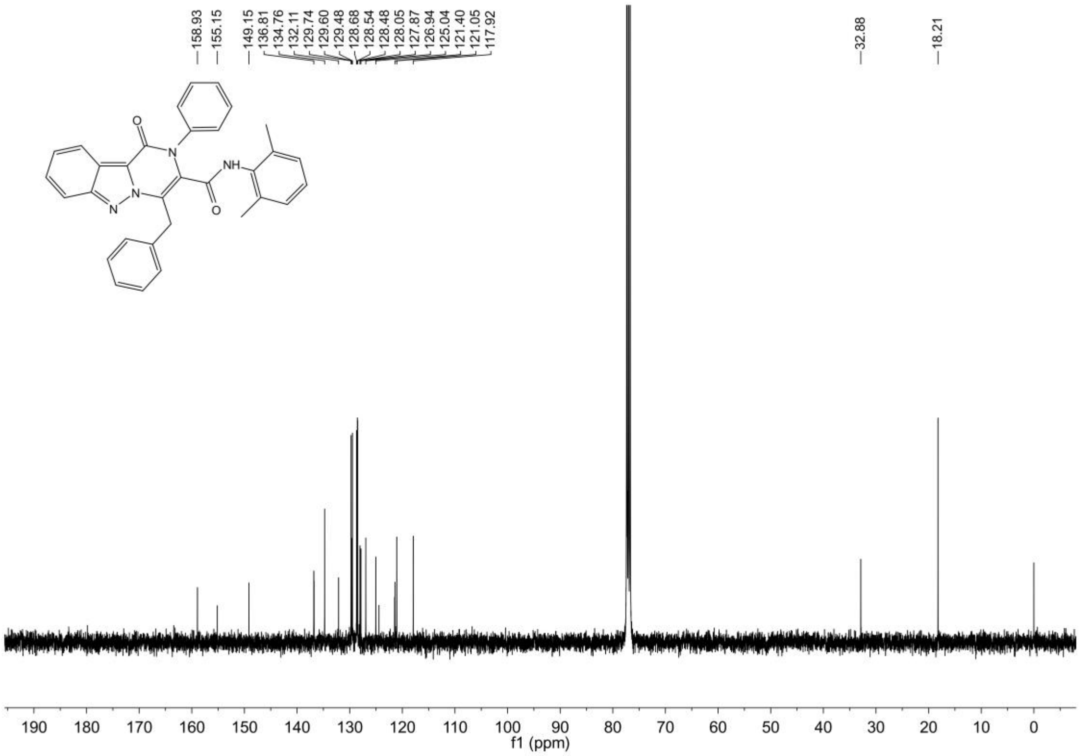
158.93
155.15
149.15
136.81
134.76
132.11
129.74
129.60
129.48
128.68
128.54
128.48
128.05
127.87
126.94
125.04
121.40
121.05
117.92
32.88
18.21
O
N
NH
N
N
O
190 180 170 160 150 140 130 120 110 100 90 80 70 60 50 40 30 20 10 0
f1 (ppm)

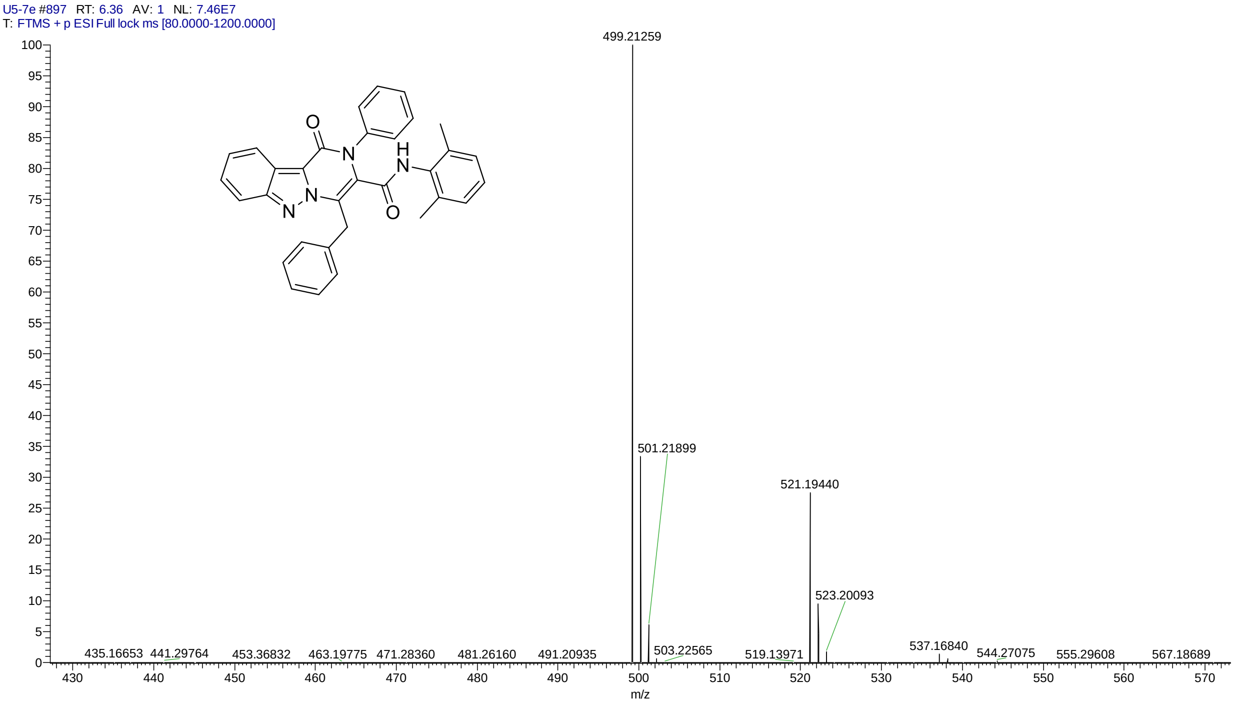
U5-7e #897 RT: 6.36 AV: 1 NL: 7.46E7
T: FTMS + p ESI Full lock ms [80.0000-1200.0000]
499.21259
501.21899
521.19440
523.20093
435.16653
441.29764
453.36832
463.19775
471.28360
481.26160
491.20935
503.22565
519.13971
537.16840
544.27075
555.29608
567.18689
O
N
H
N
N
N
O
m/z

Compound **7f**

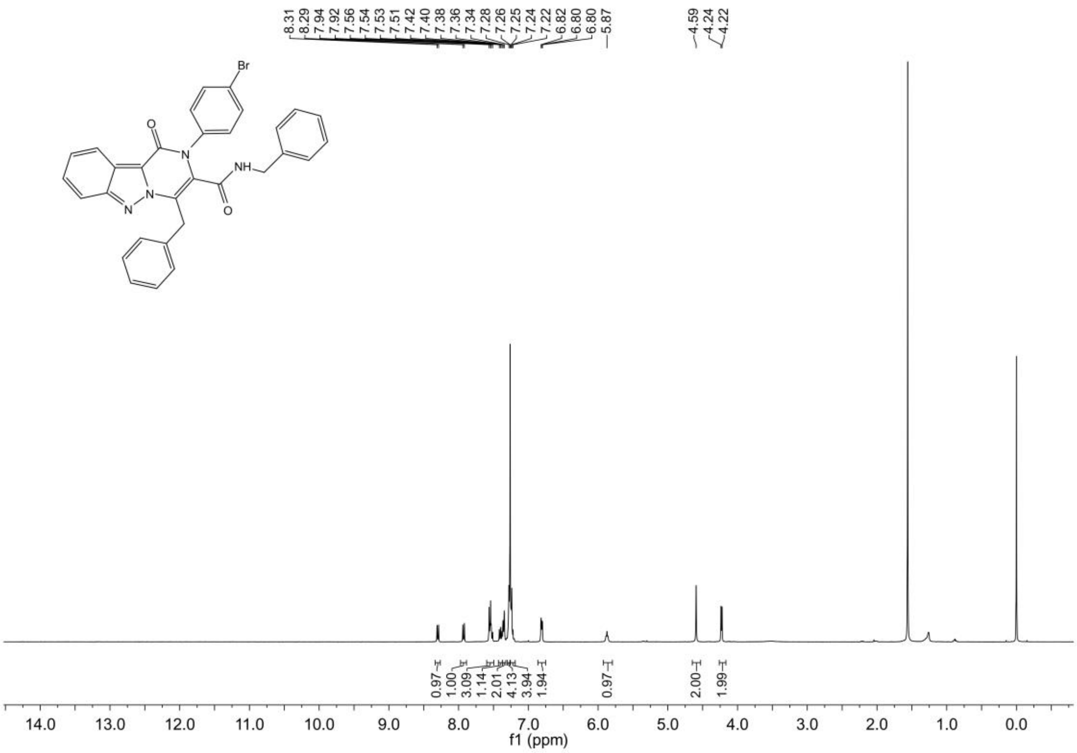
8.31
8.29
7.94
7.92
7.56
7.54
7.53
7.51
7.42
7.40
7.38
7.36
7.34
7.28
7.26
7.25
7.24
7.22
6.82
6.80
6.80
5.87
4.59
4.24
4.22
Br
O
N
NH
N
N
O
0.97
1.00
3.09
1.14
2.01
4.13
3.94
1.94
0.97
2.00
1.99
14.0
13.0
12.0
11.0
10.0
9.0
8.0
7.0
6.0
5.0
4.0
3.0
2.0
1.0
0.0
f1 (ppm)

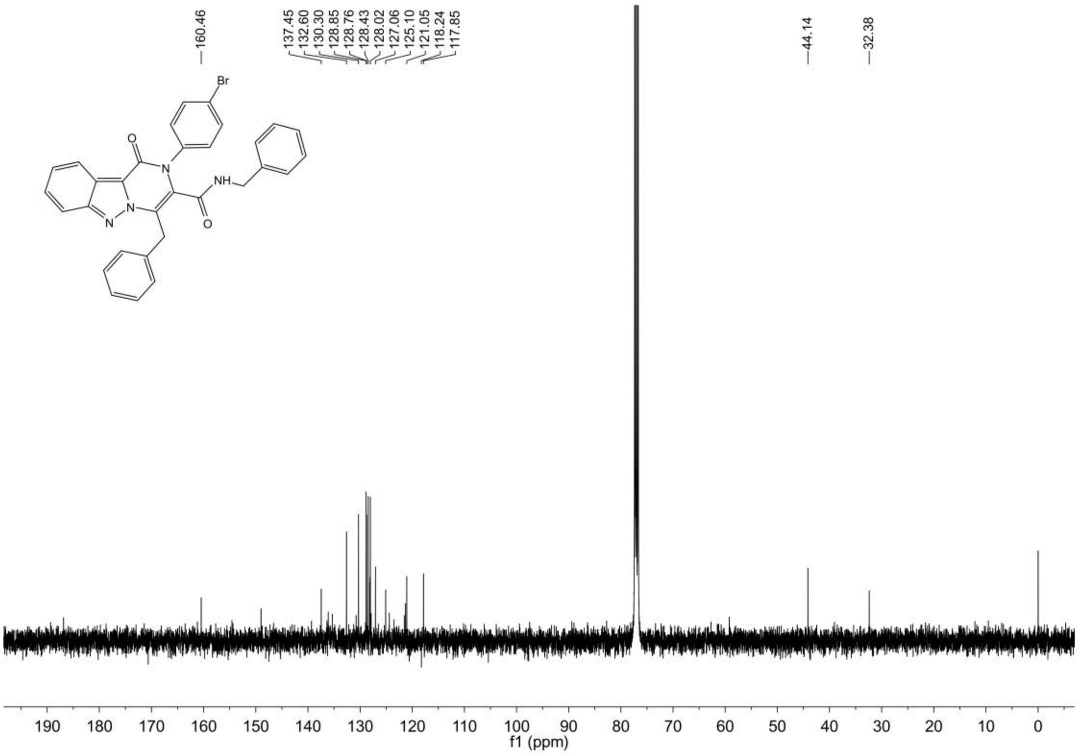
160.46
137.45
132.60
130.30
128.85
128.76
128.43
128.02
127.06
125.10
121.05
118.24
117.85
44.14
32.38
Br
O
N
NH
N
N
O
190
180
170
160
150
140
130
120
110
100
90
80
70
60
50
40
30
20
10
0
f1 (ppm)

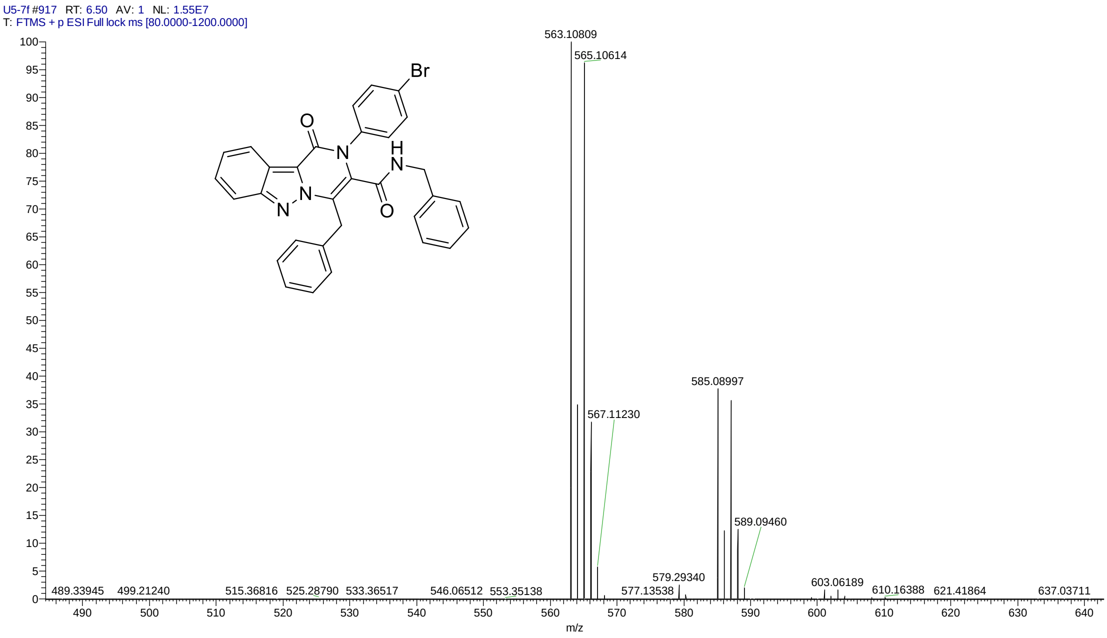
U5-7f #917 RT: 6.50 AV: 1 NL: 1.55E7
T: FTMS + p ESI Full lock ms [80.0000-1200.0000]
563.10809
565.10614
567.11230
585.08997
589.09460
579.29340
577.13538
603.06189
610.16388
621.41864
637.03711
489.33945
499.21240
515.36816
525.28790
533.36517
546.06512
553.35138
m/z
Br
O
N
H
N
N
N
O

Compound **7g**

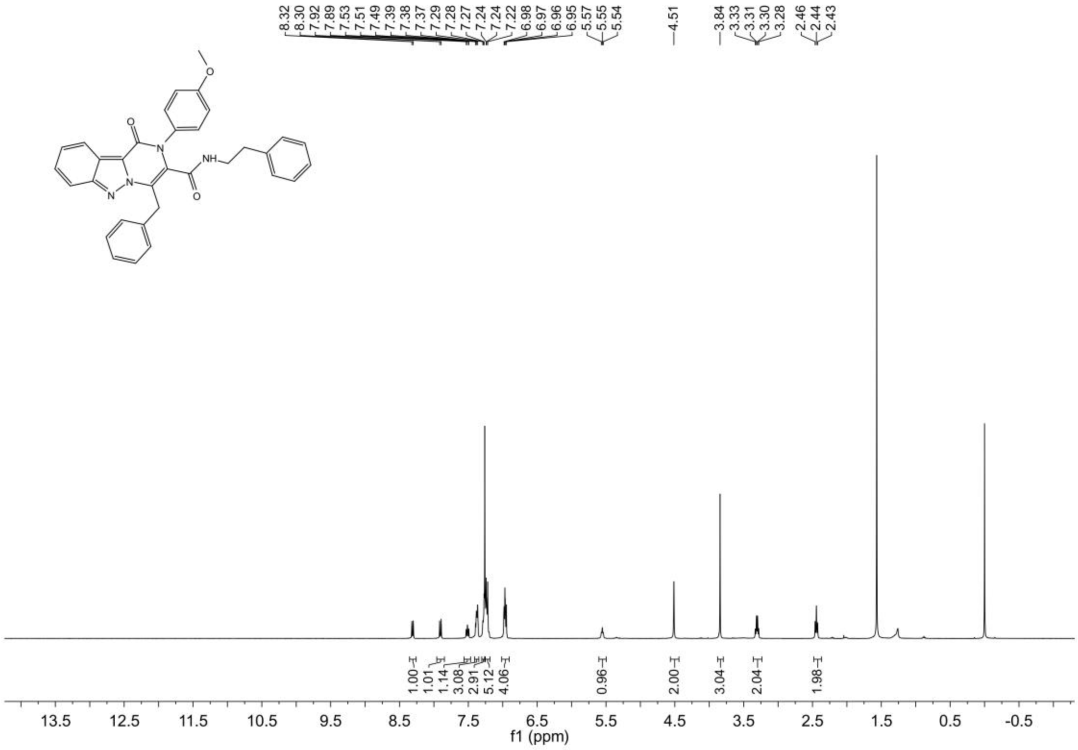
8.32
8.30
7.92
7.89
7.53
7.51
7.49
7.39
7.38
7.37
7.29
7.28
7.27
7.24
7.24
7.22
6.98
6.97
6.96
6.95
5.57
5.55
5.54
4.51
3.84
3.33
3.31
3.30
3.28
2.46
2.44
2.43
1.00
1.01
1.14
3.08
2.91
5.12
4.06
0.96
2.00
3.04
2.04
1.98
f1 (ppm)

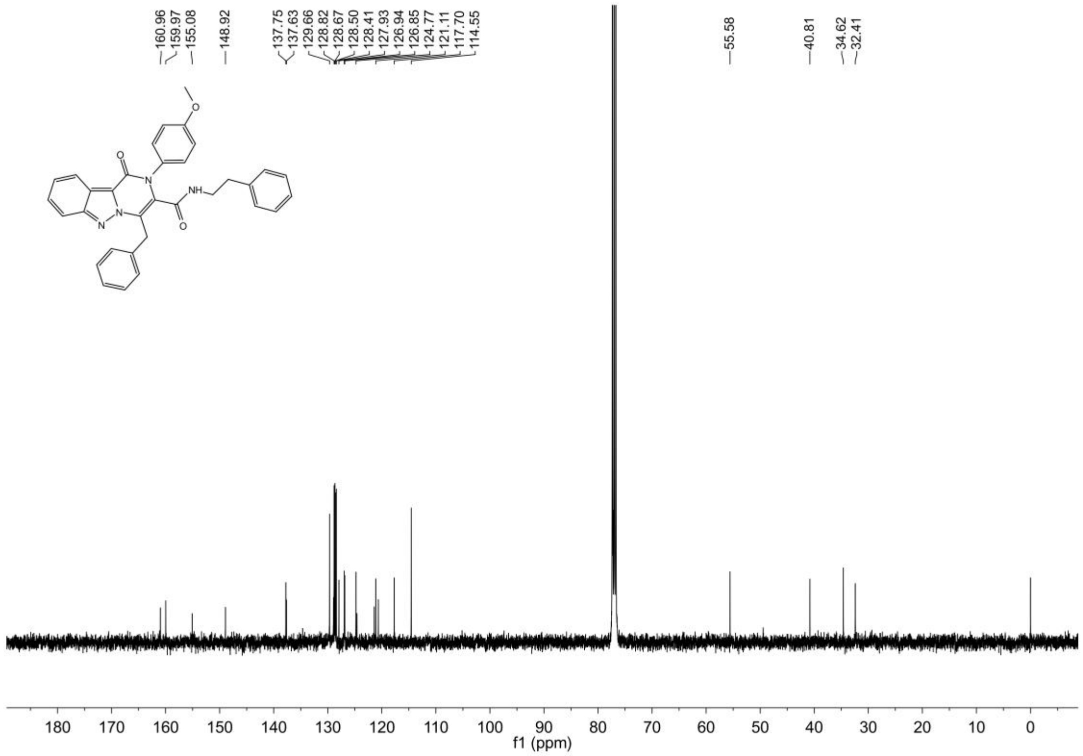
160.96
159.97
155.08
148.92
137.75
137.63
129.66
128.82
128.67
128.50
128.41
127.93
126.94
126.85
124.77
121.11
117.70
114.55
55.58
40.81
34.62
32.41
180 170 160 150 140 130 120 110 100 90 80 70 60 50 40 30 20 10 0
f1 (ppm)

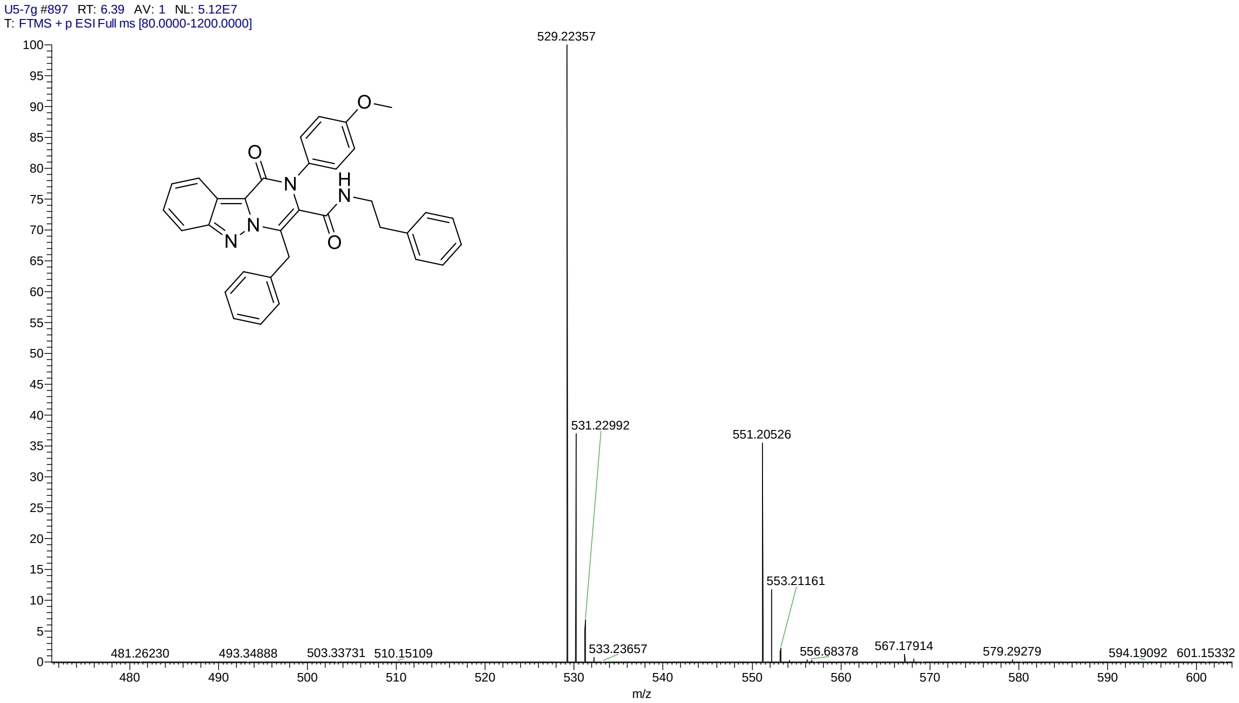
U5-7g #897 RT: 6.39 AV: 1 NL: 5.12E7
T: FTMS + p ESI Full ms [80.0000-1200.0000]
529.22357
531.22992
551.20526
553.21161
533.23657
481.26230
493.34888
503.33731
510.15109
556.68378
567.17914
579.29279
594.19092
601.15332
m/z

Compound **7h**

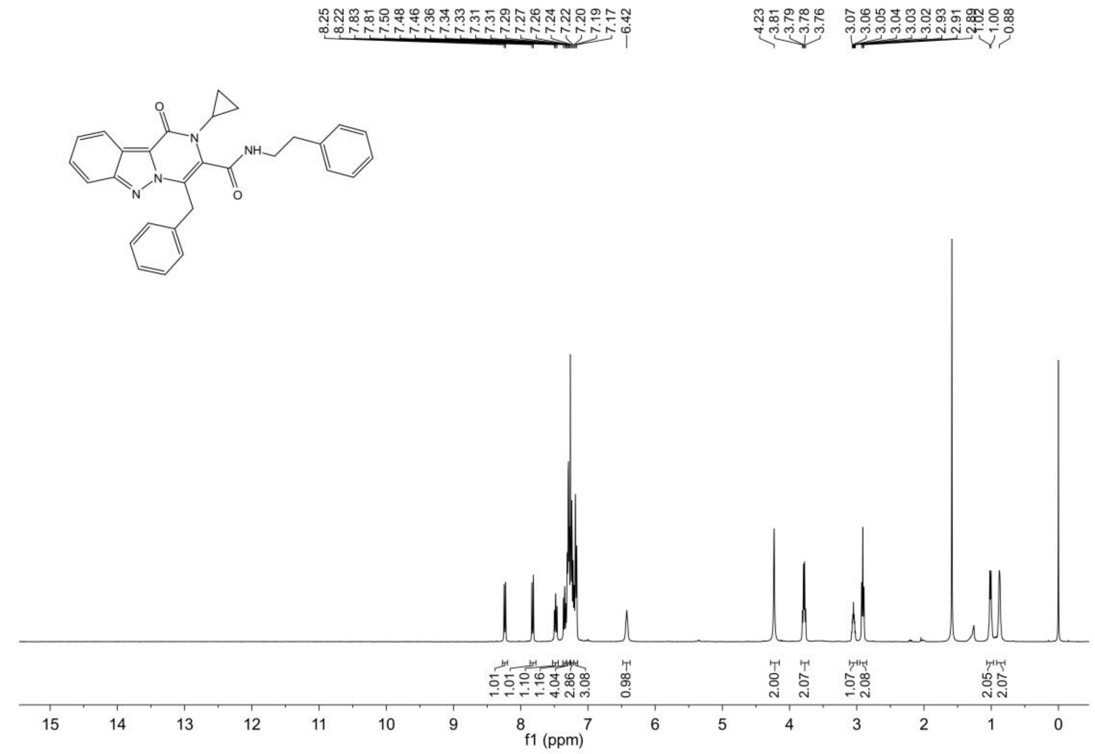
8.25
8.22
7.83
7.81
7.50
7.48
7.46
7.36
7.34
7.33
7.31
7.31
7.29
7.27
7.26
7.24
7.22
7.20
7.19
7.17
6.42
4.23
3.81
3.79
3.78
3.76
3.07
3.06
3.05
3.04
3.03
3.02
2.93
2.91
2.89
1.02
1.00
0.88
1.01
1.01
1.10
1.16
4.04
2.86
3.08
0.98
2.00
2.07
1.07
2.08
2.05
2.07
f1 (ppm)

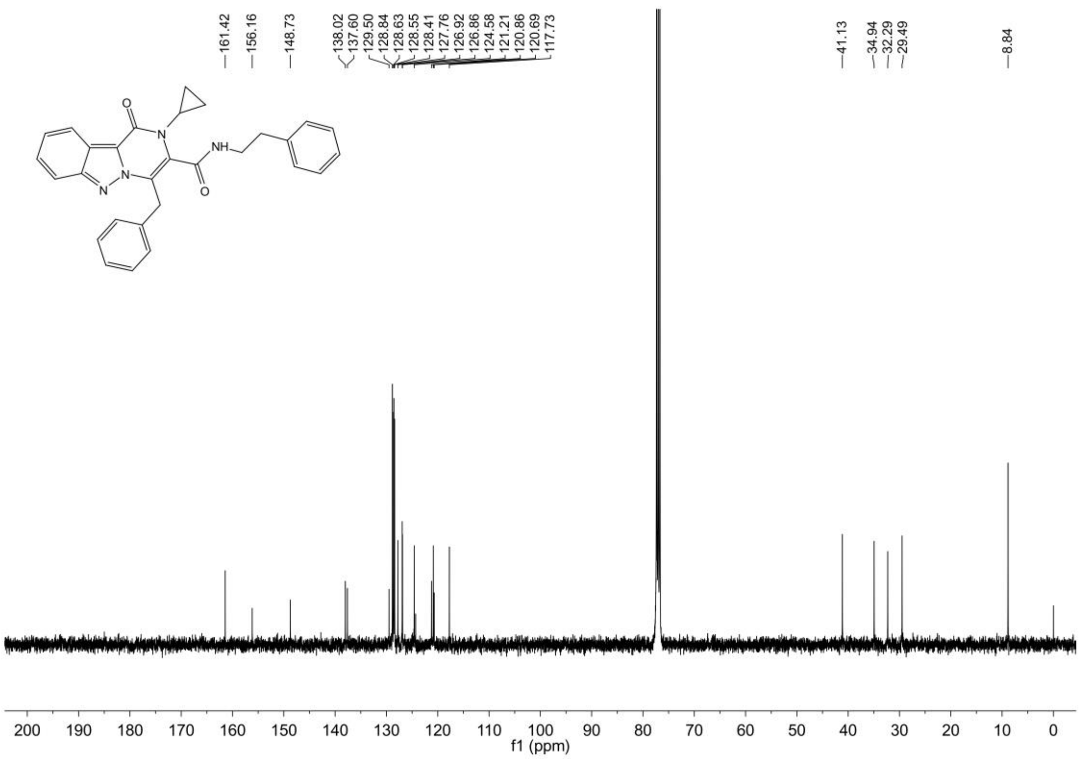
161.42
156.16
148.73
138.02
137.60
129.50
128.84
128.63
128.55
128.41
127.76
126.92
126.86
124.58
121.21
120.86
120.69
117.73
41.13
34.94
32.29
29.49
8.84
f1 (ppm)

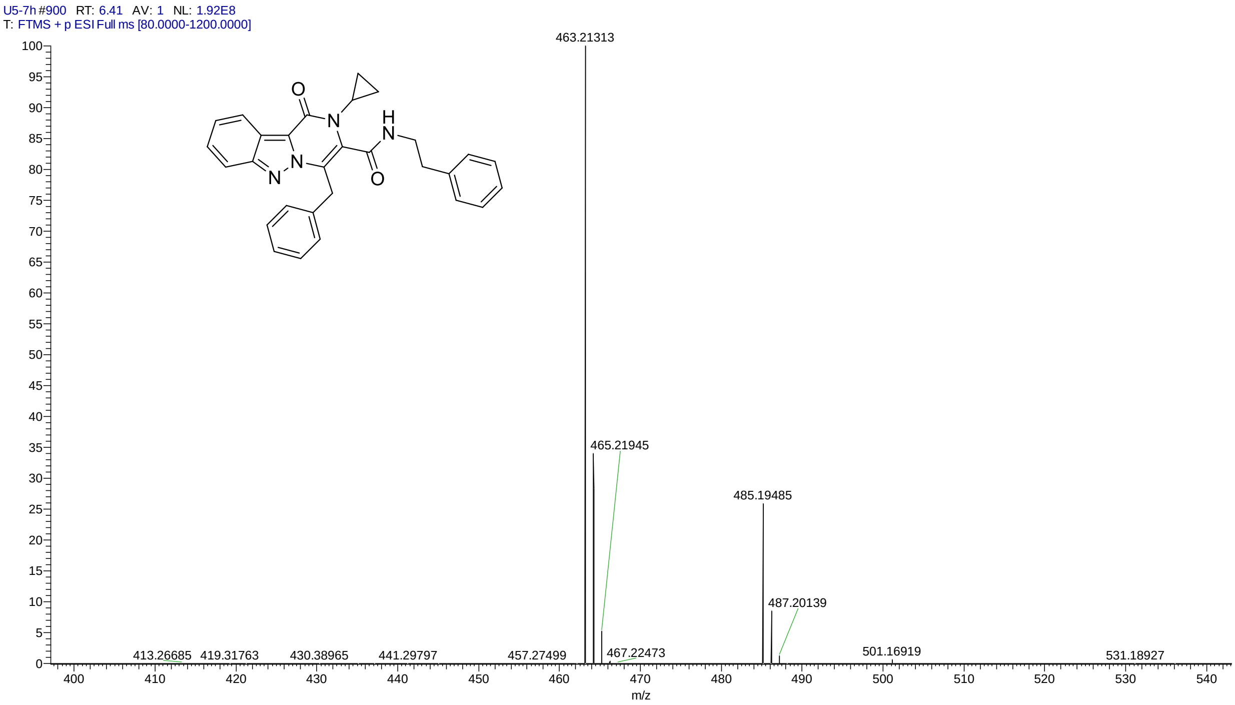
U5-7h #900 RT: 6.41 AV: 1 NL: 1.92E8
T: FTMS + p ESI Full ms [80.0000-1200.0000]
463.21313
465.21945
485.19485
487.20139
413.26685 419.31763 430.38965 441.29797 457.27499 467.22473 501.16919 531.18927
m/z

Compound **7i**

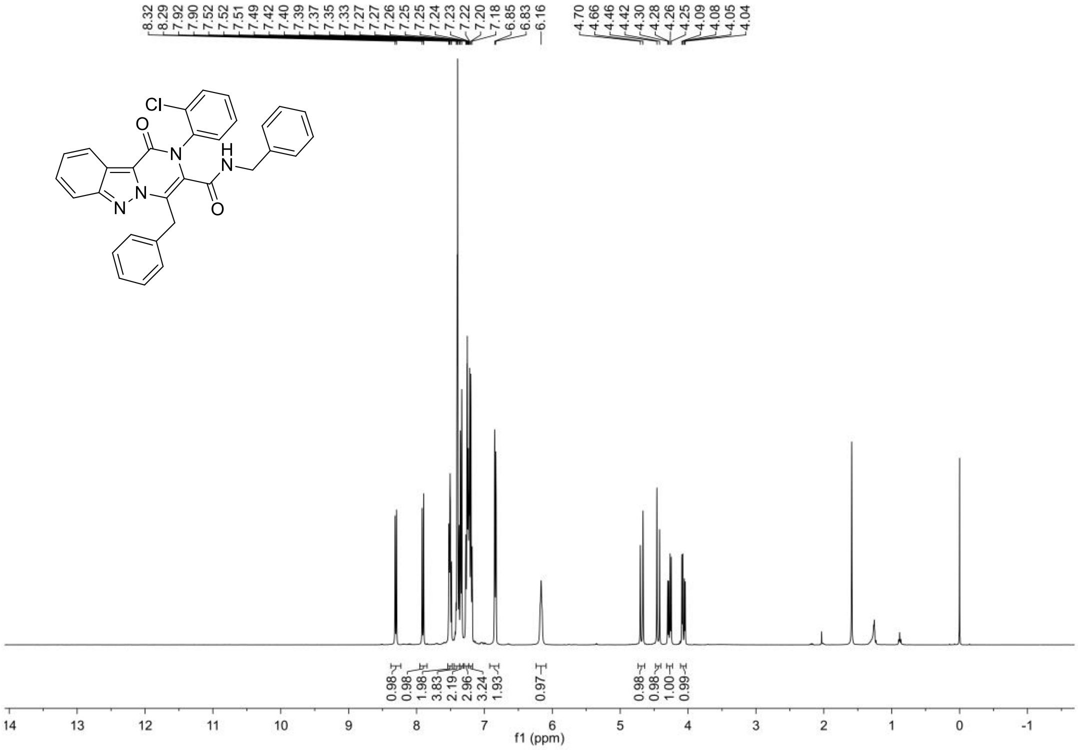
8.32 8.29 7.92 7.90 7.52 7.52 7.51 7.49 7.42 7.40 7.39 7.37 7.35 7.33 7.27 7.27 7.26 7.25 7.25 7.24 7.23 7.22 7.20 7.18 6.85 6.83 6.16
4.70 4.66 4.46 4.42 4.30 4.28 4.26 4.25 4.09 4.08 4.05 4.04
0.98 0.98 1.98 3.83 2.19 2.96 3.24 1.93 0.97 0.98 0.98 1.00 0.99
f1 (ppm)

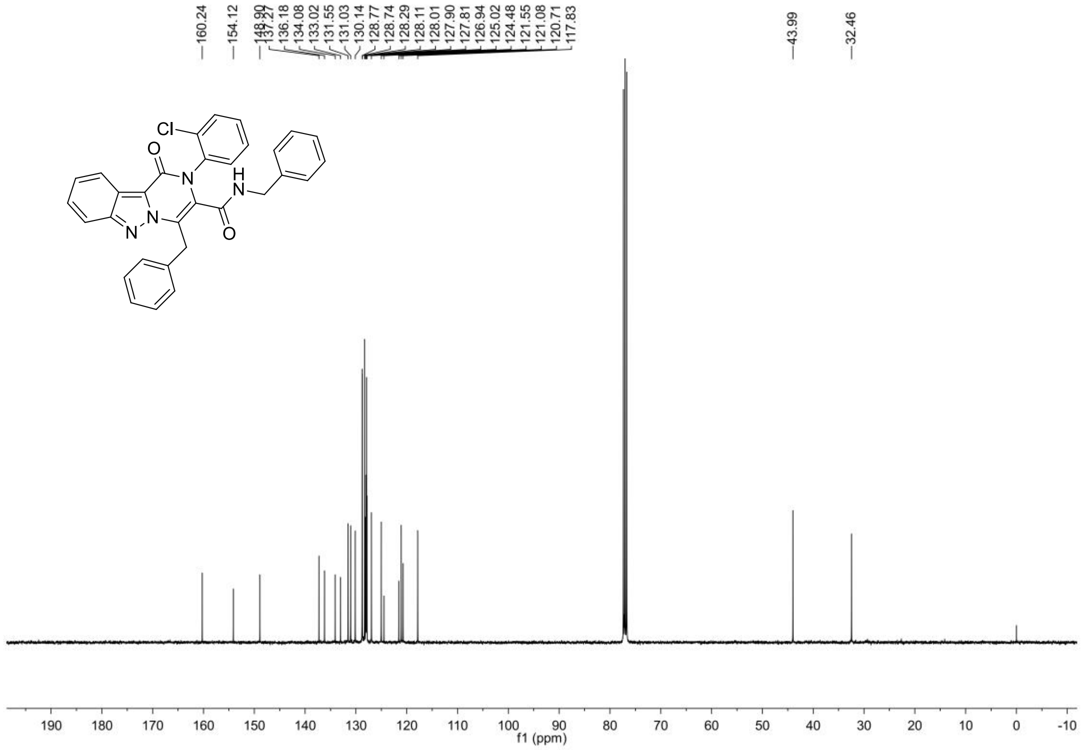

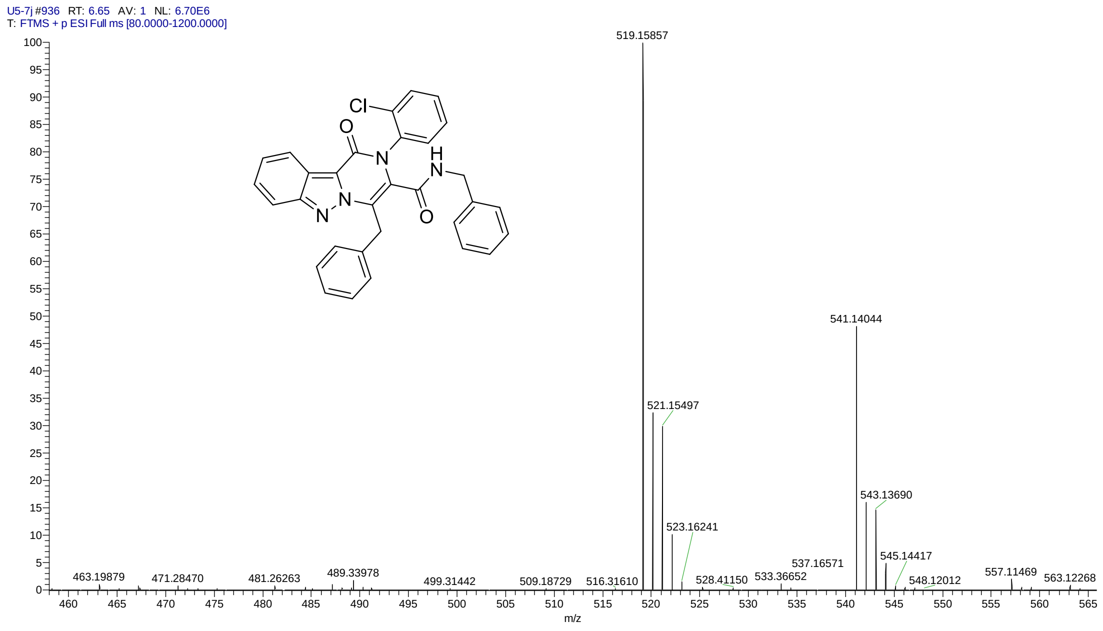

Compound **7j**

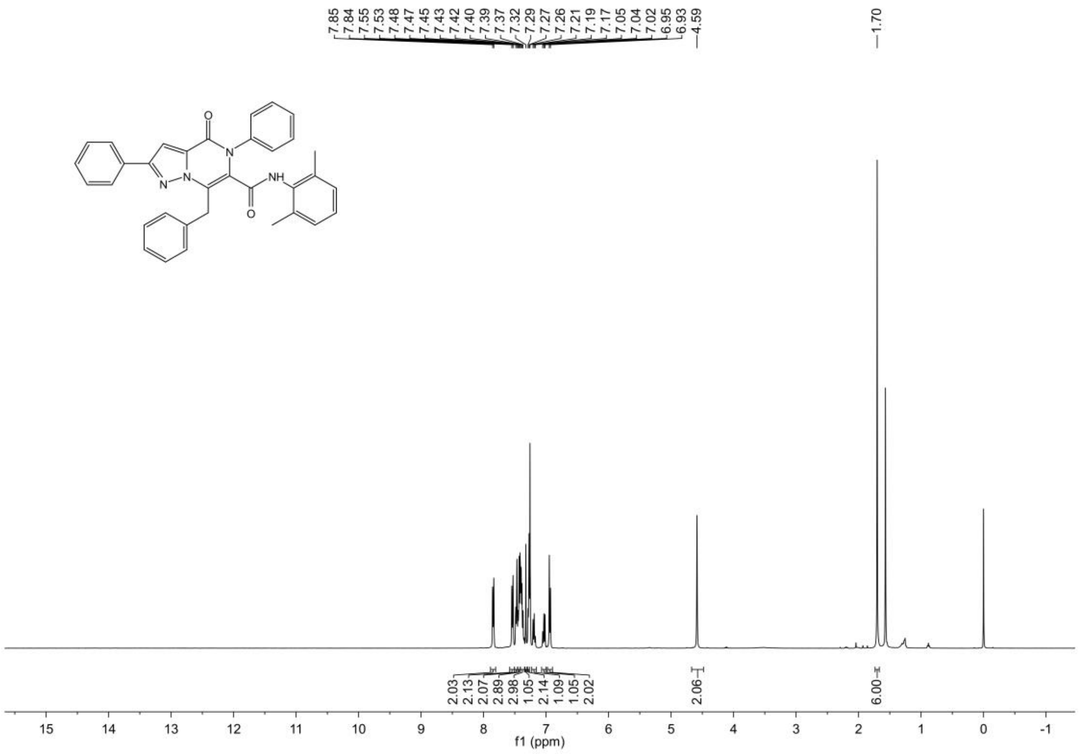
7.85
7.84
7.55
7.53
7.48
7.47
7.45
7.43
7.42
7.40
7.39
7.37
7.32
7.29
7.27
7.26
7.21
7.19
7.17
7.05
7.04
7.02
6.95
6.93
4.59
1.70
O
N
NH
N
O
2.03
2.13
2.07
2.89
2.98
1.05
2.14
1.09
1.05
2.02
2.06
6.00
15 14 13 12 11 10 9 8 7 6 5 4 3 2 1 0 -1
f1 (ppm)

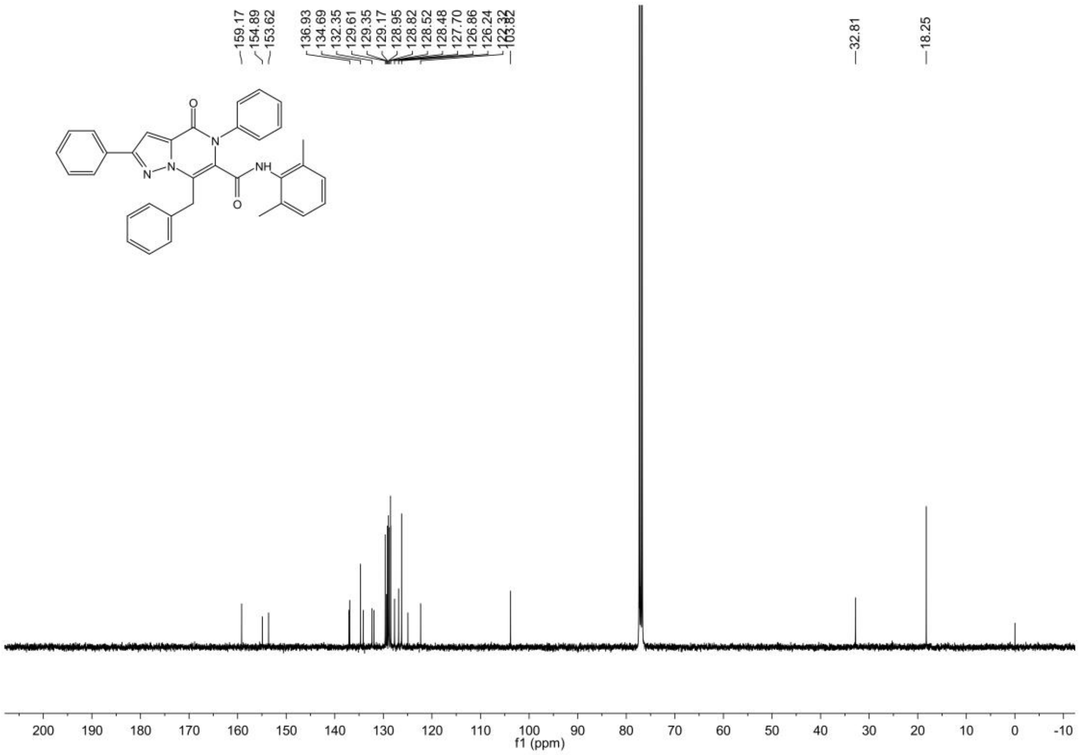
159.17
154.89
153.62
136.93
134.69
132.35
129.61
129.35
129.17
128.95
128.82
128.52
128.48
127.70
126.86
126.24
122.32
103.82
32.81
18.25
O
N
NH
N
O
200 190 180 170 160 150 140 130 120 110 100 90 80 70 60 50 40 30 20 10 0 -10
f1 (ppm)

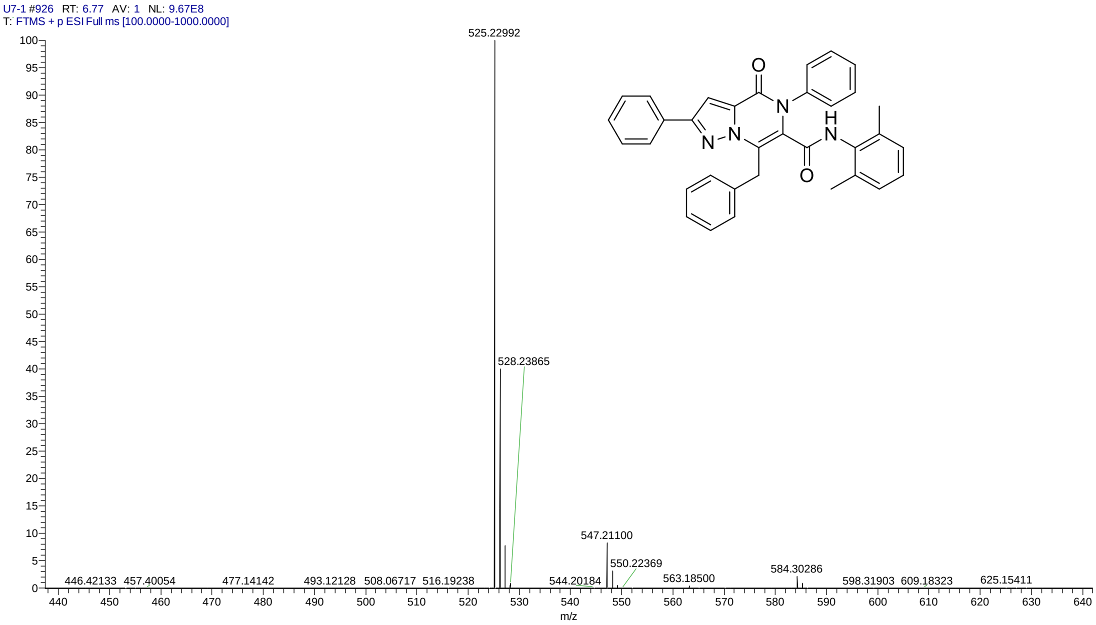
U7-1 #926 RT: 6.77 AV: 1 NL: 9.67E8
T: FTMS + p ESI Full ms [100.0000-1000.0000]
525.22992
528.23865
547.21100
550.22369
563.18500
584.30286
598.31903 609.18323
625.15411
446.42133 457.40054
477.14142
493.12128 508.06717 516.19238
544.20184
m/z

Compound **7k**

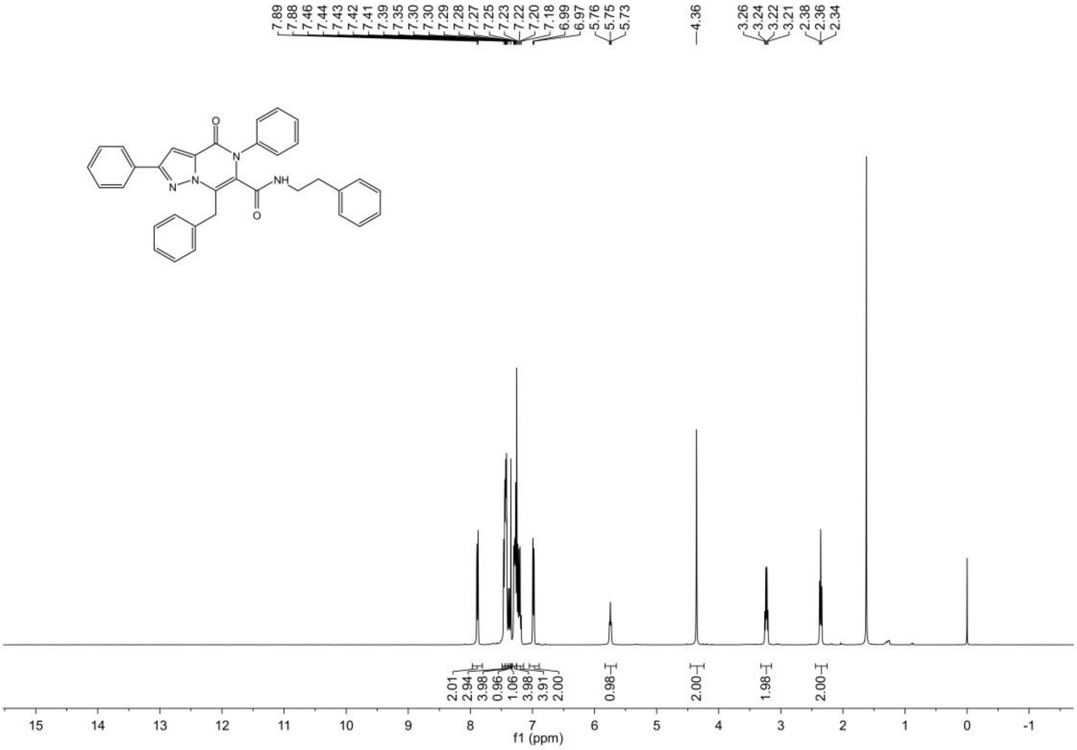
f1 (ppm)

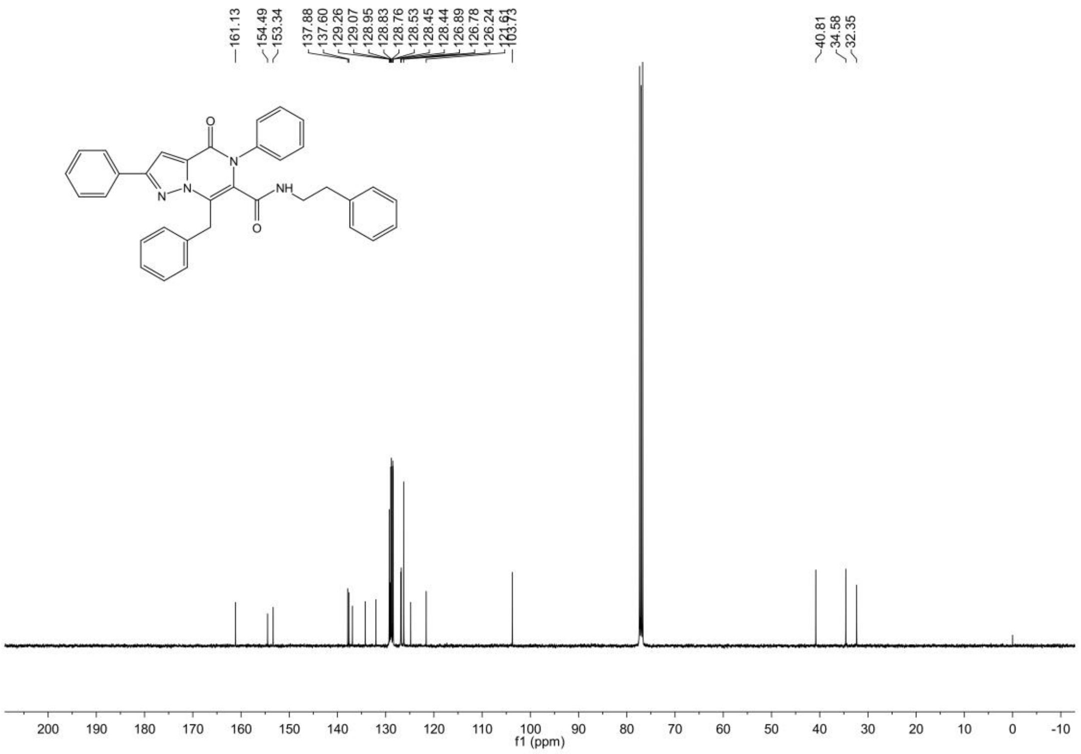
161.13
154.49
153.34
137.88
137.60
129.26
129.07
128.95
128.83
128.76
128.53
128.45
128.44
126.89
126.78
126.24
121.61
103.73
40.81
34.58
32.35
f1 (ppm)

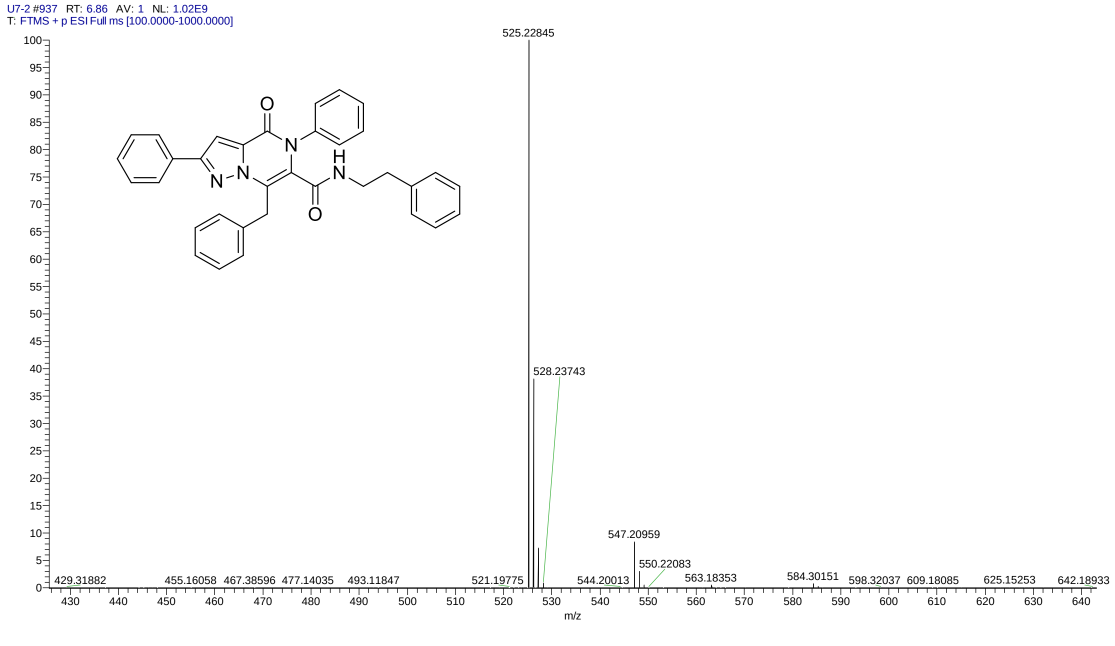
U7-2 #937 RT: 6.86 AV: 1 NL: 1.02E9
T: FTMS + p ESI Full ms [100.0000-1000.0000]
525.22845
528.23743
547.20959
550.22083
429.31882
455.16058
467.38596
477.14035
493.11847
521.19775
544.20013
563.18353
584.30151
598.32037
609.18085
625.15253
642.18933
m/z

Compound **7l**

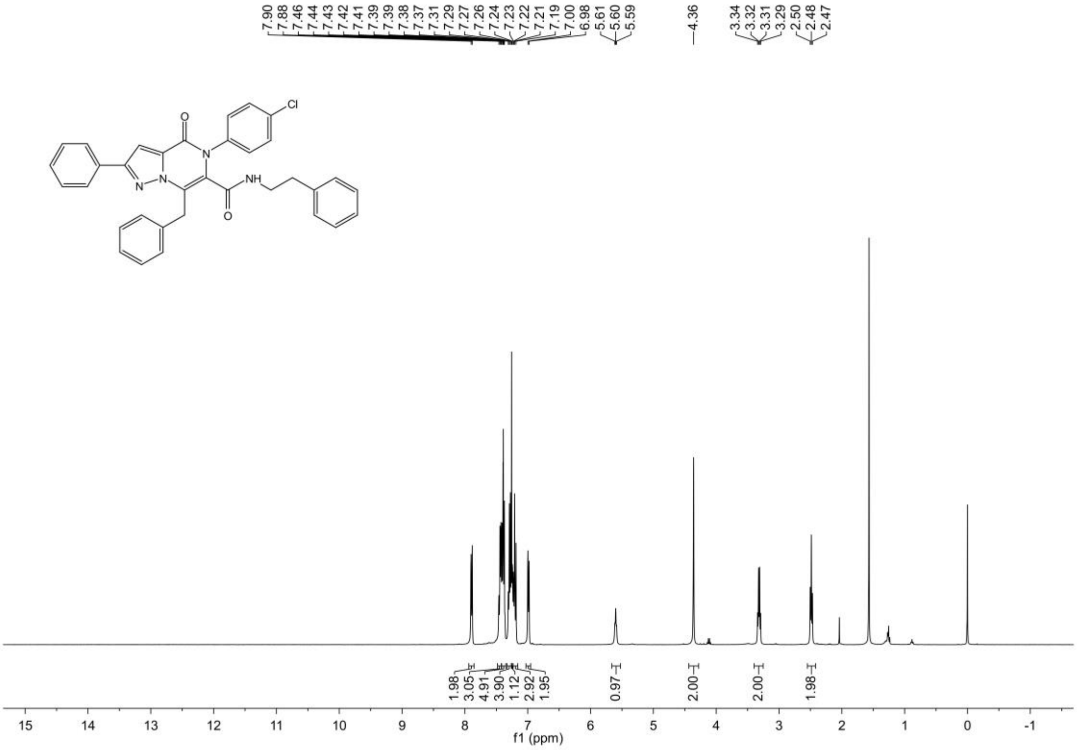

7.90
7.88
7.46
7.44
7.43
7.42
7.41
7.39
7.39
7.38
7.37
7.31
7.29
7.27
7.26
7.24
7.23
7.22
7.21
7.19
7.00
6.98
5.61
5.60
5.59
4.36
3.34
3.32
3.31
3.29
2.50
2.48
2.47
O
Cl
N
N
N
NH
O
1.98
3.05
4.91
3.90
1.12
2.92
1.95
0.97
2.00
2.00
1.98
15 14 13 12 11 10 9 8 7 6 5 4 3 2 1 0 -1
f1 (ppm)

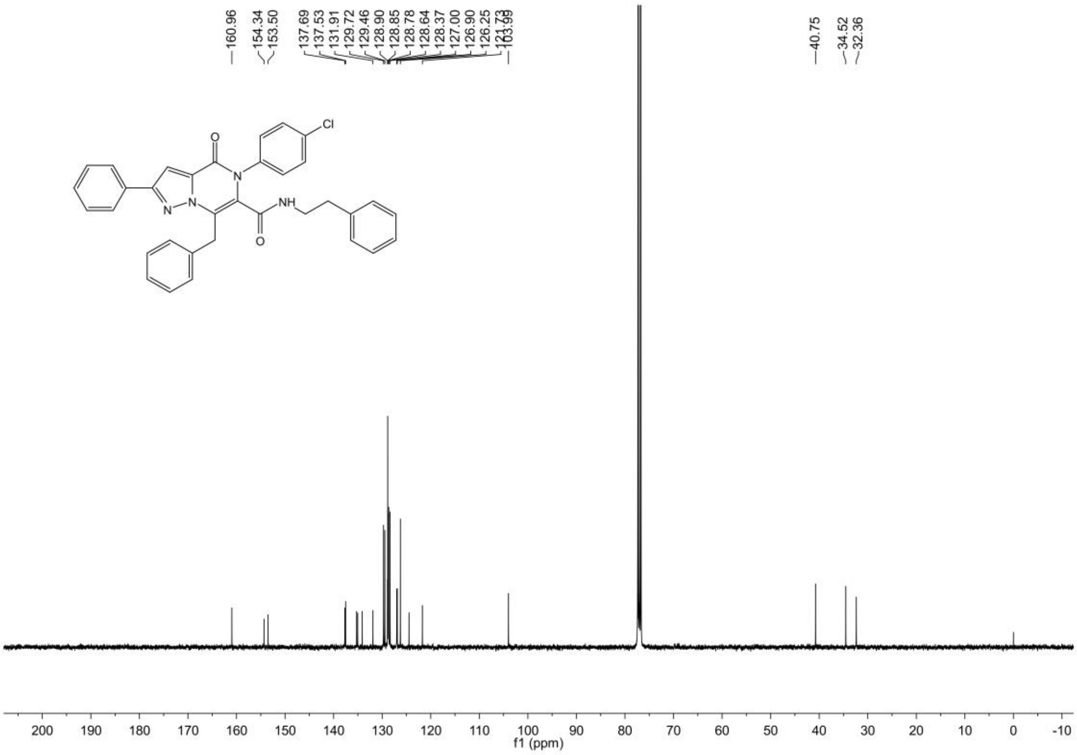

160.96
154.34
153.50
137.69
137.53
131.91
129.72
129.46
128.90
128.85
128.78
128.64
128.37
127.00
126.90
126.25
40.75
34.52
32.36
O
Cl
N
N
N
NH
O
200 190 180 170 160 150 140 130 120 110 100 90 80 70 60 50 40 30 20 10 0 -10
f1 (ppm)

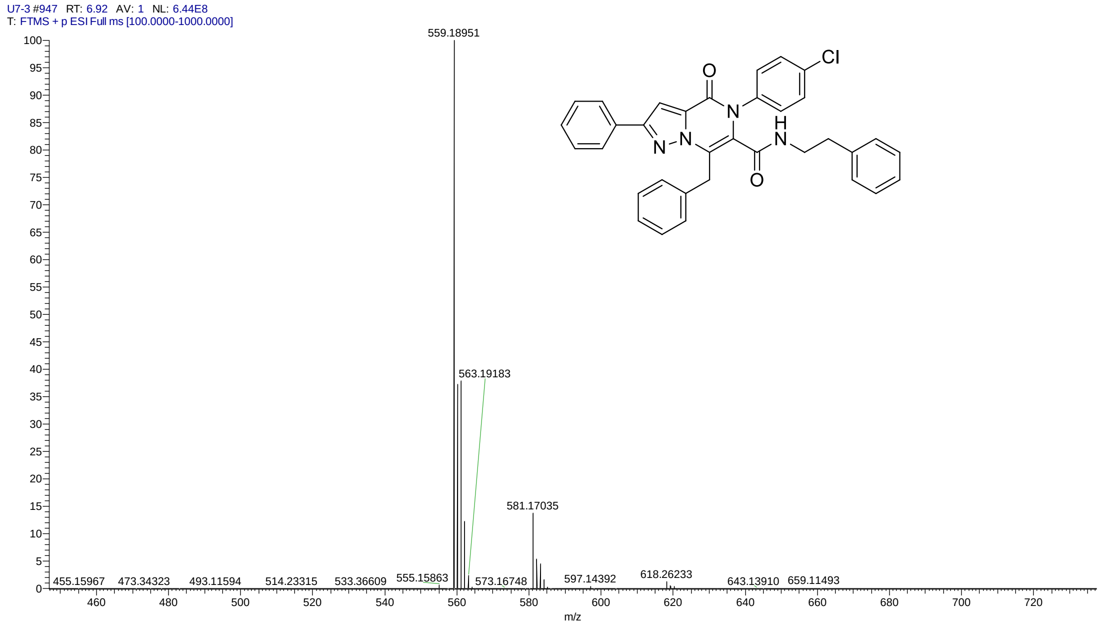
U7-3 #947 RT: 6.92 AV: 1 NL: 6.44E8
T: FTMS + p ESI Full ms [100.0000-1000.0000]
559.18951
563.19183
581.17035
455.15967
473.34323
493.11594
514.23315
533.36609
555.15863
573.16748
597.14392
618.26233
643.13910
659.11493
m/z
Cl

Compound **7m**

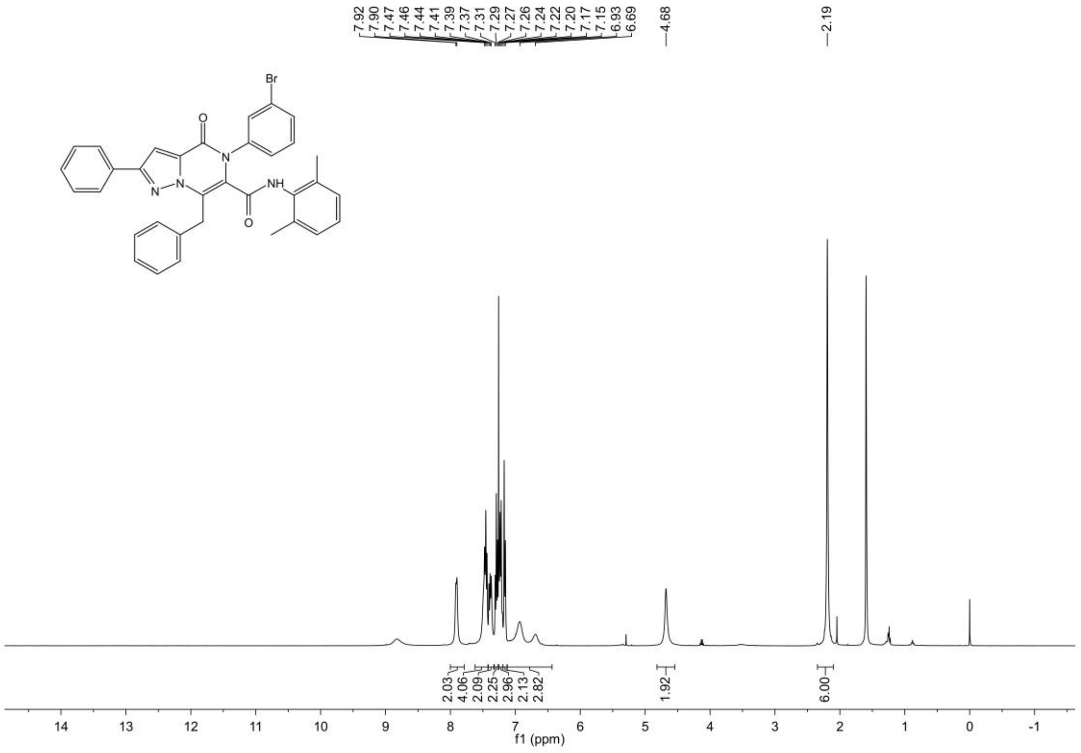
7.92
7.90
7.47
7.46
7.44
7.41
7.39
7.37
7.31
7.29
7.27
7.26
7.24
7.22
7.20
7.17
7.15
6.93
6.69
4.68
2.19
Br
NH
2.03
4.06
2.09
2.25
2.96
2.13
2.82
1.92
6.00
f1 (ppm)

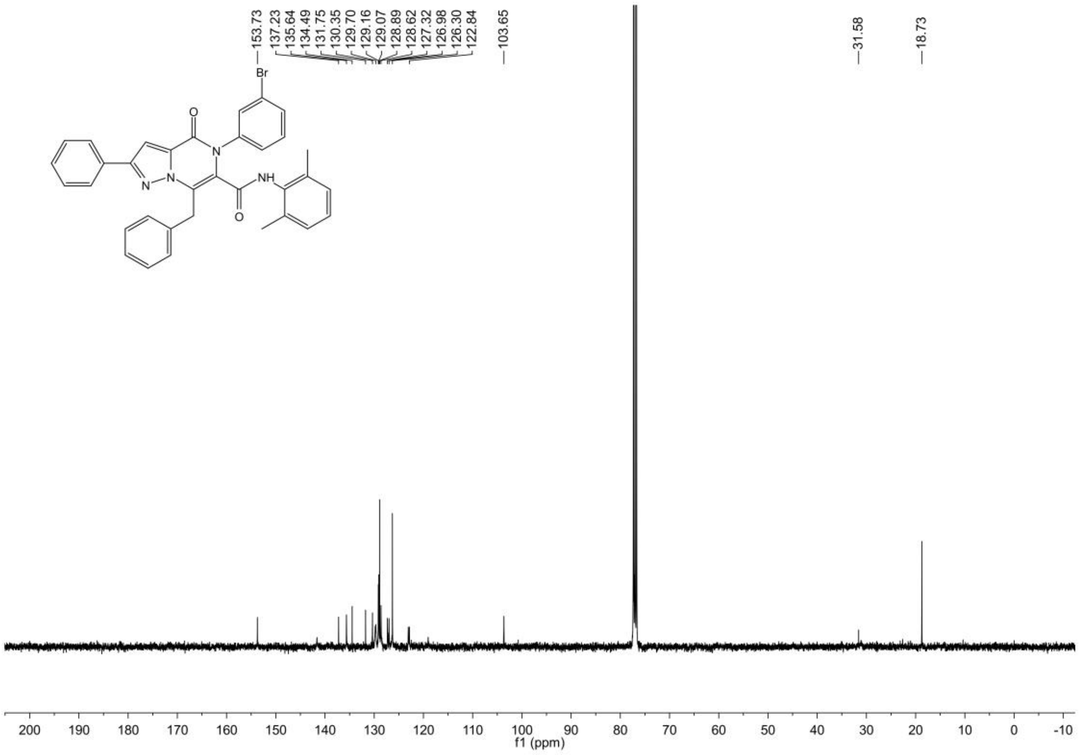
153.73
137.23
135.64
134.49
131.75
130.35
129.70
129.16
129.07
128.89
128.62
127.32
126.98
126.30
122.84
103.65
31.58
18.73
Br
O
N
N
N
NH
O
200 190 180 170 160 150 140 130 120 110 100 90 80 70 60 50 40 30 20 10 0 -10
f1 (ppm)

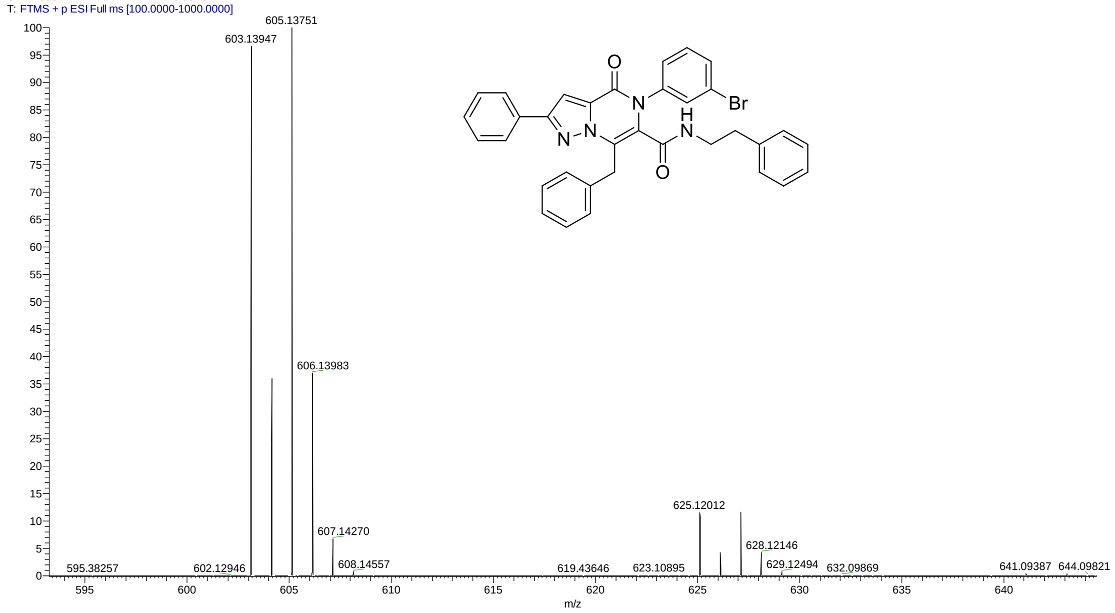
U7-4 #993 RT: 7.20 AV: 1 NL: 6.05E8
T: FTMS + p ESI Full ms [100.0000-1000.0000]
605.13751
603.13947
606.13983
607.14270
608.14557
595.38257
602.12946
619.43646
623.10895
625.12012
628.12146
629.12494
632.09869
641.09387
644.09821
m/z
O
N
Br
H
N
N
N
O

Compound **7n**

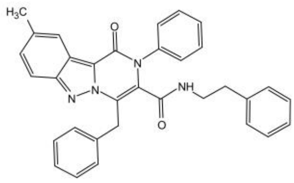
H3C
O
N
N
N
NH
O

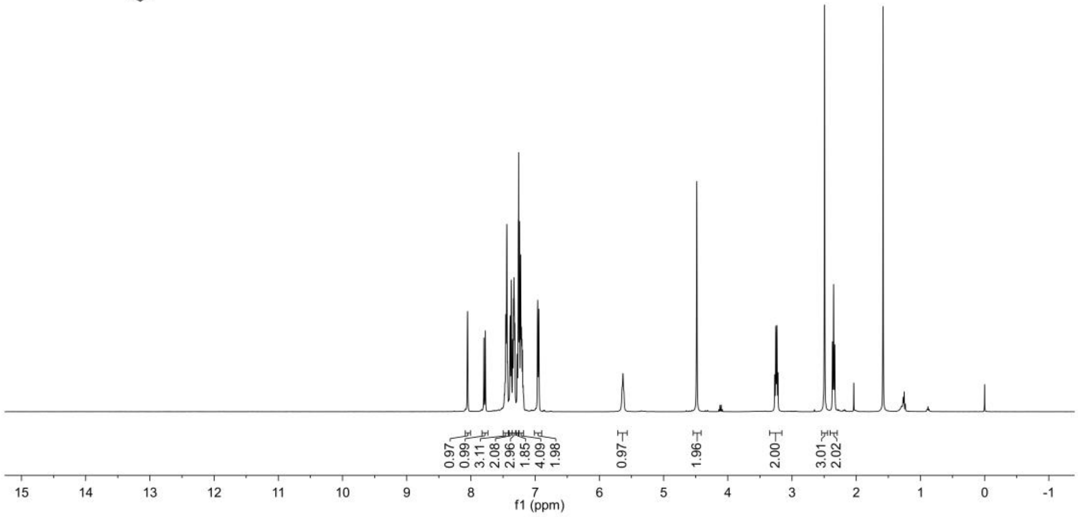
8.05
7.80
7.78
7.47
7.46
7.44
7.43
7.39
7.37
7.35
7.35
7.34
7.33
7.32
7.28
7.26
7.26
7.24
7.23
7.21
7.21
7.20
6.96
6.94
5.63
4.48
3.25
3.24
2.49
2.37
2.35
2.33
0.97
0.99
3.11
2.08
2.96
1.85
4.09
1.98
0.97
1.96
2.00
3.01
2.02
f1 (ppm)

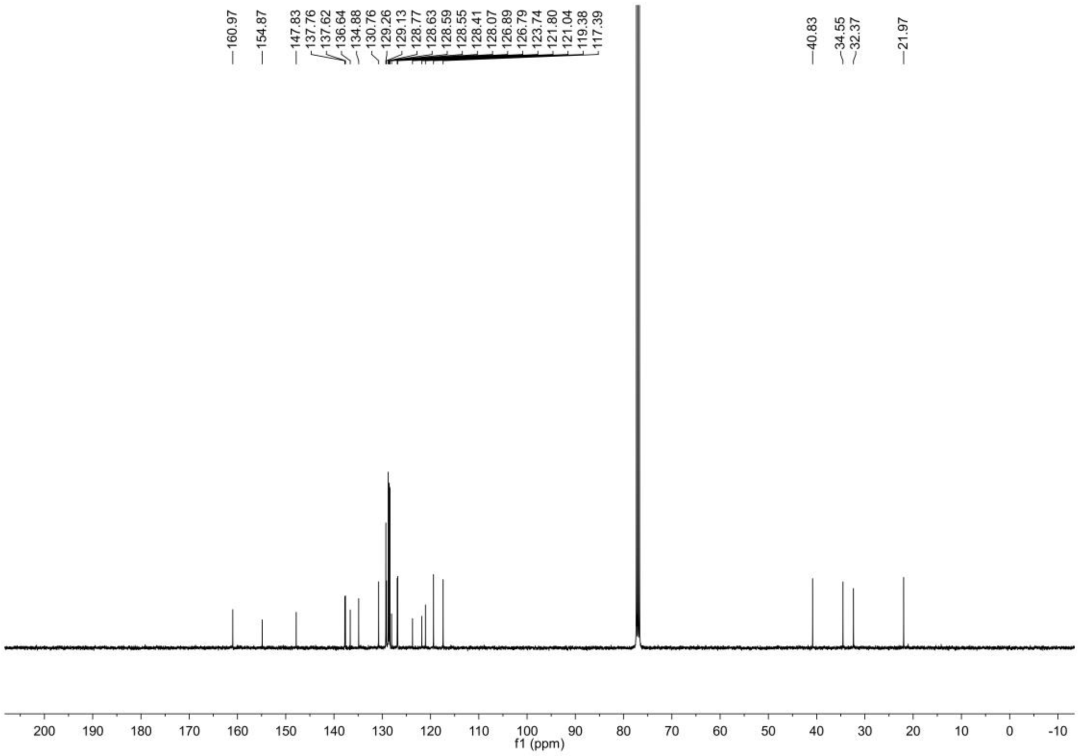
160.97
154.87
147.83
137.76
137.62
136.64
134.88
130.76
129.26
129.13
128.77
128.63
128.59
128.55
128.41
128.07
126.89
126.79
123.74
121.80
121.04
119.38
117.39
40.83
34.55
32.37
21.97
f1 (ppm)

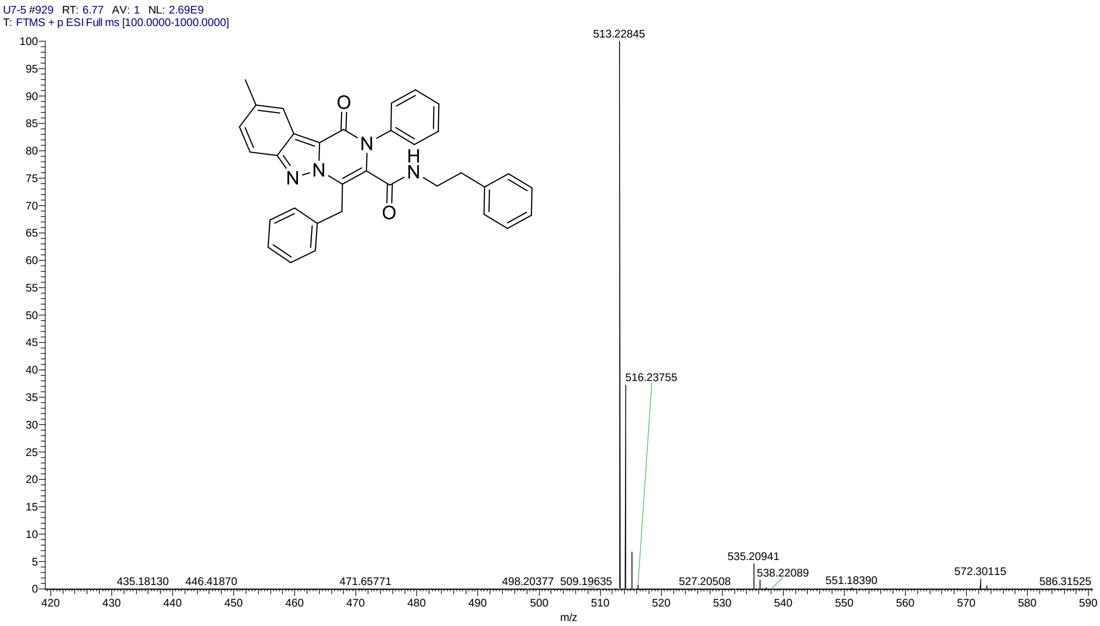
U7-5 #929 RT: 6.77 AV: 1 NL: 2.69E9
T: FTMS + p ESI Full ms [100.0000-1000.0000]
513.22845
516.23755
535.20941
538.22089
435.18130
446.41870
471.65771
498.20377
509.19635
527.20508
551.18390
572.30115
586.31525
m/z

Compound **7o**

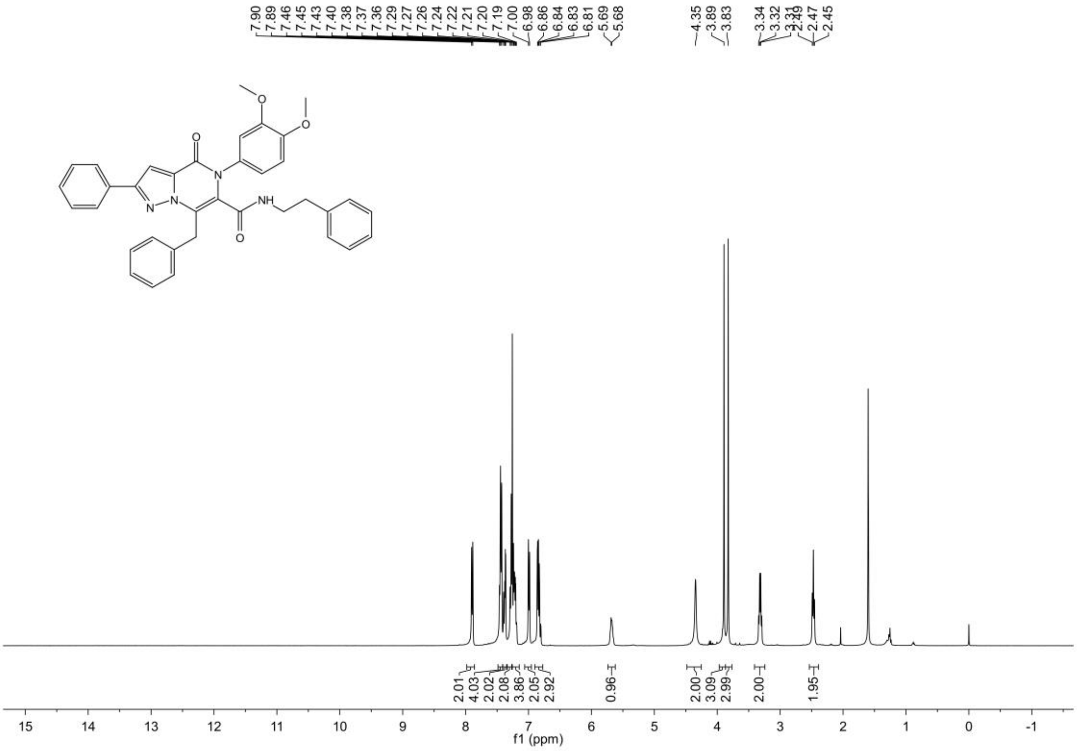
7.90 7.89 7.46 7.45 7.43 7.40 7.38 7.37 7.36 7.29 7.27 7.26 7.24 7.22 7.21 7.20 7.19 7.00 6.98 6.86 6.84 6.83 6.81 5.69 5.68 4.35 3.89 3.83 3.34 3.32 3.31 2.49 2.47 2.45
2.01 4.03 2.02 2.08 3.86 2.05 2.92 0.96 2.00 3.09 2.99 2.00 1.95
f1 (ppm)

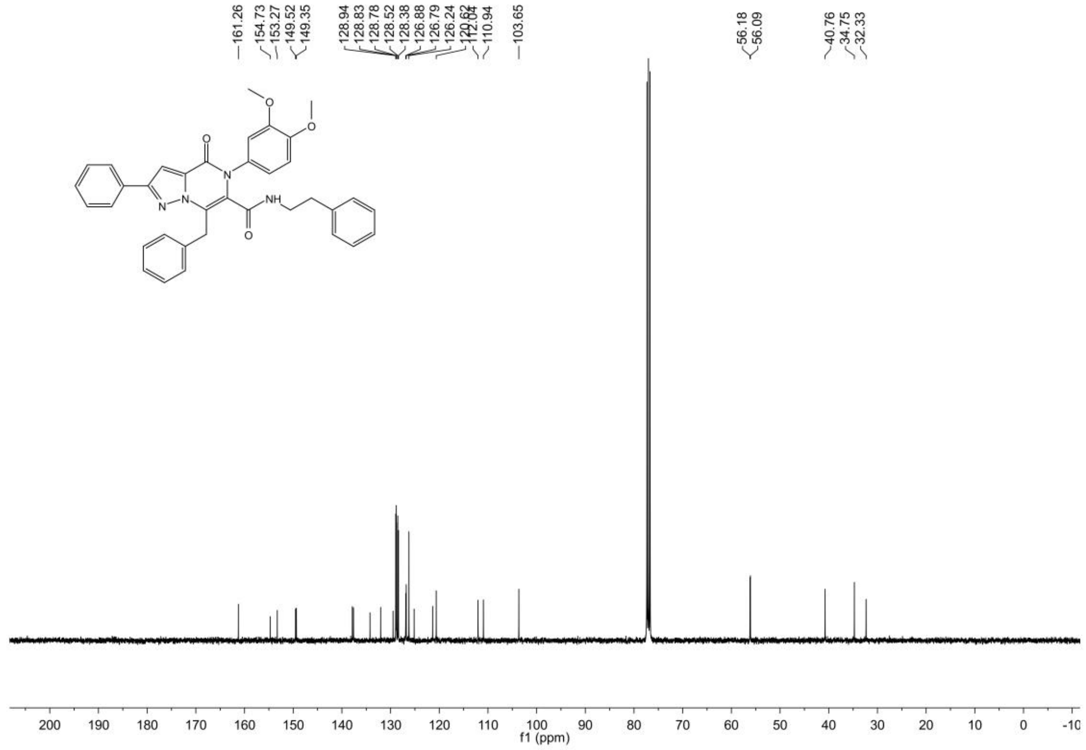
161.26
154.73
153.27
149.52
149.35
128.94
128.83
128.78
128.52
128.38
126.88
126.79
126.24
110.94
103.65
56.18
56.09
40.76
34.75
32.33
N
NH
O
f1 (ppm)

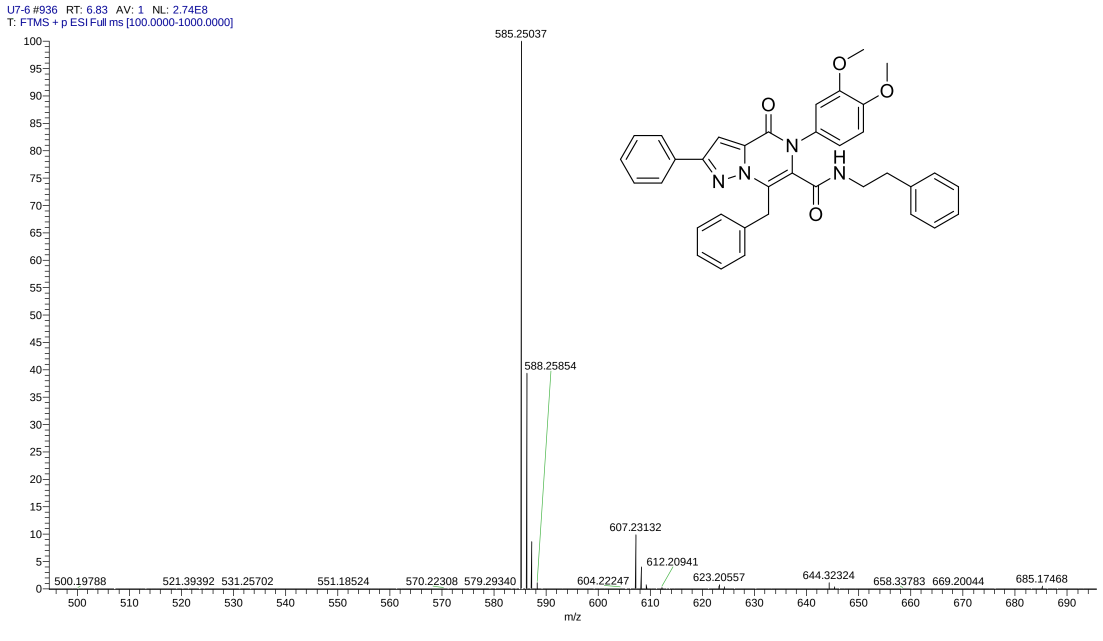
U7-6 #936 RT: 6.83 AV: 1 NL: 2.74E8
T: FTMS + p ESI Full ms [100.0000-1000.0000]
585.25037
588.25854
607.23132
612.20941
500.19788
521.39392
531.25702
551.18524
570.22308
579.29340
604.22247
623.20557
644.32324
658.33783
669.20044
685.17468
m/z

Compound **7p**

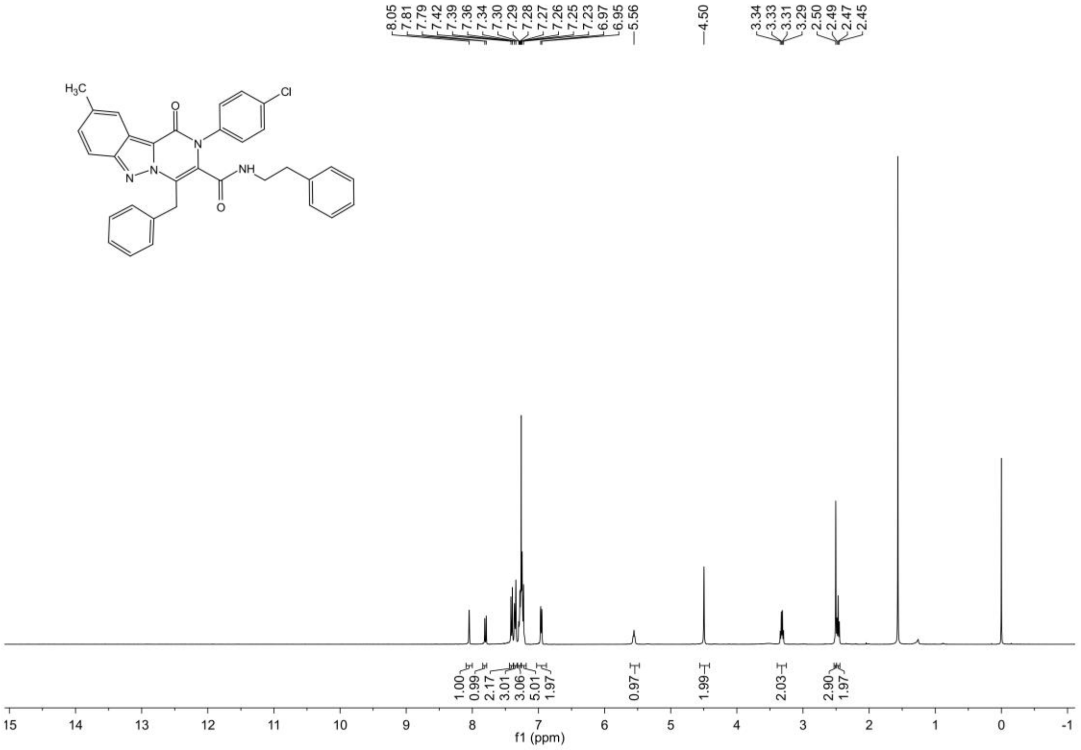
8.05
7.81
7.79
7.42
7.39
7.36
7.34
7.30
7.29
7.28
7.27
7.26
7.25
7.23
6.97
6.95
5.56
4.50
3.34
3.33
3.31
3.29
2.50
2.49
2.47
2.45
H3C
O
N
Cl
N
N
NH
O
1.00
0.99
2.17
3.01
3.06
5.01
1.97
0.97
1.99
2.03
2.90
1.97
15 14 13 12 11 10 9 8 7 6 5 4 3 2 1 0 -1
f1 (ppm)

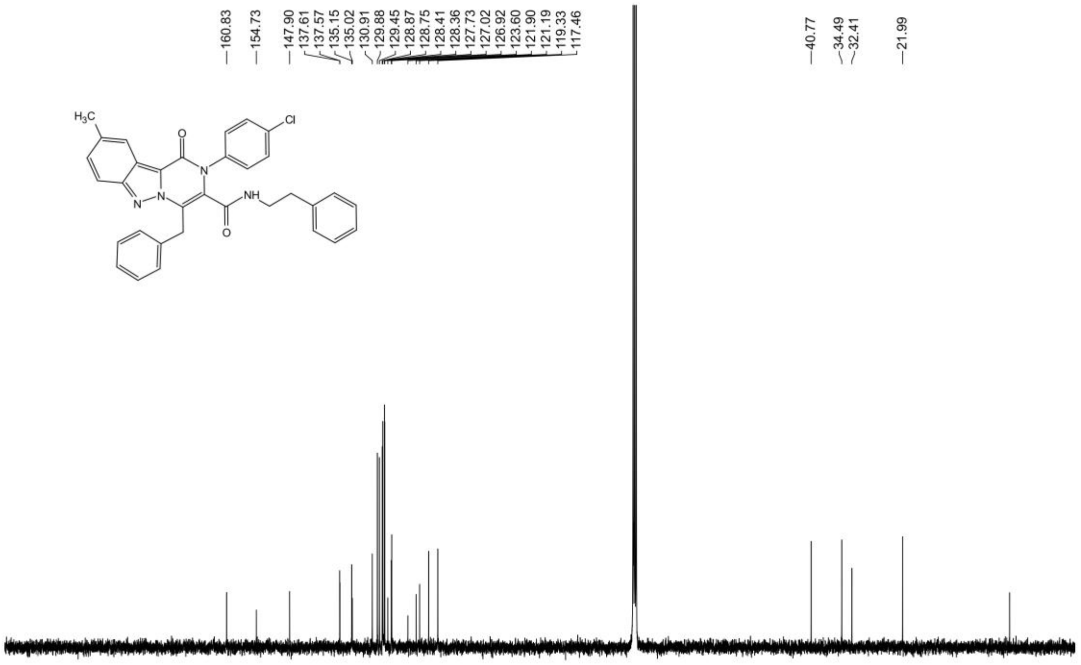
160.83
154.73
147.90
137.61
137.57
135.15
135.02
130.91
129.88
129.45
128.87
128.75
128.41
128.36
127.73
127.02
126.92
123.60
121.90
121.19
119.33
117.46
40.77
34.49
32.41
21.99
H3C
O
N
Cl
N
N
NH
O
200 190 180 170 160 150 140 130 120 110 100 90 80 70 60 50 40 30 20 10 0 -10
f1 (ppm)

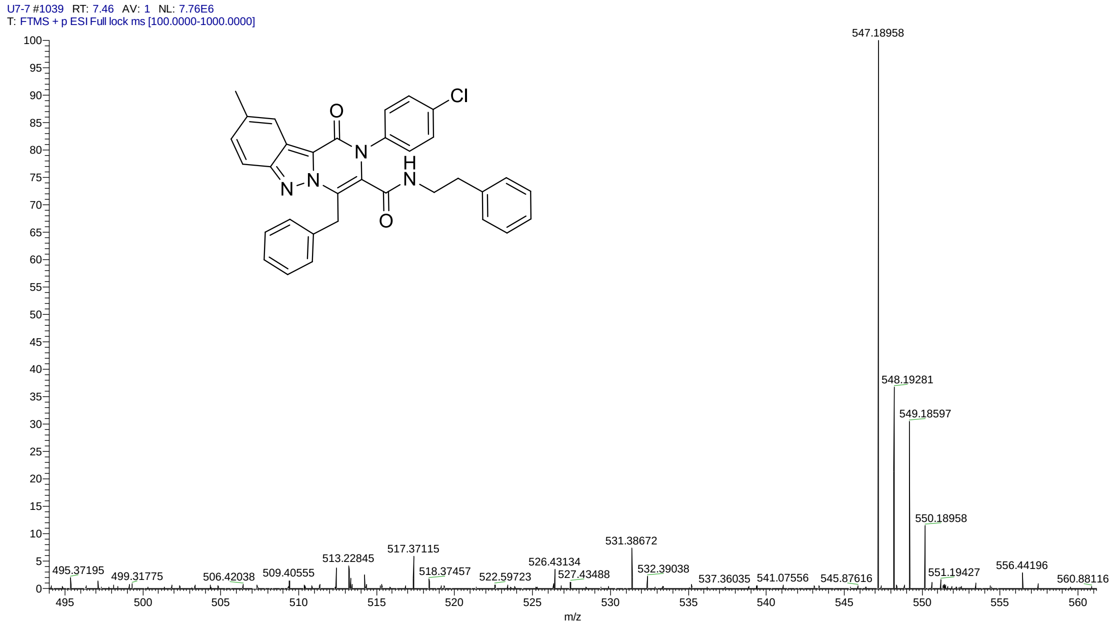
U7-7 #1039 RT: 7.46 AV: 1 NL: 7.76E6
T: FTMS + p ESI Full lock ms [100.0000-1000.0000]
547.18958
548.19281
549.18597
550.18958
531.38672
517.37115
513.22845
526.43134
m/z

Compound **7q**

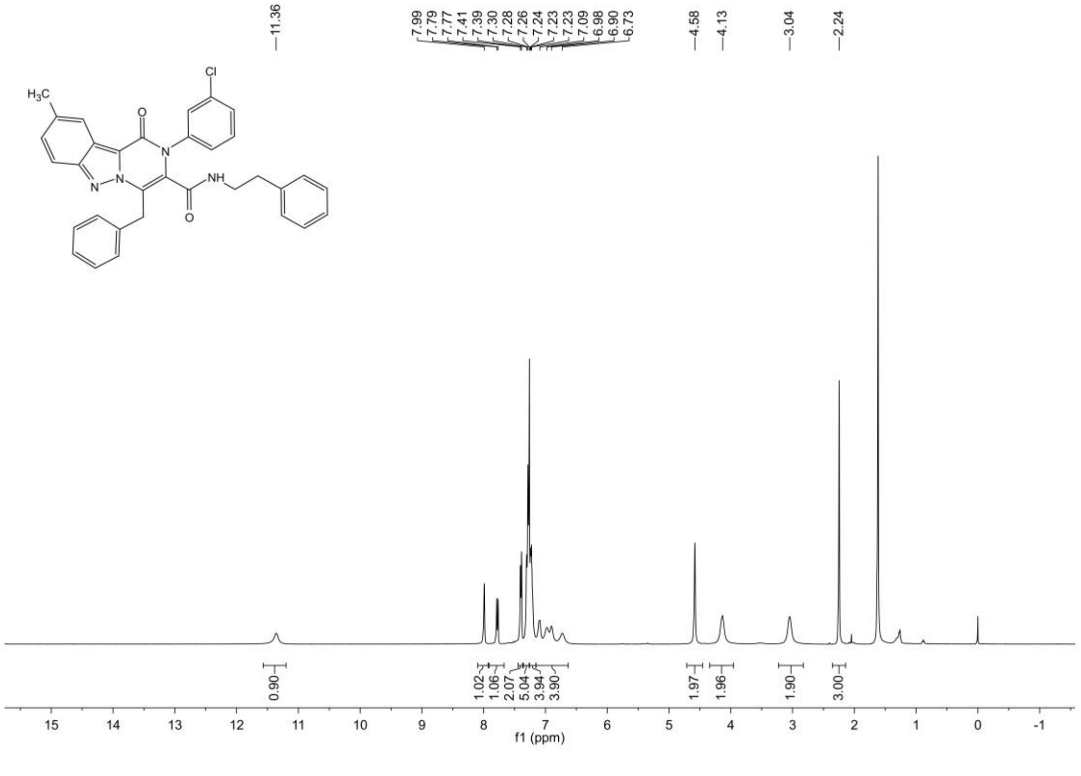
11.36
7.99 7.79 7.77 7.41 7.39 7.30 7.28 7.26 7.24 7.23 7.23 7.09 6.98 6.90 6.73
4.58
4.13
3.04
2.24
0.90 1.02 1.06 2.07 5.04 3.94 3.90 1.97 1.96 1.90 3.00
f1 (ppm)

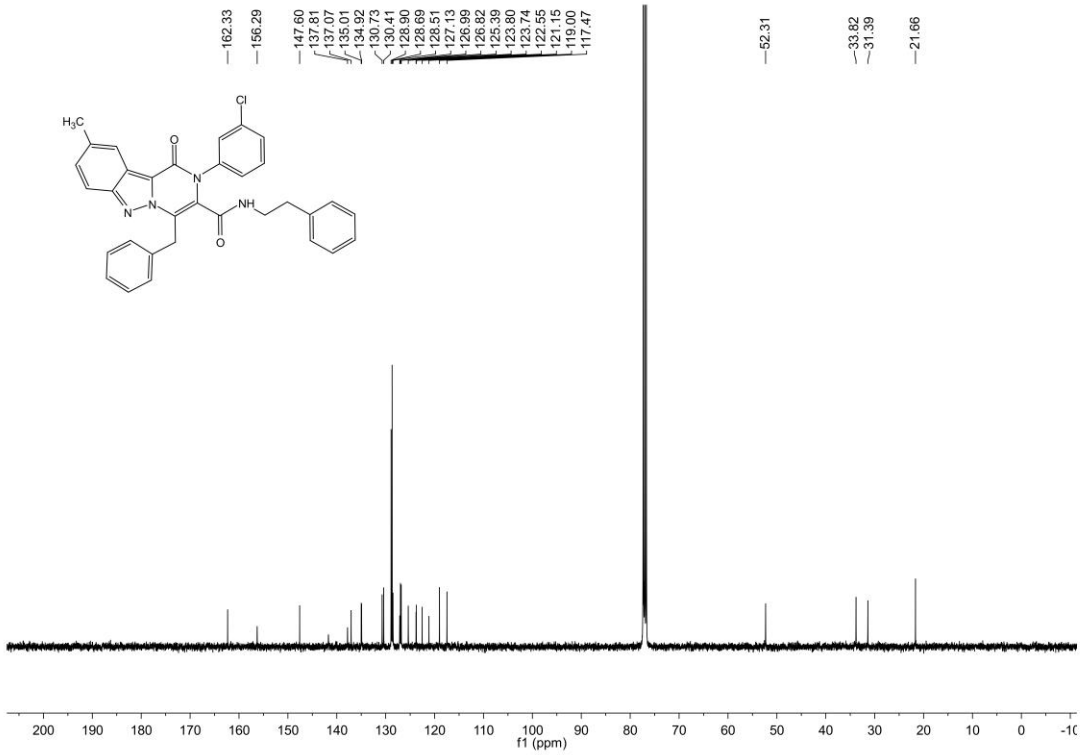

162.33
156.29
147.60
137.81
137.07
135.01
134.92
130.73
130.41
128.90
128.69
128.51
127.13
126.99
126.82
125.39
123.80
123.74
122.55
121.15
119.00
117.47
52.31
33.82
31.39
21.66
Cl
H3C
O
N
N
N
NH
O
f1 (ppm)

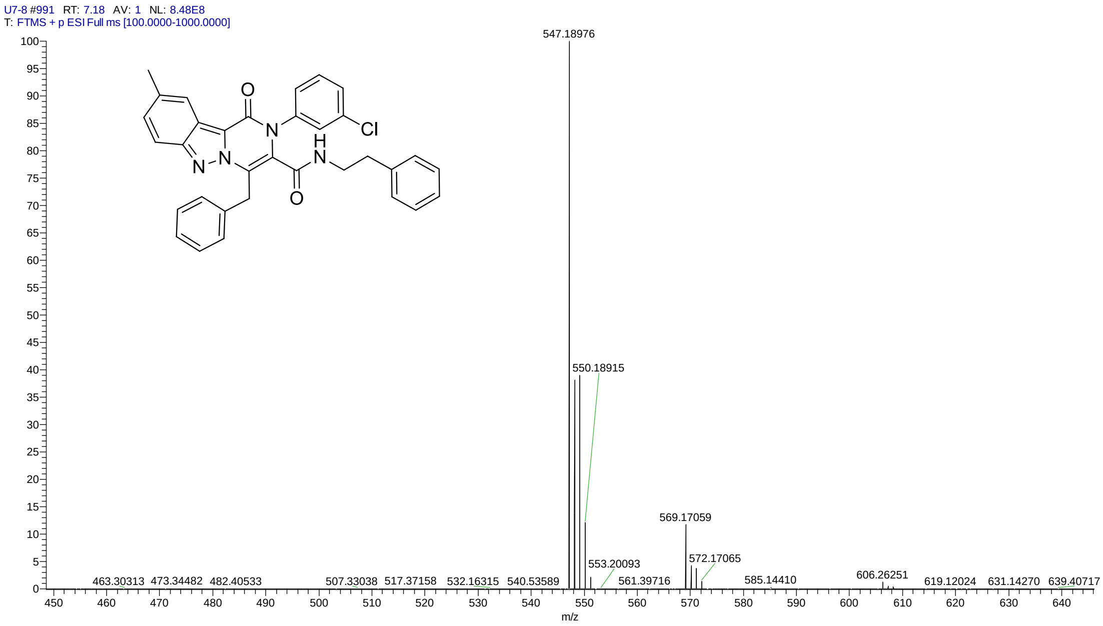

U7-8 #991 RT: 7.18 AV: 1 NL: 8.48E8
T: FTMS + p ESI Full ms [100.0000-1000.0000]
547.18976
550.18915
569.17059
572.17065
553.20093
463.30313
473.34482
482.40533
507.33038
517.37158
532.16315
540.53589
561.39716
585.14410
606.26251
619.12024
631.14270
639.40717
m/z

Compound **7r**

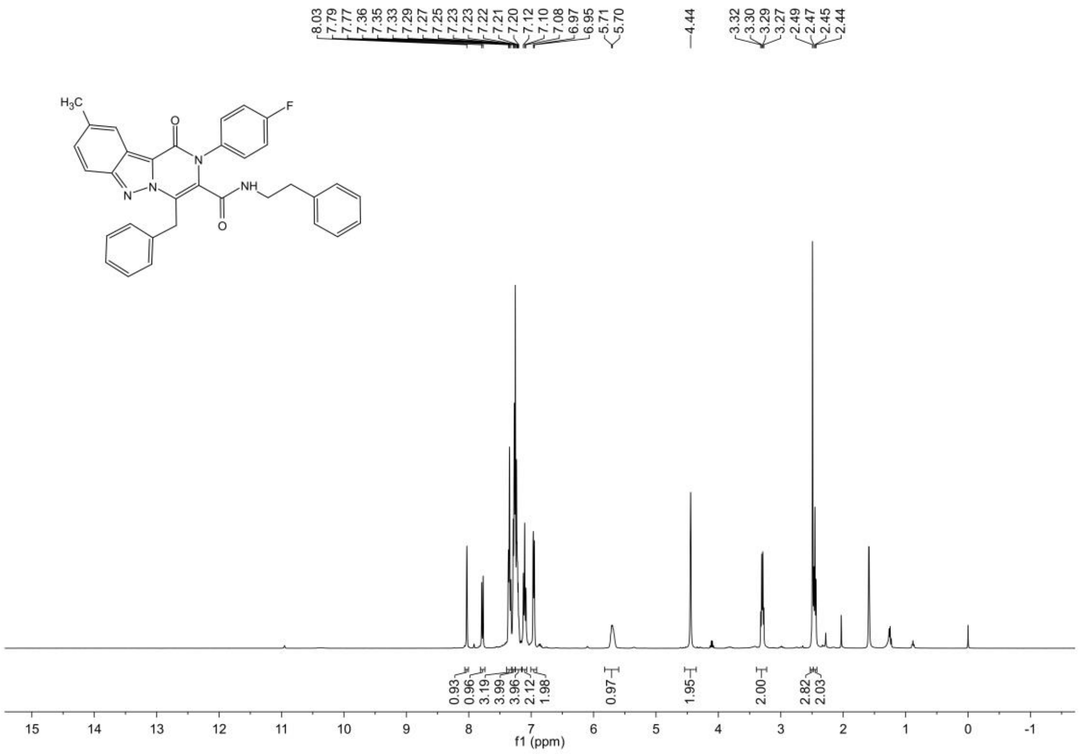
8.03
7.79
7.77
7.36
7.35
7.33
7.29
7.27
7.25
7.23
7.23
7.22
7.21
7.20
7.12
7.10
7.08
6.97
6.95
5.71
5.70
4.44
3.32
3.30
3.29
3.27
2.49
2.47
2.45
2.44
H_3C
O
N
F
N
N
NH
O
0.93
0.96
3.19
3.99
3.96
2.12
1.98
0.97
1.95
2.00
2.82
2.03
15
14
13
12
11
10
9
8
7
6
5
4
3
2
1
0
-1
f1 (ppm)

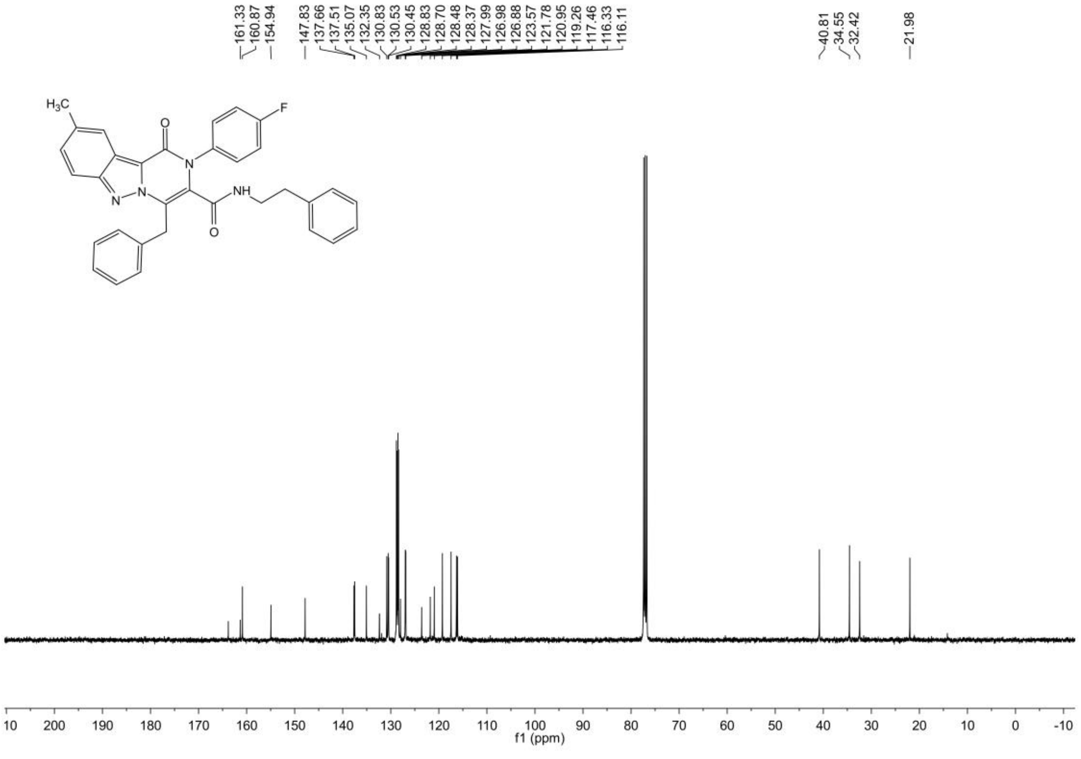
161.33
160.87
154.94
147.83
137.66
137.51
135.07
132.35
130.83
130.53
130.45
128.83
128.70
128.48
128.37
127.99
126.98
126.88
123.57
121.78
120.95
119.26
117.46
116.33
116.11
40.81
34.55
32.42
21.98
H_3C
O
N
F
N
N
NH
O
10
200
190
180
170
160
150
140
130
120
110
100
90
80
70
60
50
40
30
20
10
0
-10
f1 (ppm)

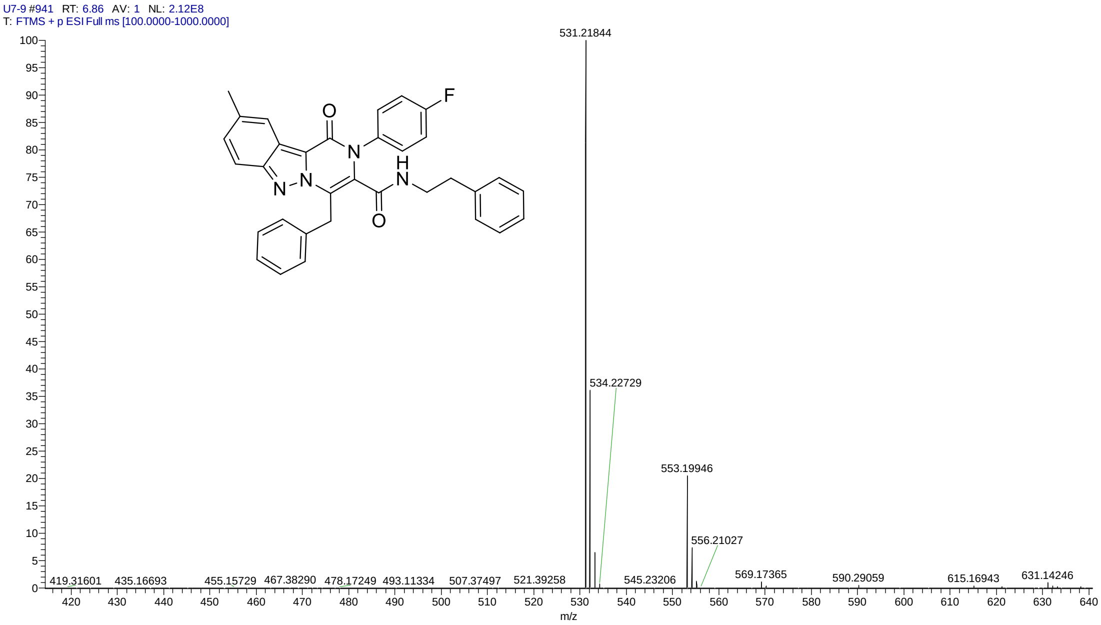
U7-9 #941 RT: 6.86 AV: 1 NL: 2.12E8
T: FTMS + p ESI Full ms [100.0000-1000.0000]
531.21844
534.22729
553.19946
556.21027
m/z

Compound **7s**

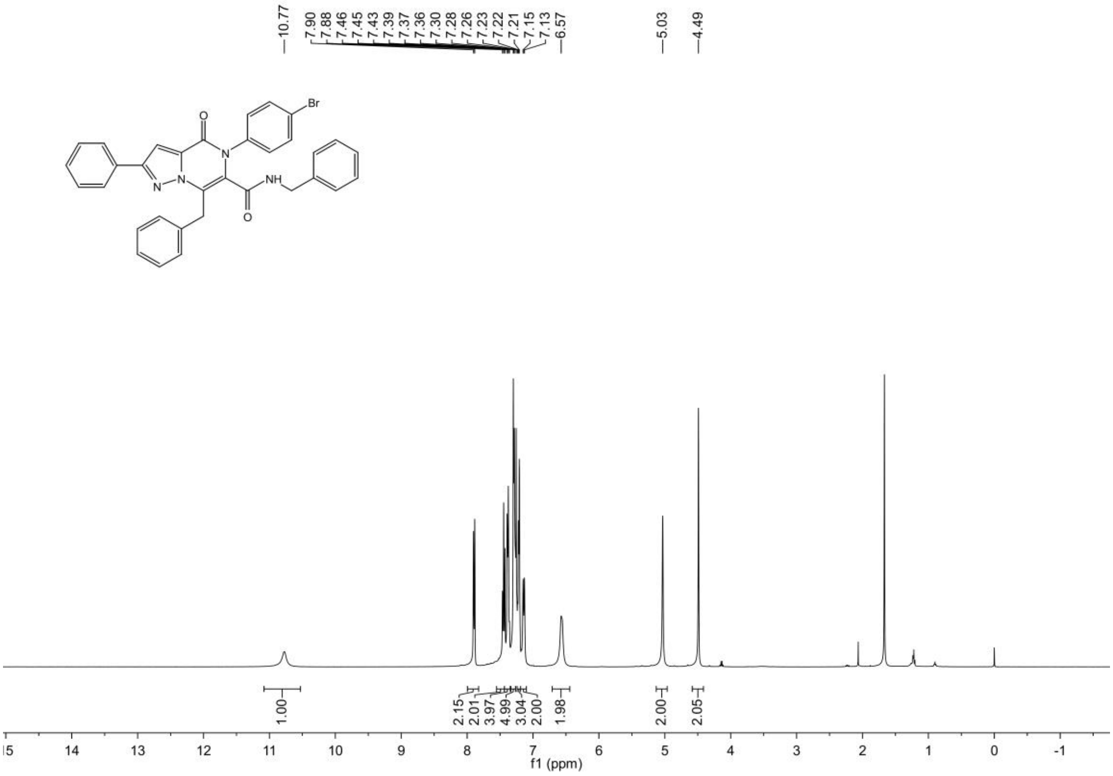
f1 (ppm)

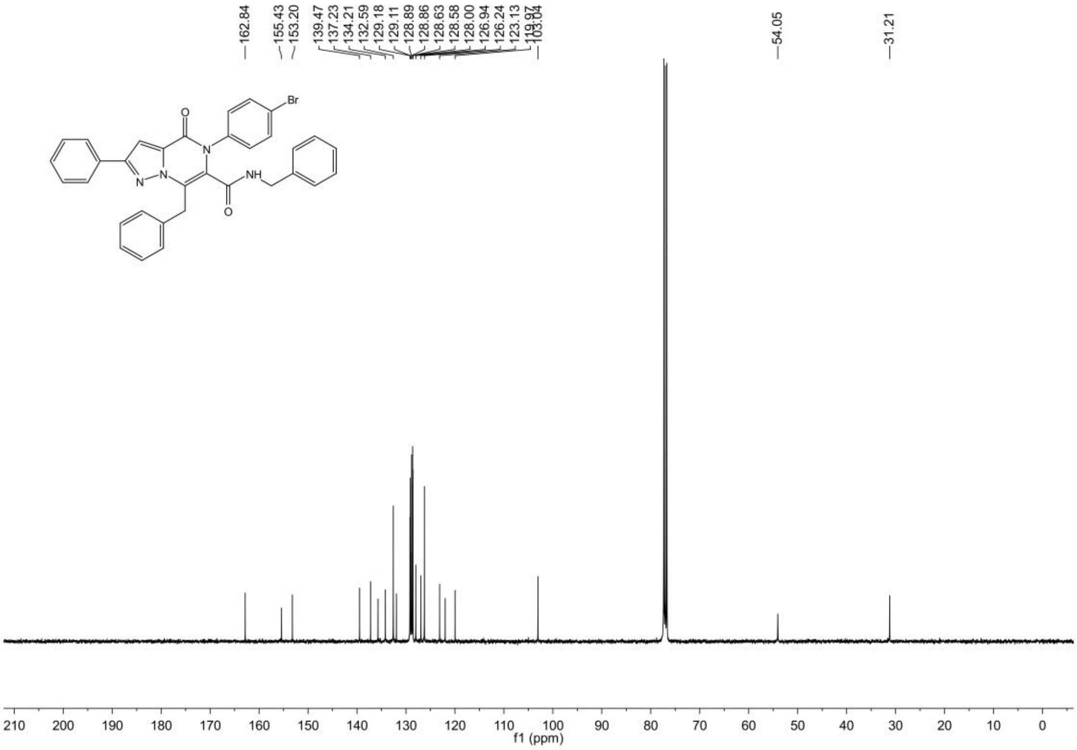
162.84
155.43
153.20
139.47
137.23
134.21
132.59
129.18
129.11
128.89
128.86
128.63
128.58
128.00
126.94
126.24
123.13
103.04
54.05
31.21
Br
O
N
N
N
NH
O
210 200 190 180 170 160 150 140 130 120 110 100 90 80 70 60 50 40 30 20 10 0
f1 (ppm)

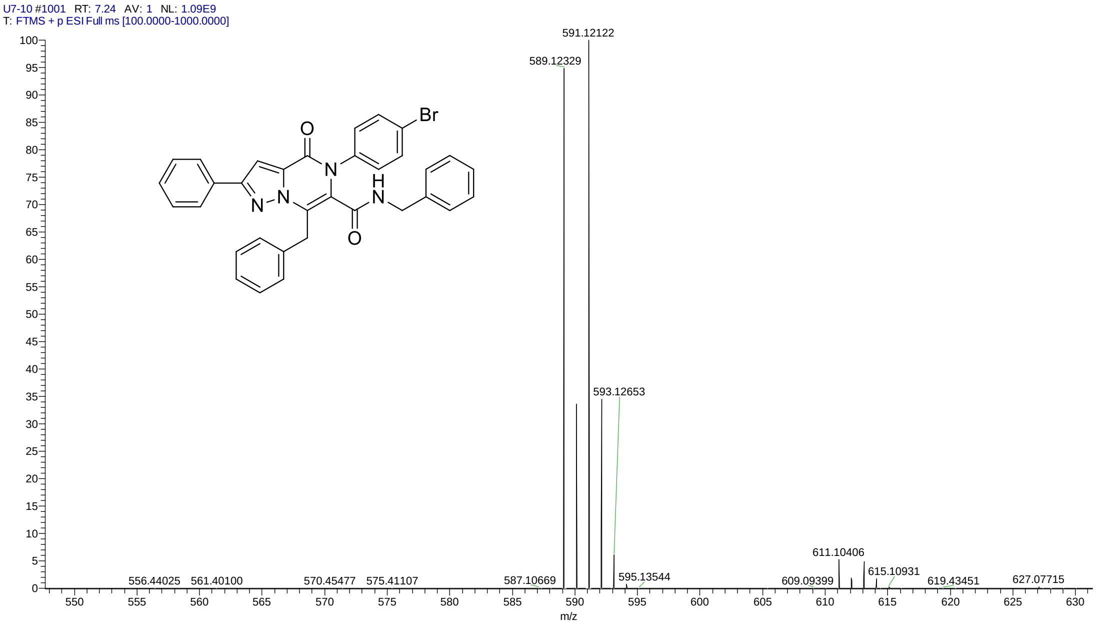
U7-10 #1001 RT: 7.24 AV: 1 NL: 1.09E9
T: FTMS + p ESI Full ms [100.0000-1000.0000]
591.12122
589.12329
593.12653
595.13544
611.10406
615.10931
556.44025
561.40100
570.45477
575.41107
587.10669
609.09399
619.43451
627.07715
Br
O
N
H
N
N
N
O
m/z

Compound **11a**

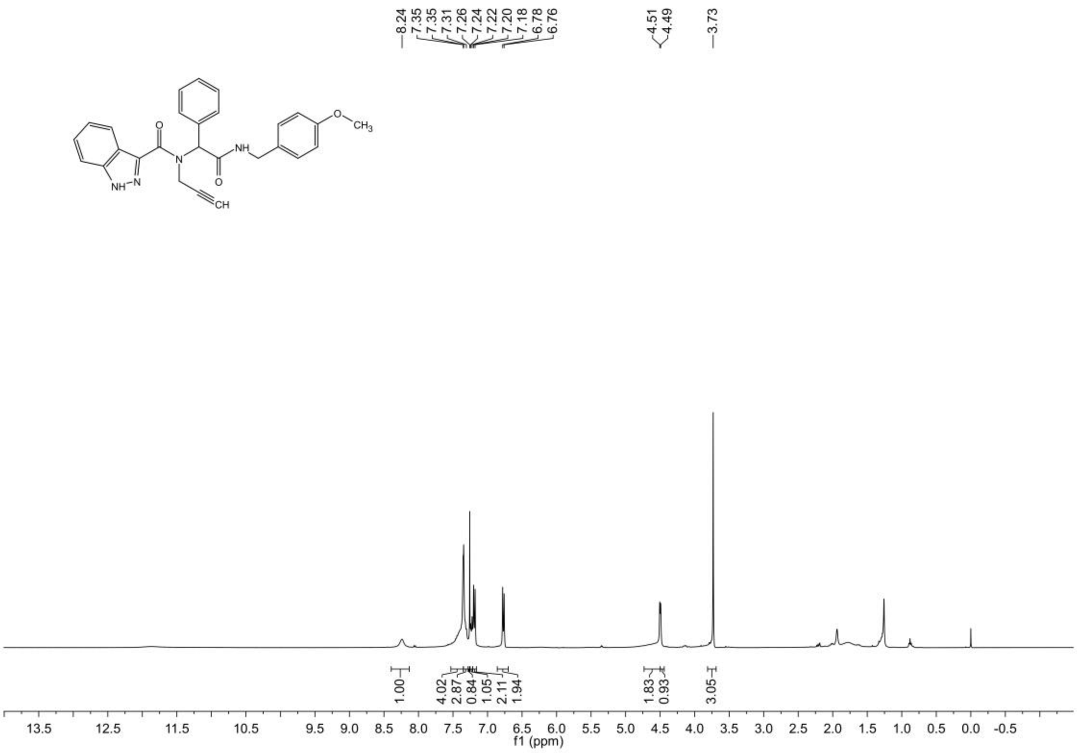

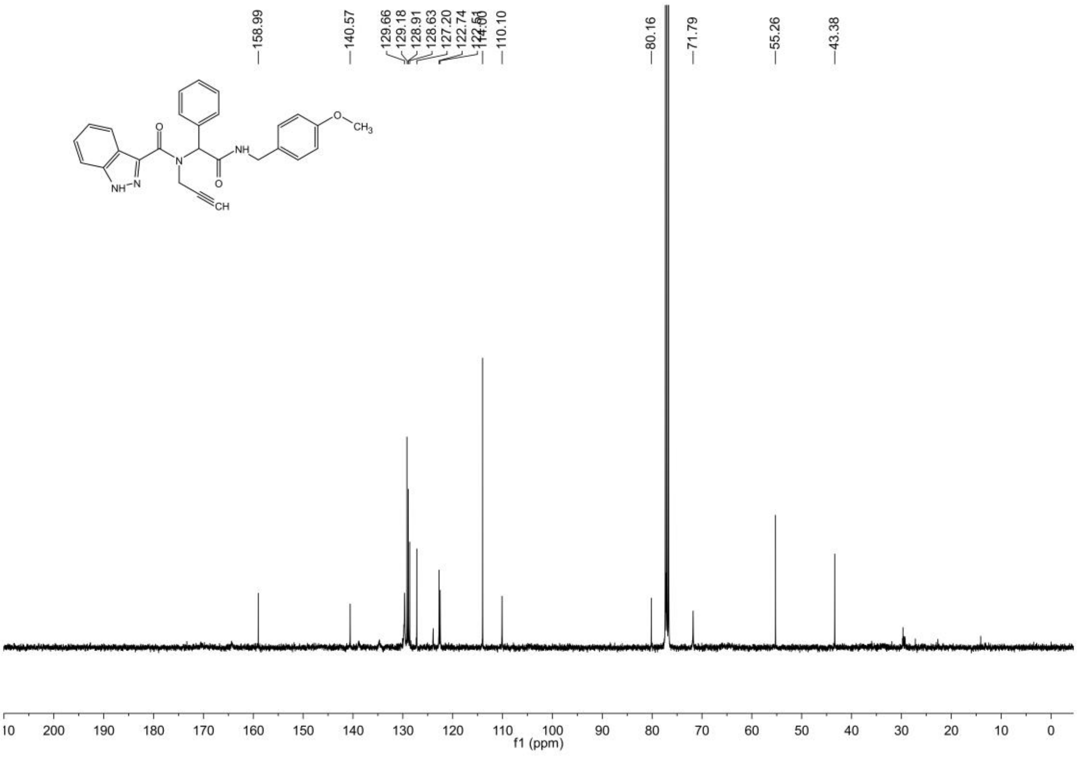

Compound **14a**

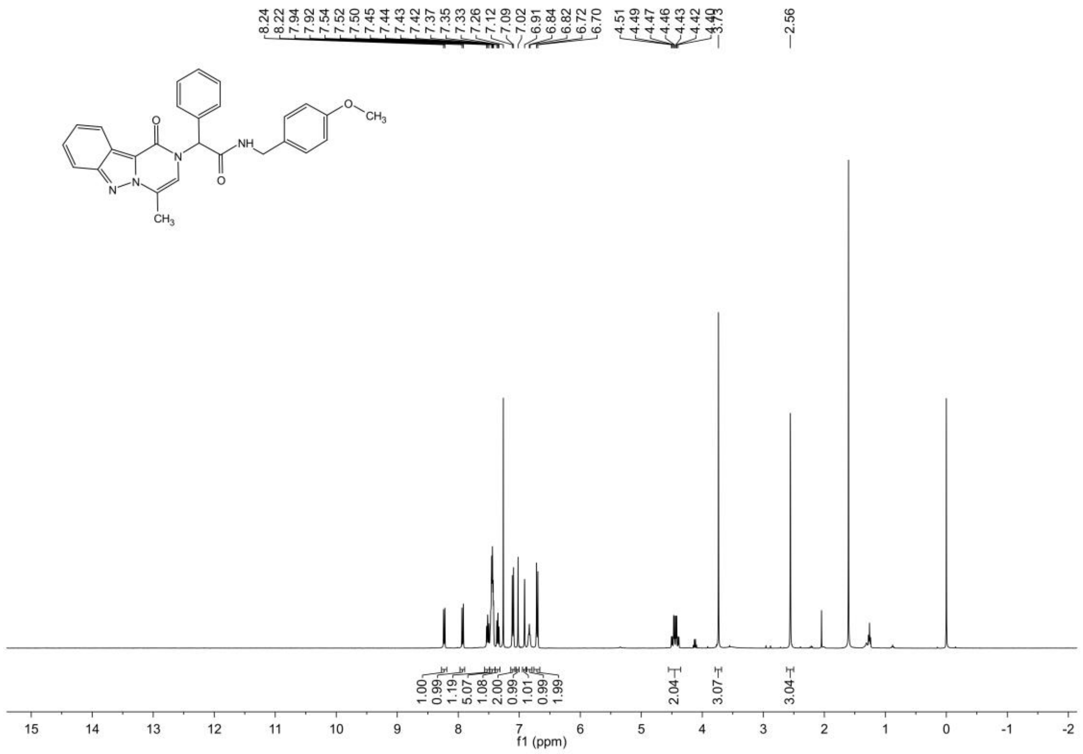

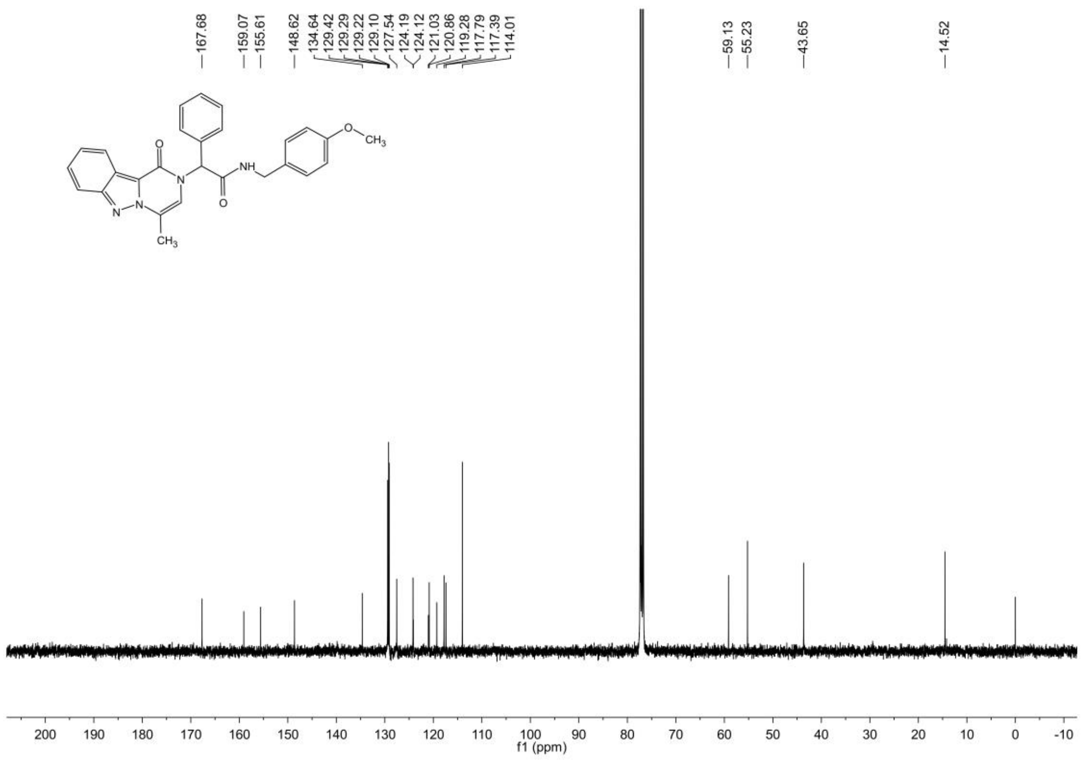

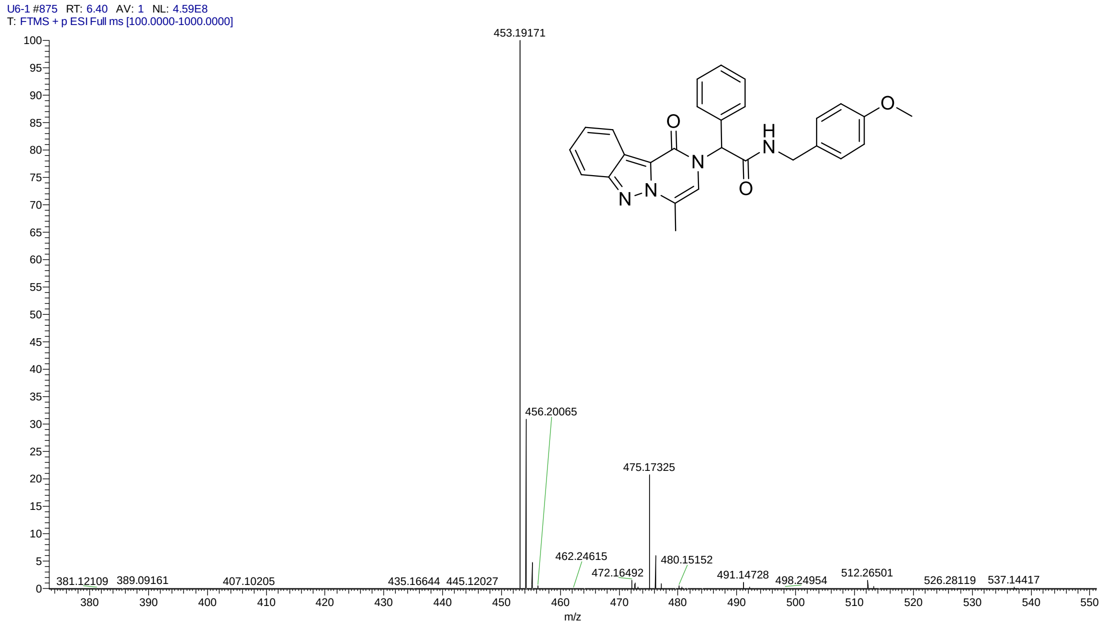

Compound **14b**

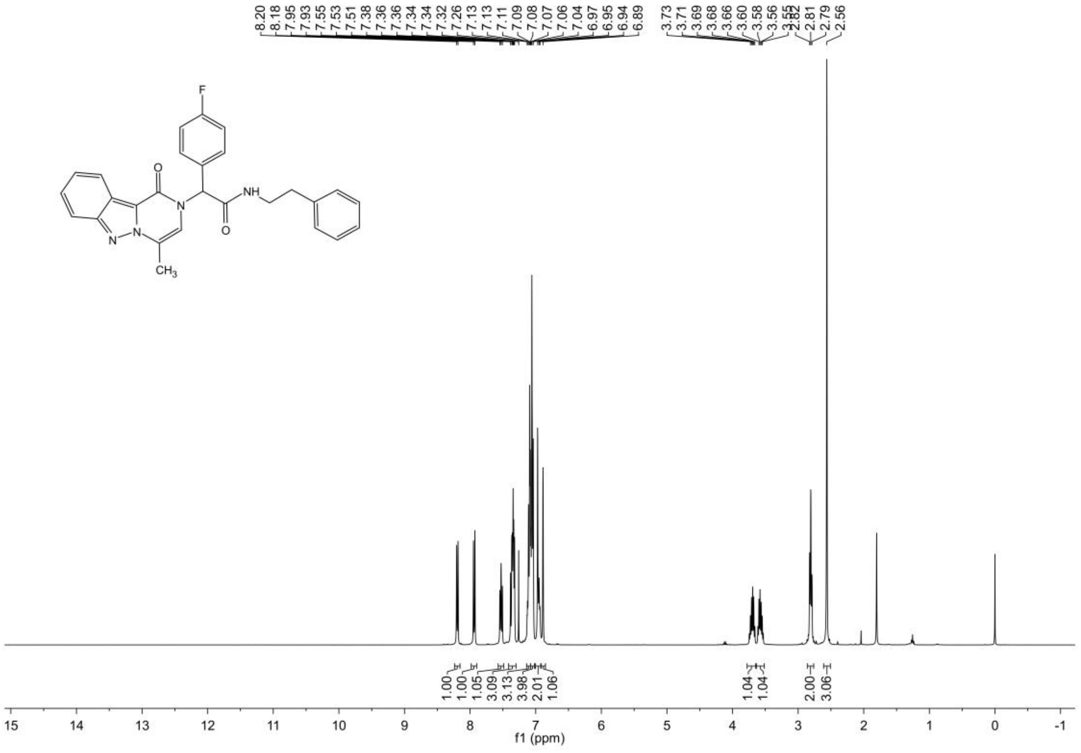

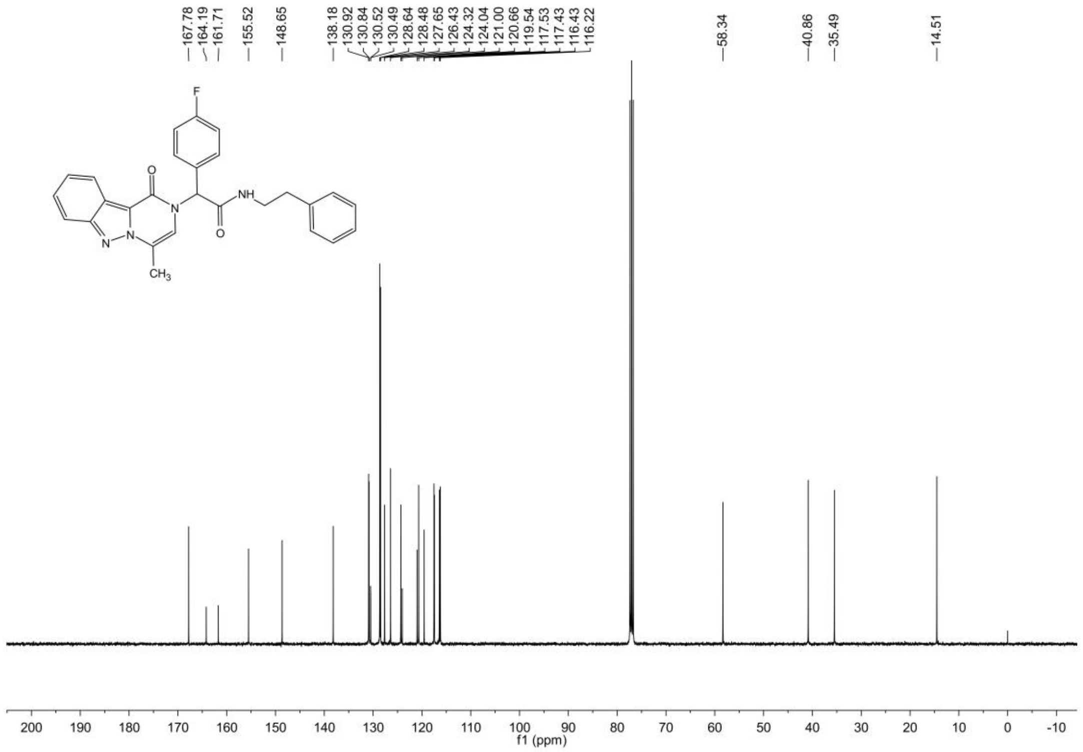
167.78
164.19
161.71
155.52
148.65
138.18
130.92
130.84
130.52
130.49
128.64
128.48
127.65
126.43
124.32
124.04
121.00
120.66
119.54
117.53
117.43
116.43
116.22
58.34
40.86
35.49
14.51
f1 (ppm)

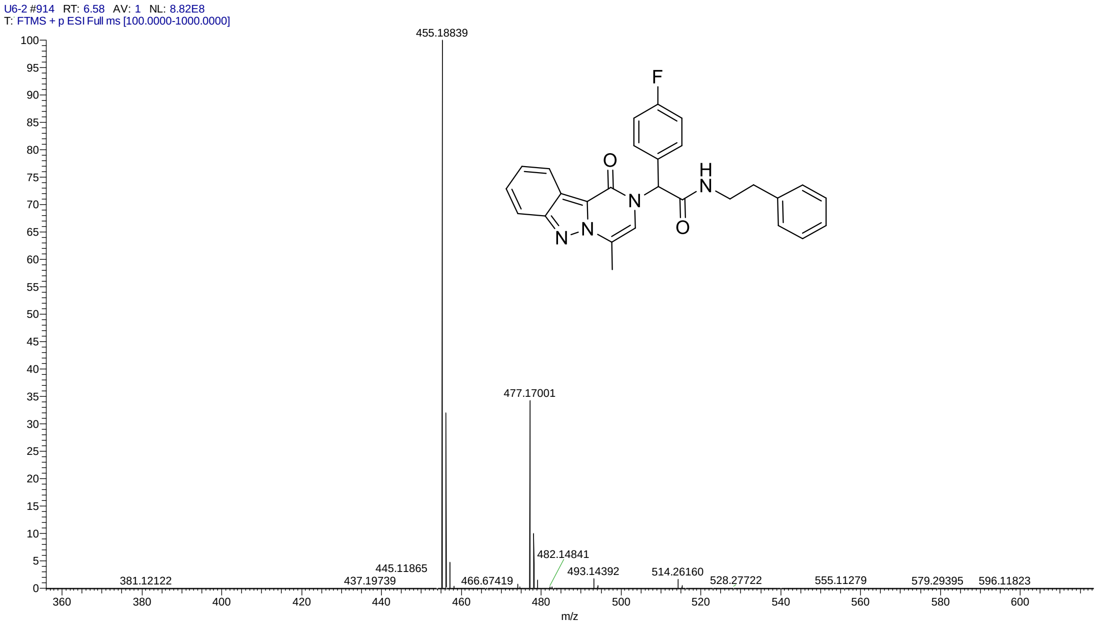
U6-2 #914 RT: 6.58 AV: 1 NL: 8.82E8
T: FTMS + p ESI Full ms [100.0000-1000.0000]
455.18839
477.17001
482.14841
493.14392
445.11865
437.19739
466.67419
381.12122
514.26160
528.27722
555.11279
579.29395
596.11823
m/z

Compound **14c**

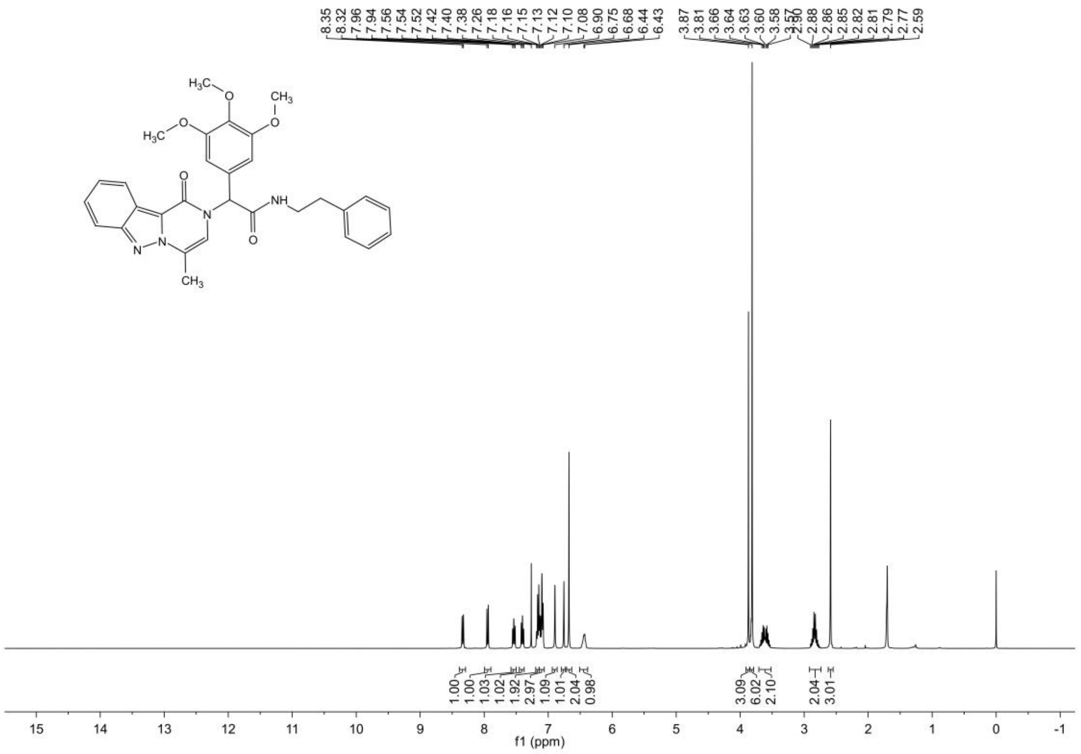

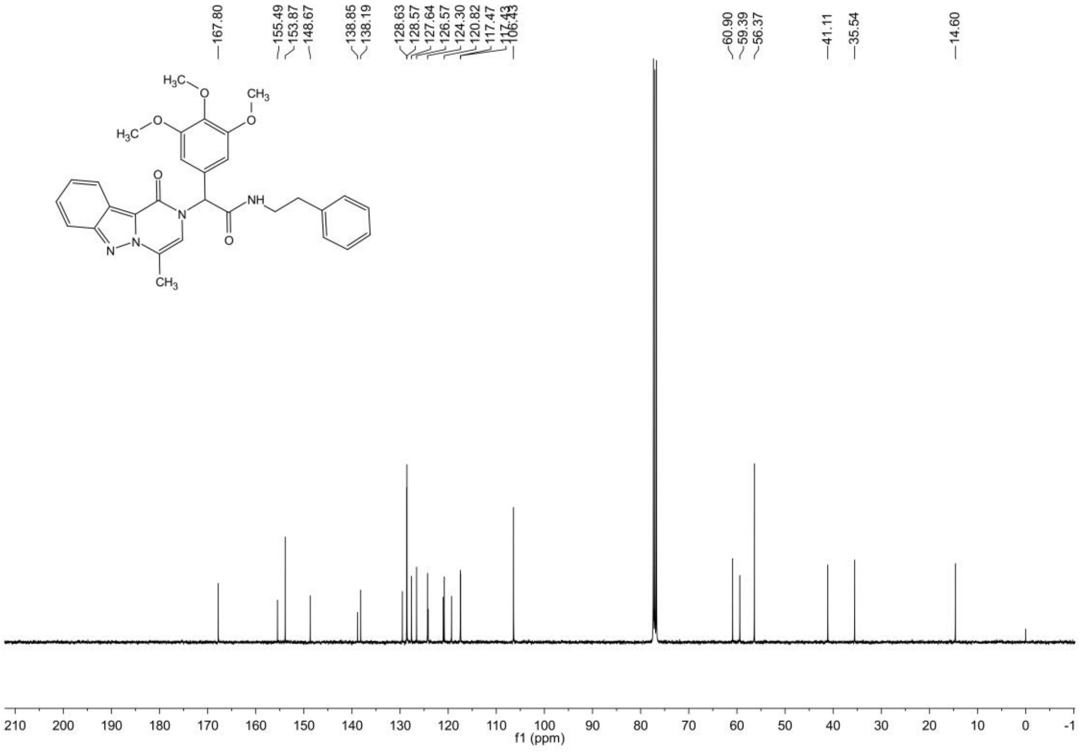

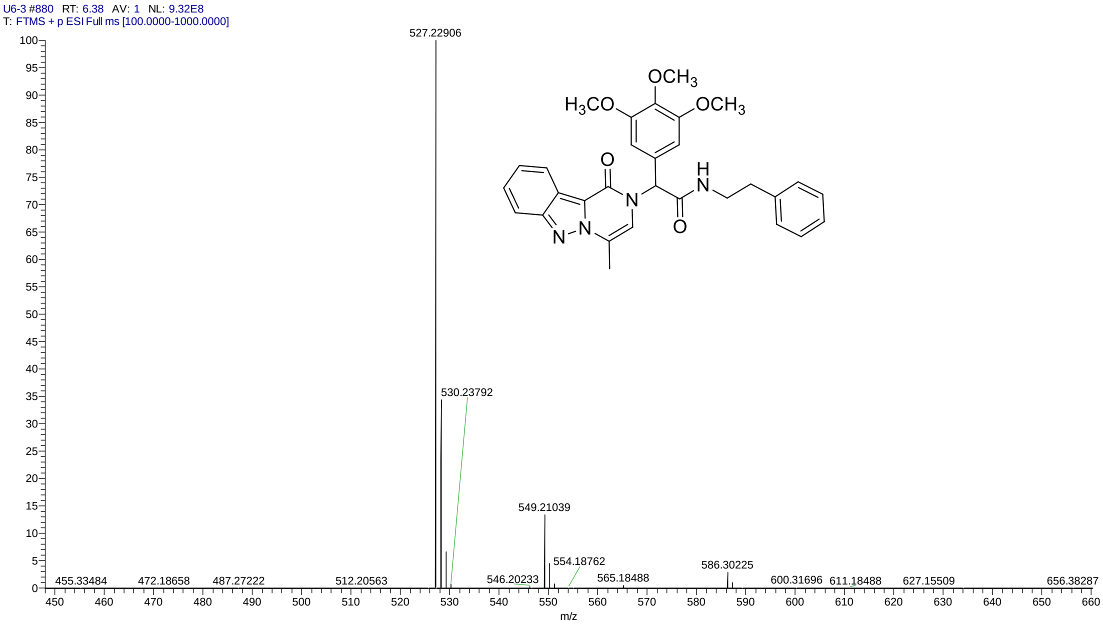

Compound **14d**

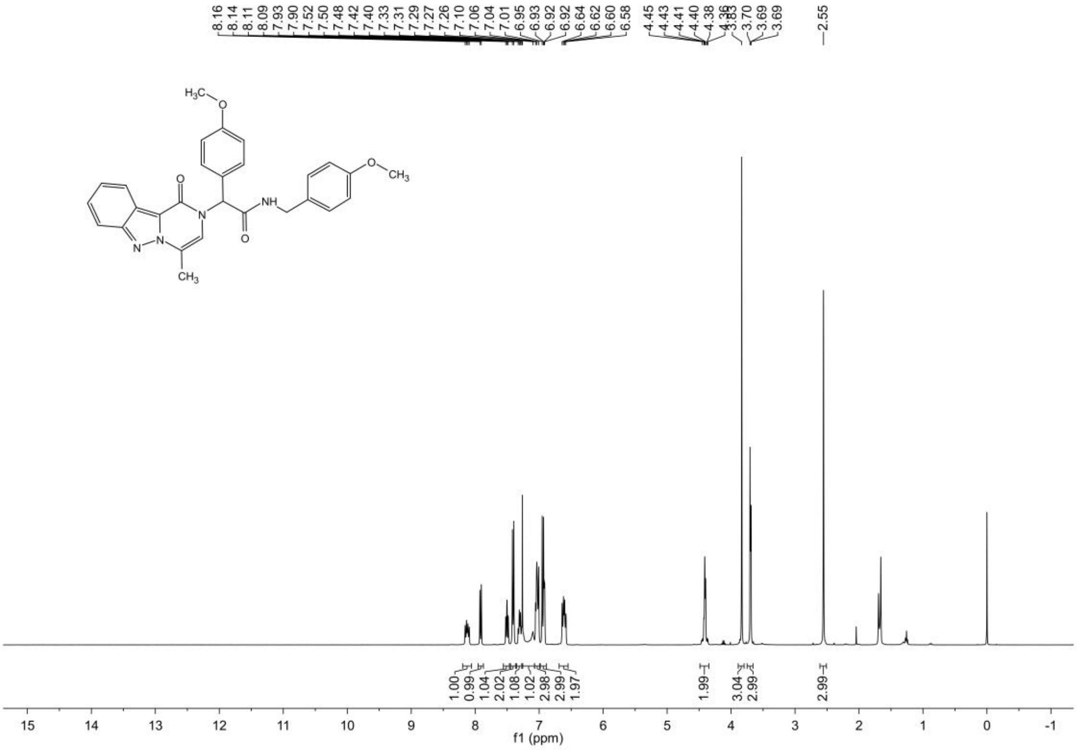

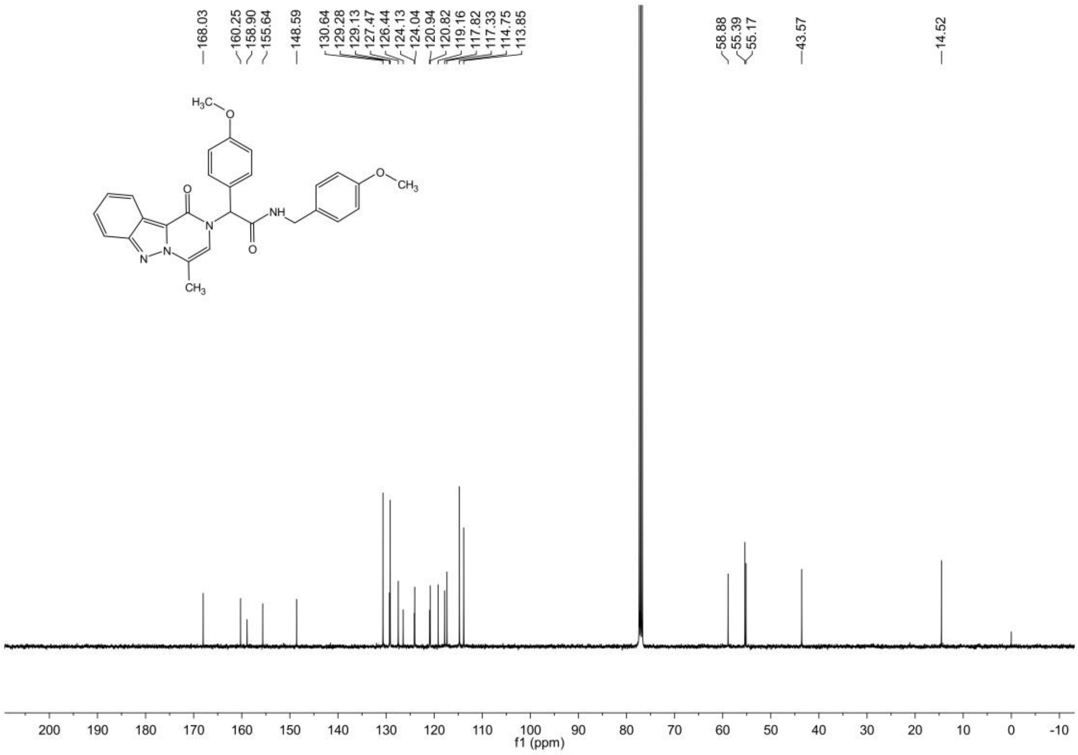
168.03
160.25
158.90
155.64
148.59
130.64
129.28
129.13
127.47
126.44
124.13
124.04
120.94
120.82
119.16
117.82
117.33
114.75
113.85
58.88
55.39
55.17
43.57
14.52
f1 (ppm)

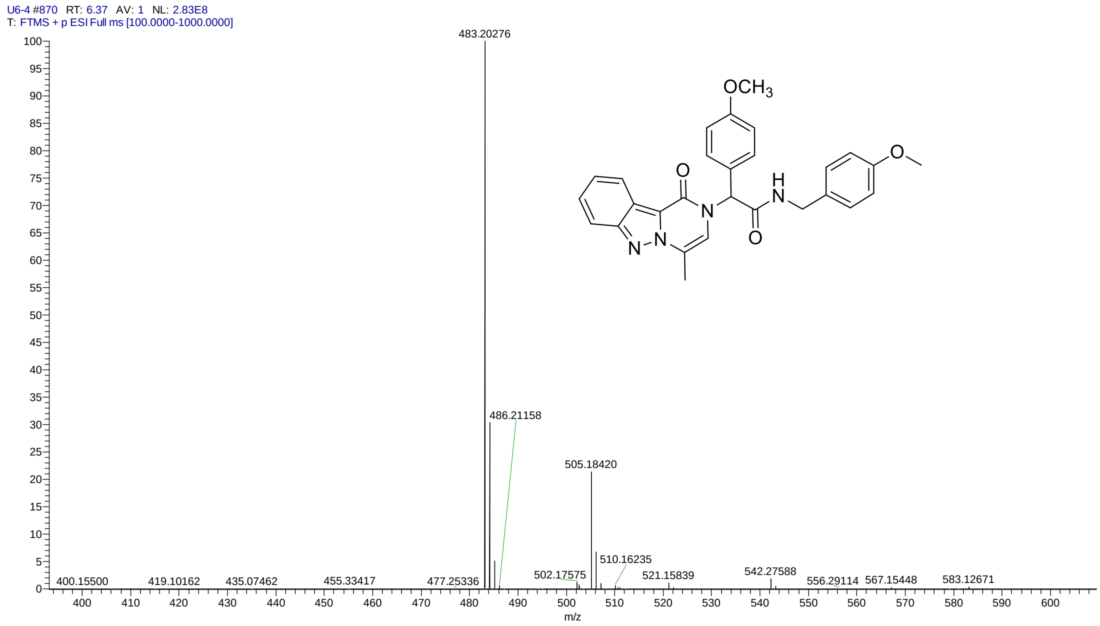
U6-4 #870 RT: 6.37 AV: 1 NL: 2.83E8
T: FTMS + p ESI Full ms [100.0000-1000.0000]
483.20276
486.21158
505.18420
510.16235
400.15500
419.10162
435.07462
455.33417
477.25336
502.17575
521.15839
542.27588
556.29114
567.15448
583.12671
m/z

Compound **14e**

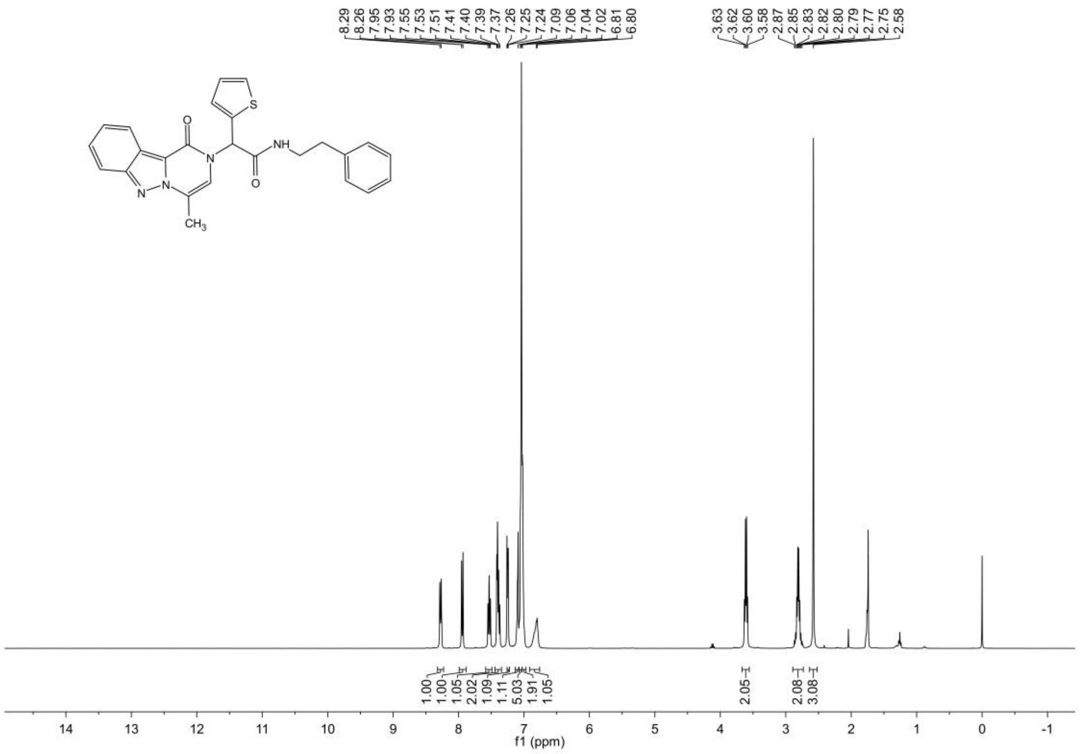

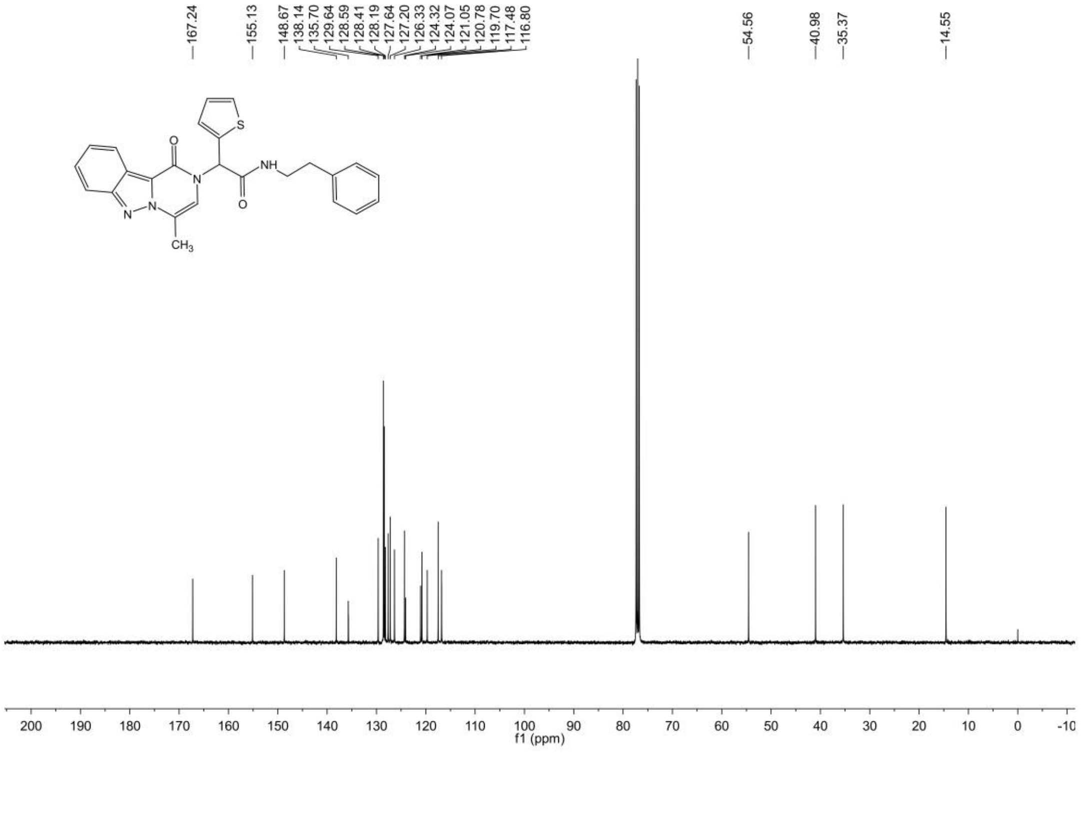

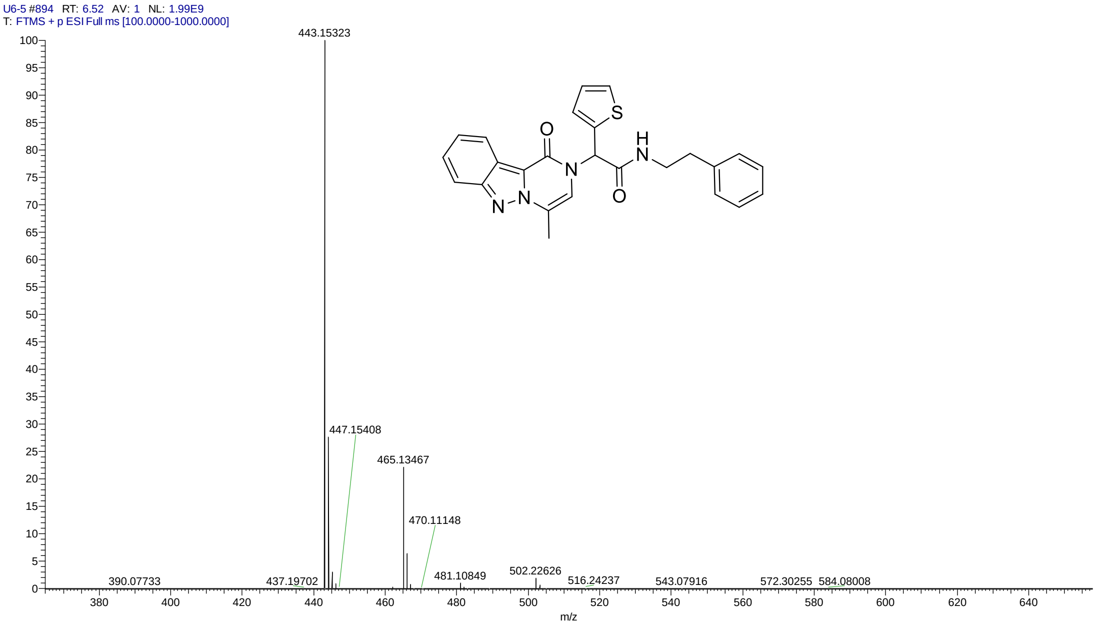

Compound **14f**

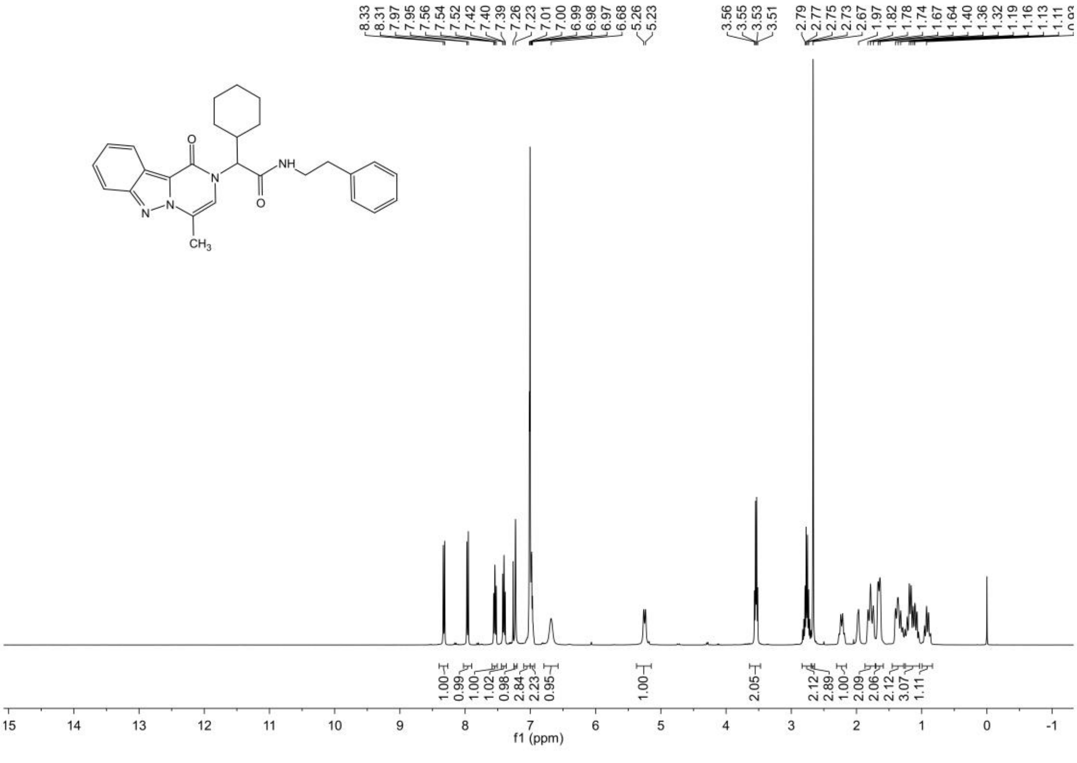

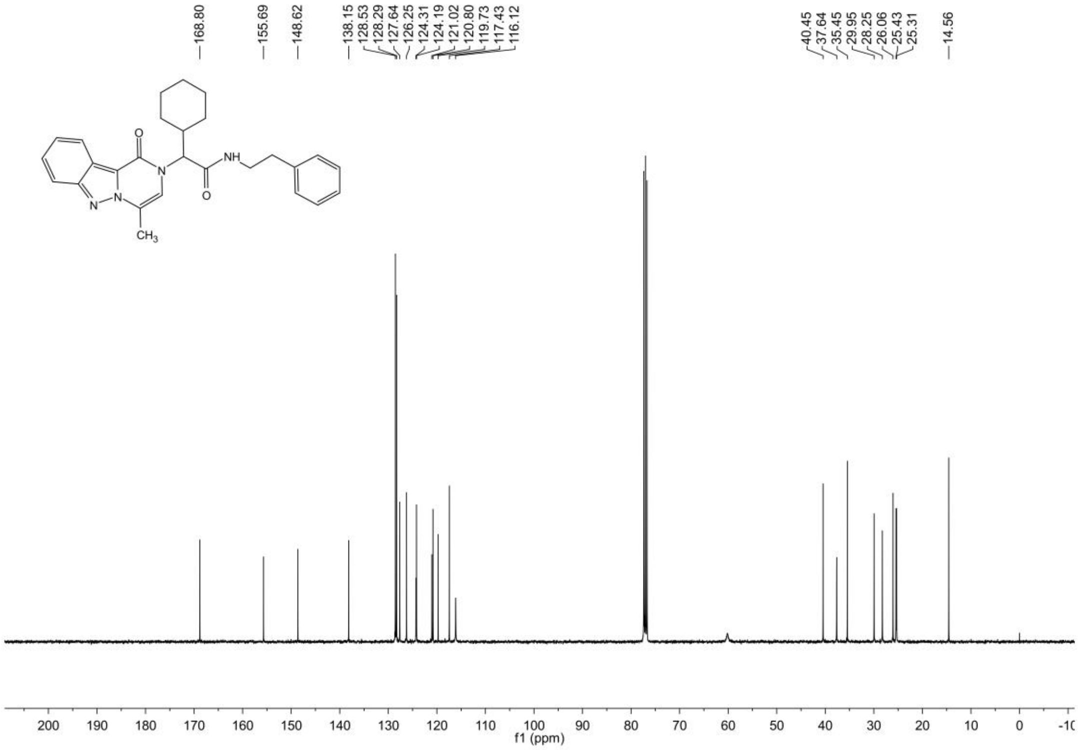
168.80
155.69
148.62
138.15
128.53
128.29
127.64
126.25
124.31
124.19
121.02
120.80
119.73
117.43
116.12
40.45
37.64
35.45
29.95
28.25
26.06
25.43
25.31
14.56
f1 (ppm)

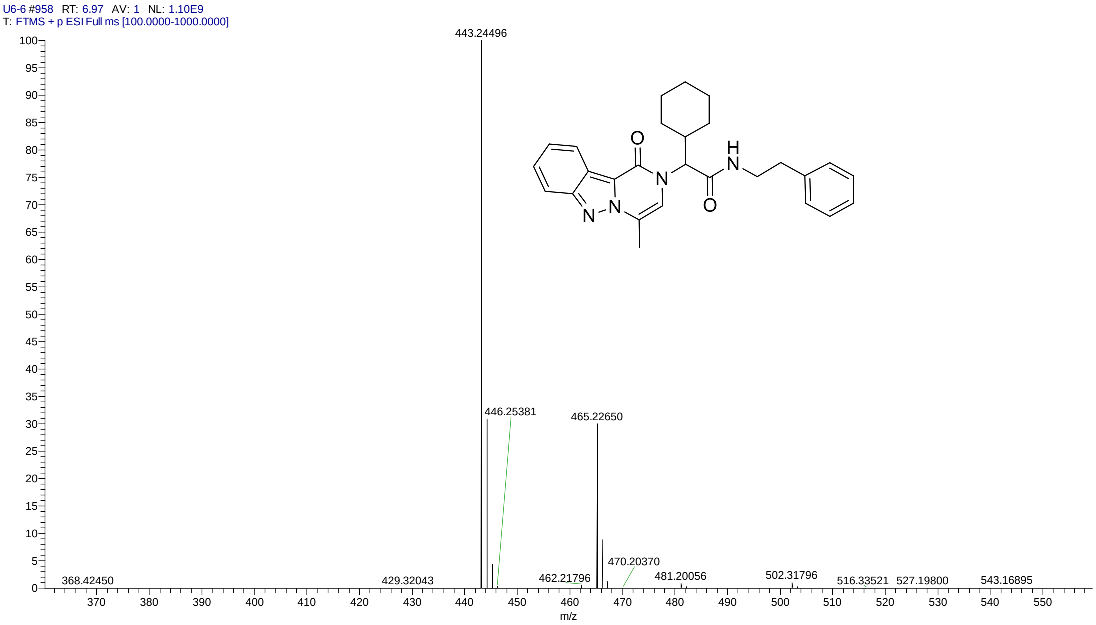
U6-6 #958 RT: 6.97 AV: 1 NL: 1.10E9
T: FTMS + p ESI Full ms [100.0000-1000.0000]
443.24496
446.25381
465.22650
470.20370
368.42450
429.32043
462.21796
481.20056
502.31796
516.33521
527.19800
543.16895
m/z

Compound **14g**

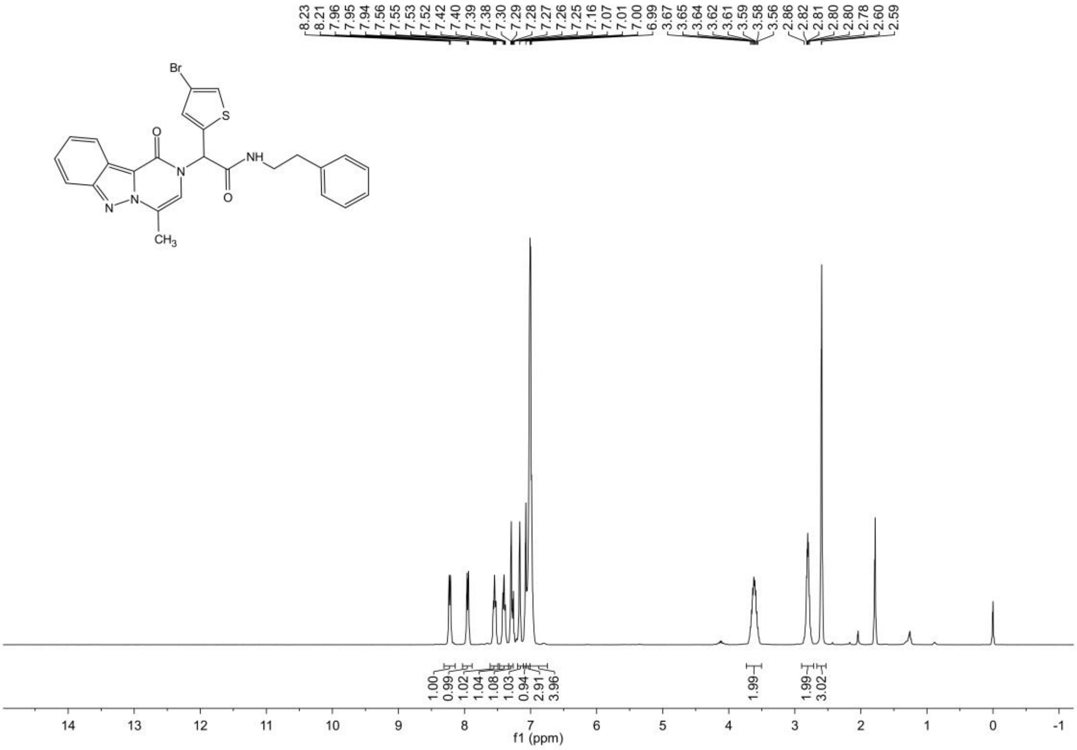

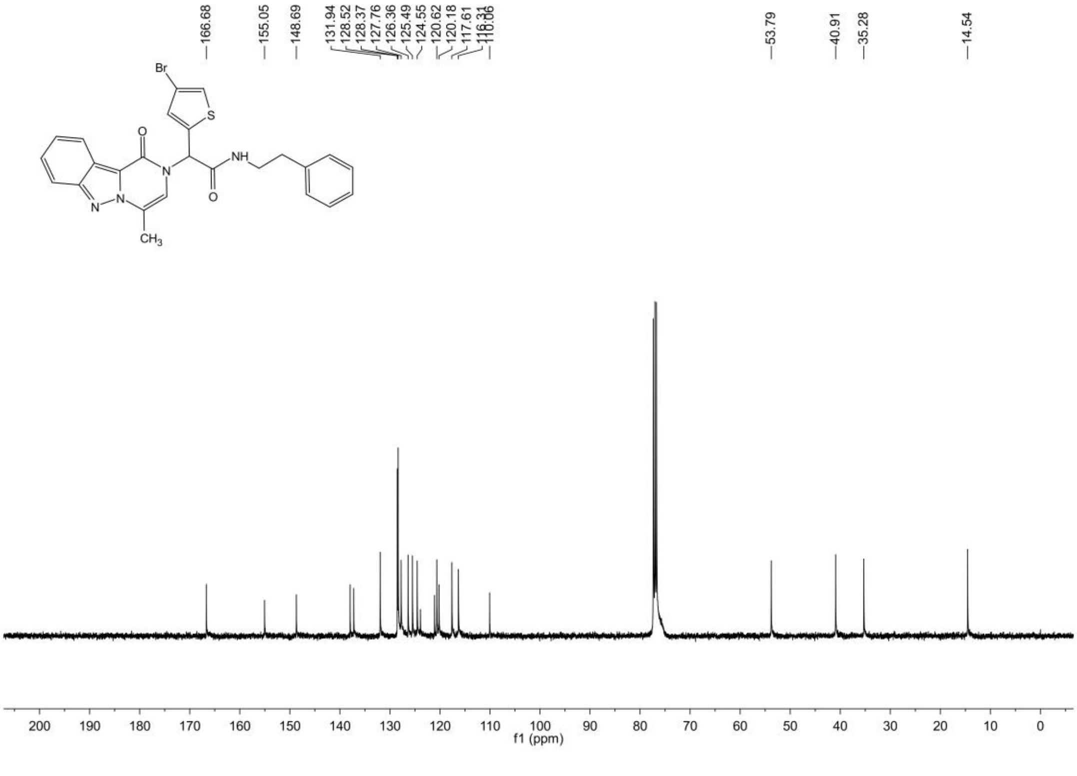

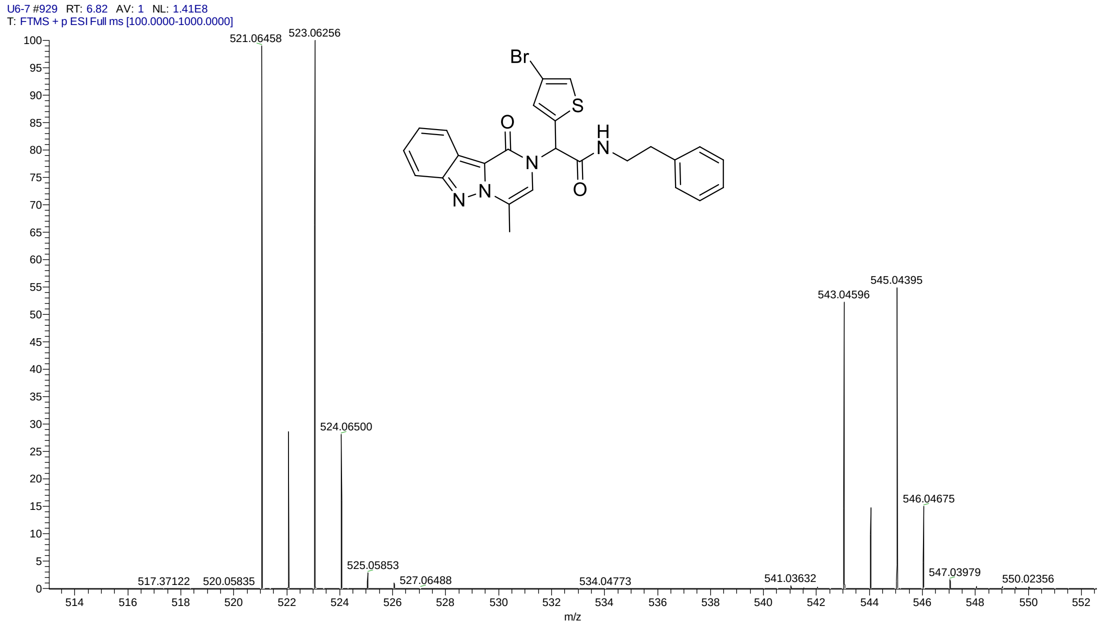

Compound **14h**

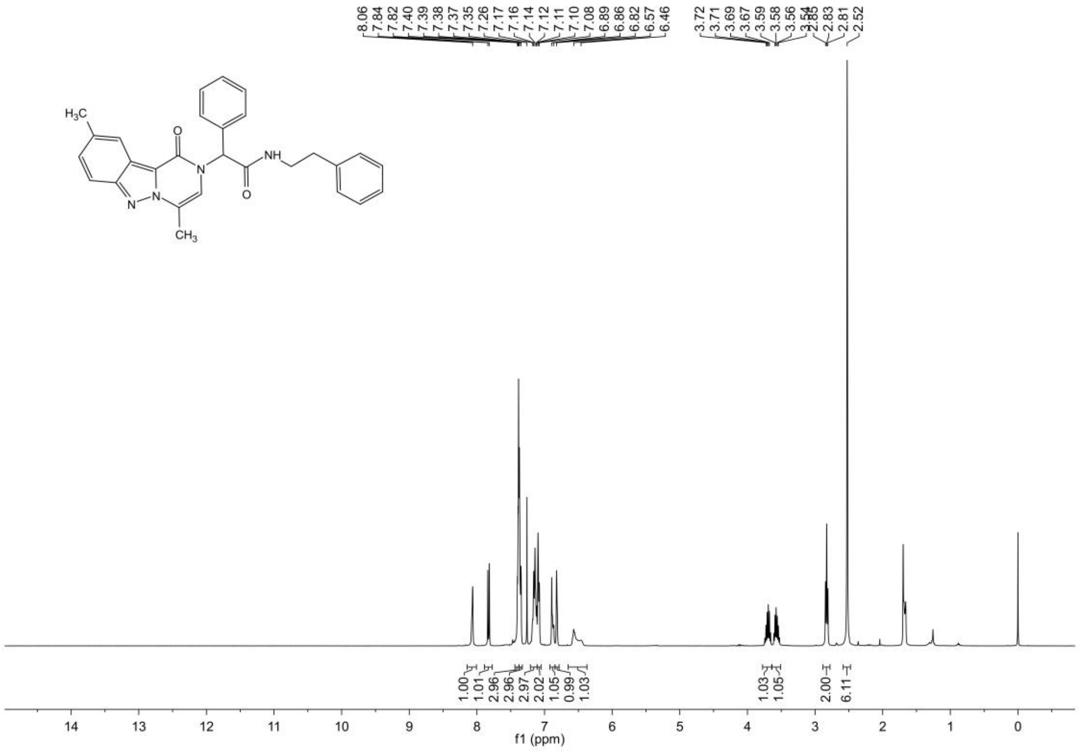

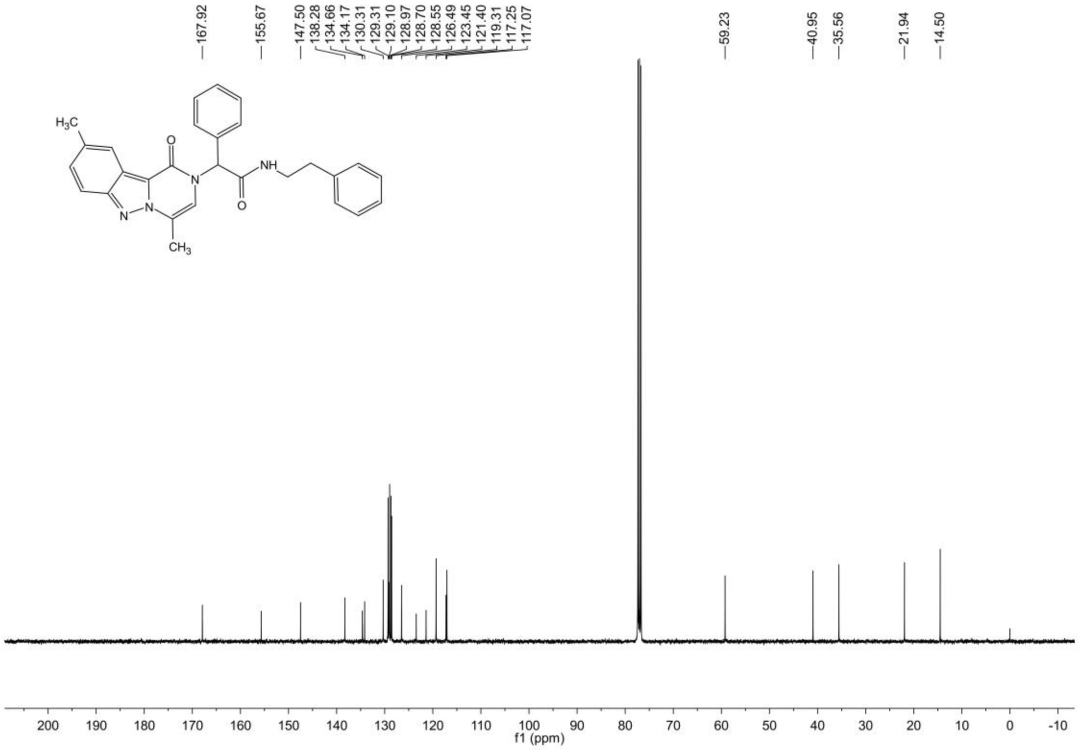
167.92
155.67
147.50
138.28
134.66
134.17
130.31
129.31
129.10
128.97
128.70
128.55
126.49
123.45
121.40
119.31
117.25
117.07
59.23
40.95
35.56
21.94
14.50
f1 (ppm)

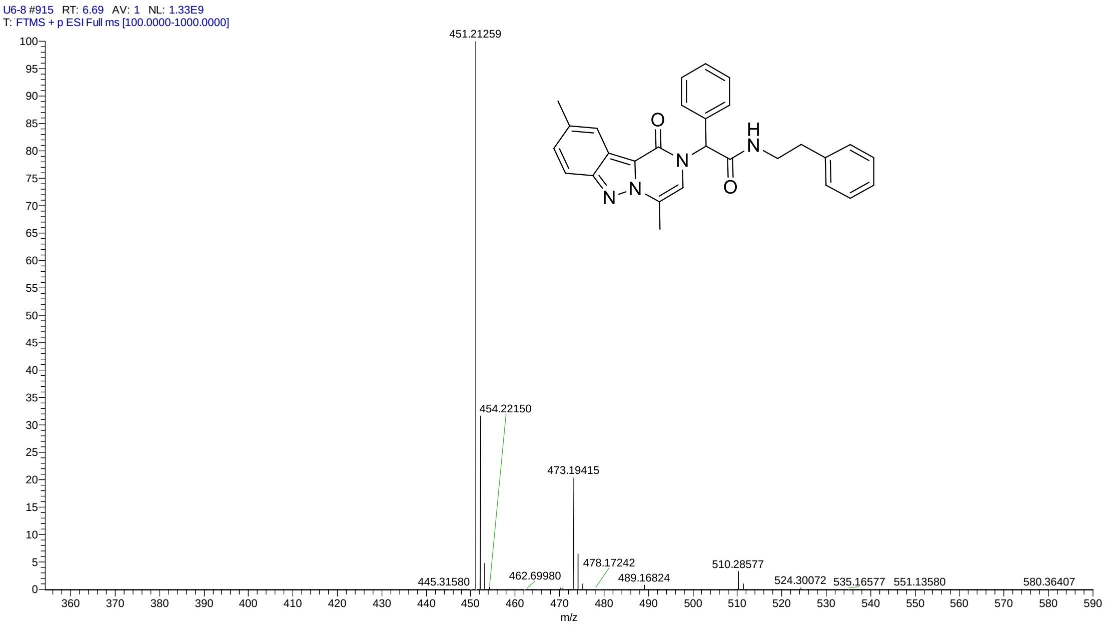
U6-8 #915 RT: 6.69 AV: 1 NL: 1.33E9
T: FTMS + p ESI Full ms [100.0000-1000.0000]
451.21259
454.22150
473.19415
445.31580
462.69980
478.17242
489.16824
510.28577
524.30072
535.16577
551.13580
580.36407
m/z

Compound **14i**

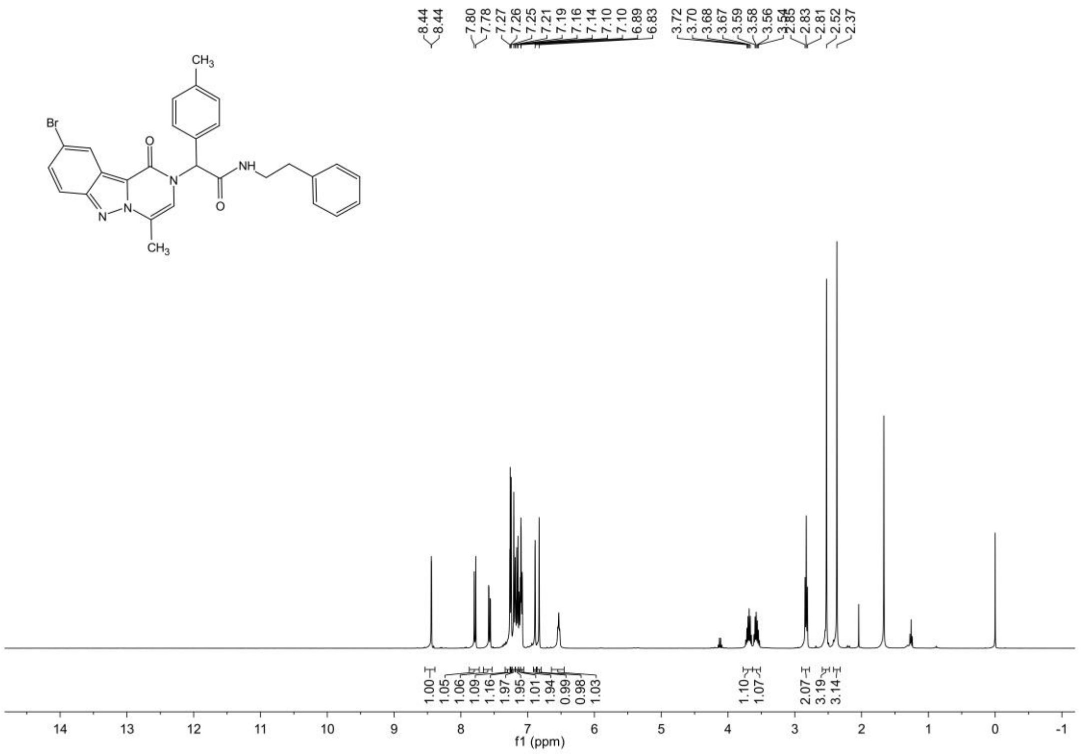

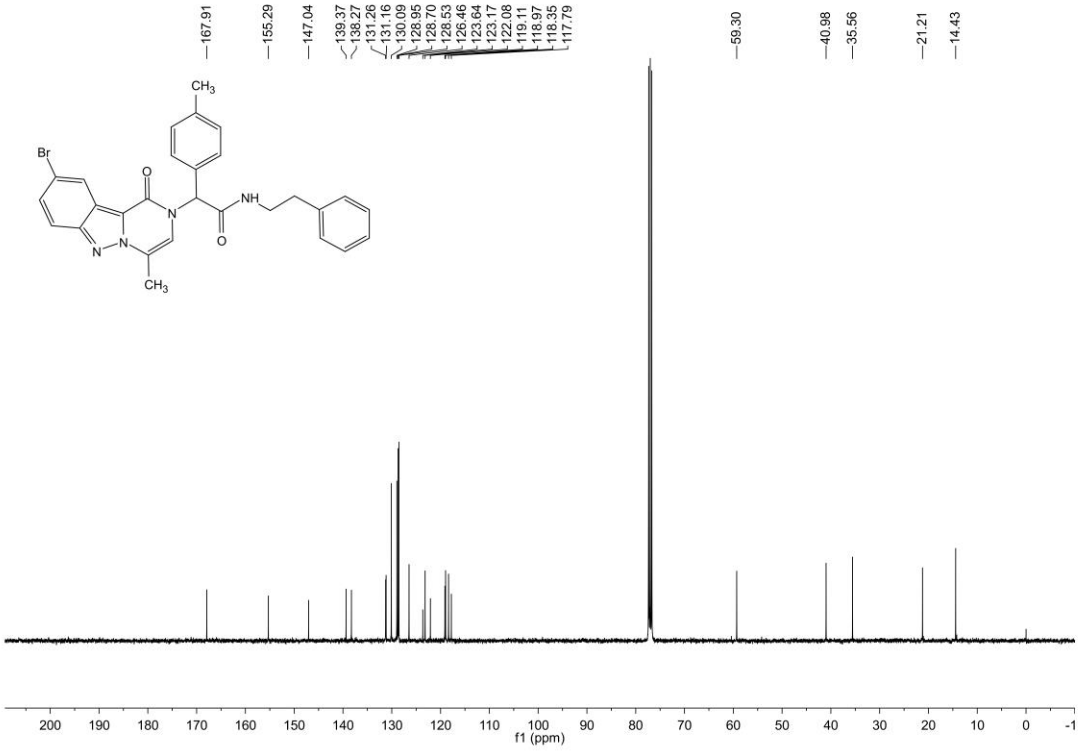

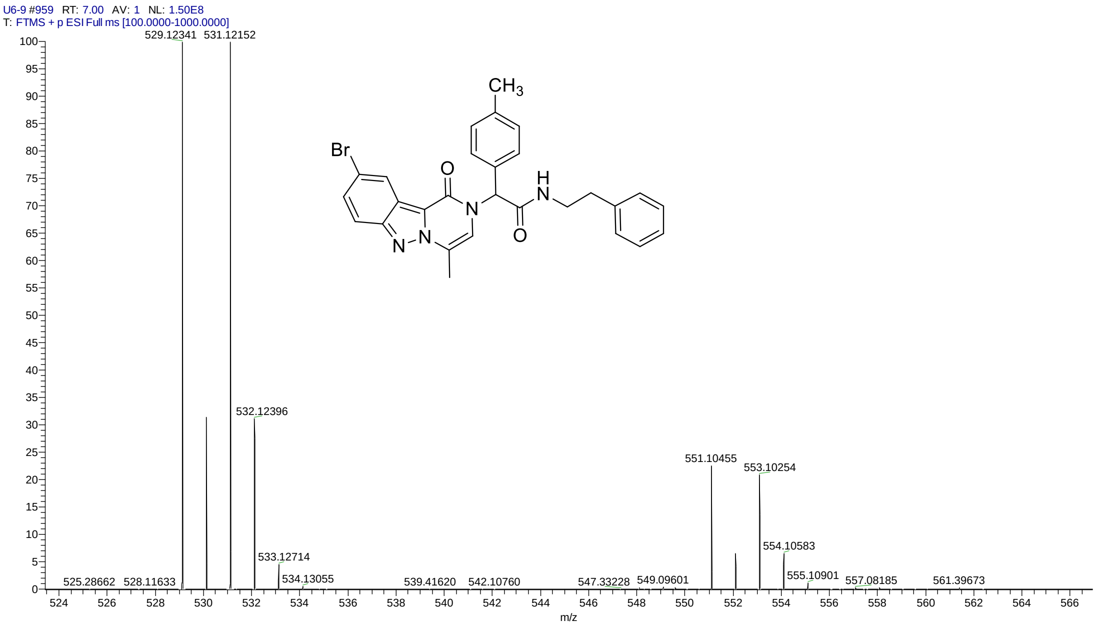

Compound **14j**

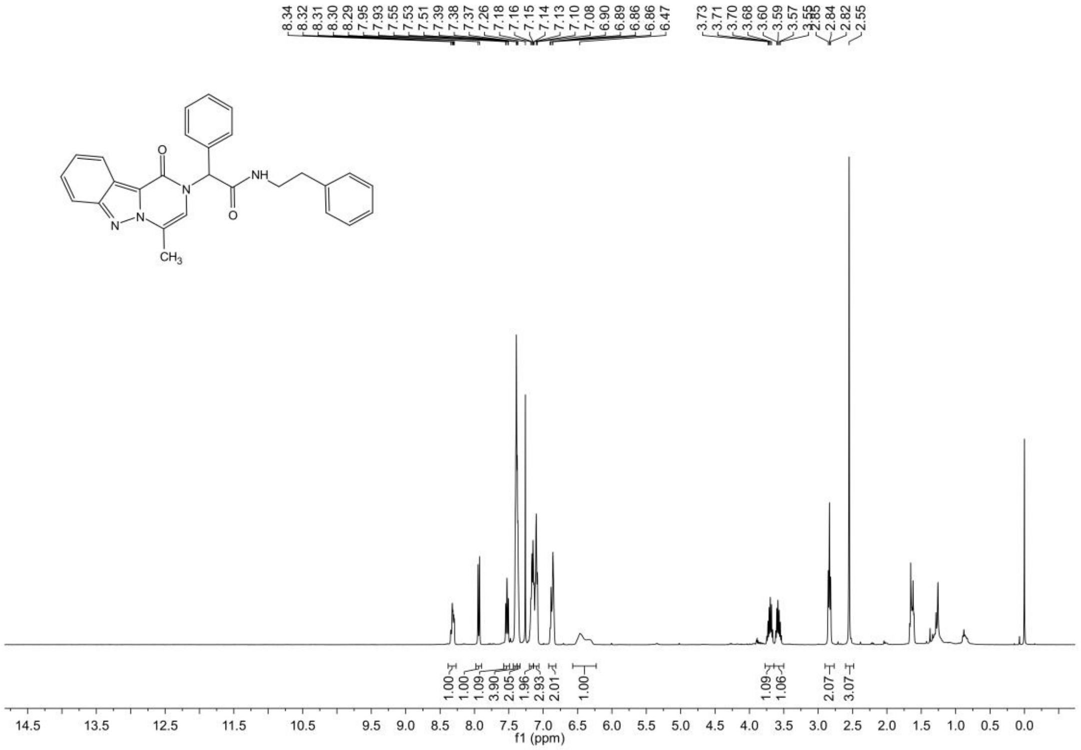

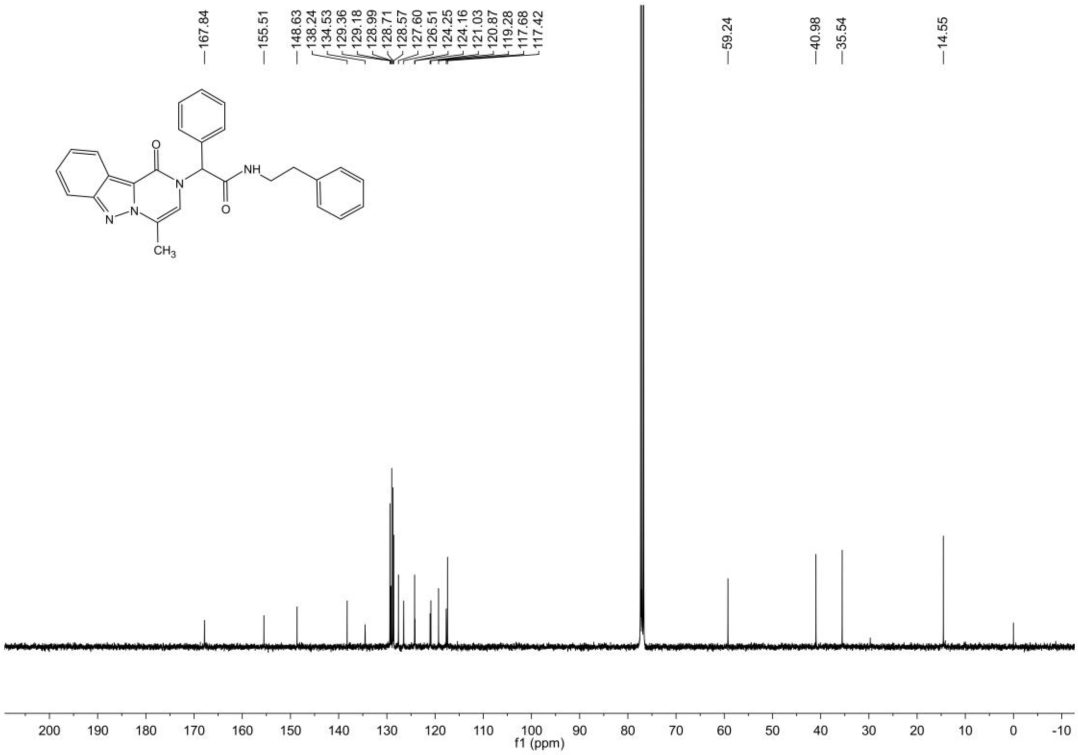
167.84
155.51
148.63
138.24
134.53
129.36
129.18
128.99
128.71
128.57
127.60
126.51
124.25
124.16
121.03
120.87
119.28
117.68
117.42
59.24
40.98
35.54
14.55
O
N
NH
N
N
O
CH3
f1 (ppm)

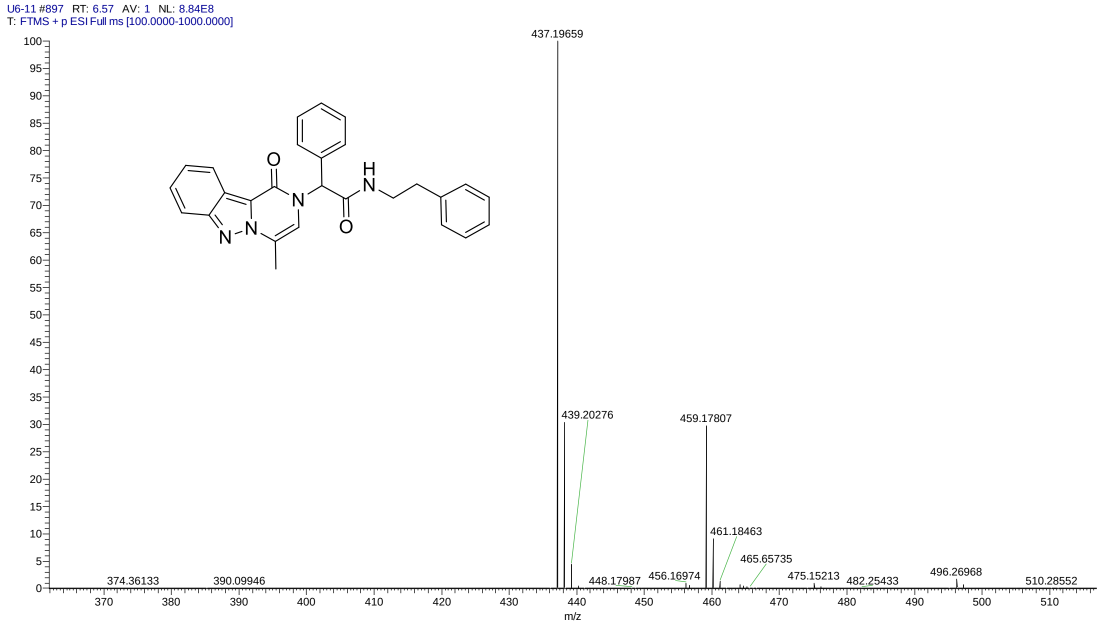
U6-11 #897 RT: 6.57 AV: 1 NL: 8.84E8
T: FTMS + p ESI Full ms [100.0000-1000.0000]
437.19659
439.20276
459.17807
461.18463
465.65735
374.36133
390.09946
448.17987
456.16974
475.15213
482.25433
496.26968
510.28552
m/z
